# A review of the melliferous flora of Yucatan peninsula, Mexico, on the basis for the honey production cycle

**DOI:** 10.1186/s13002-024-00681-0

**Published:** 2024-03-25

**Authors:** Donají Zúñiga-Díaz, William Cetzal-Ix, Héctor López-Castilla, Eliana Noguera-Savelli, Iván Tamayo-Cen, Jesús Froylán Martínez-Puc, Saikat Kumar Basu

**Affiliations:** 1 Tecnológico Nacional de México, Instituto Tecnológico de Chiná, Chiná, Campeche México; 2 Universidad Tecnológica de Calakmul, Calakmul, Campeche México; 3 Catedrática CONAHCYT, Colegio de Postgraduados Campus Campeche, Champotón, Campeche México; 4 PFS, Lethbridge, AB Canada

**Keywords:** Apiculture, Beekeeping, Campeche, Floristics, Quintana Roo, Yucatán

## Abstract

**Background:**

The Yucatan Peninsula (YP) is one of the most important regions in global apiculture. Hence, this work reviews and integrates the knowledge of the species diversity, growth habits, ecosystems, floral calendars during the apiculture production cycles and the types of vegetation represented in the melliferous flora (MF) of the YP; as a basis for proposing selection strategies locating suitable apiculture production areas for local beekeepers and help in the economic development of the region.

**Methods:**

A comprehensive review of the MF literature was carried out using the snowball method to determine and update the number of species useful for apiculture. The growth habits and flower calendars were determined through a review of the literature and databases of specimens from the herbaria CICY, UCAM and MEXU.

**Results:**

The YP reports a total of 935 taxa of MF (98 families and 498 genera); of these, Campeche has 812 taxa, followed by Quintana Roo (786) and Yucatán (767). The MF is made up of herbs (282), followed by shrubs (260), trees (229), climbers (82), woody climbers (67) palms (14) and parasitic plant (1).

**Conclusion:**

Of the 935 species of MF registered at the regional level, a high number of species have flowering throughout the year, however, not all of these species are considered useful for local commercial apiculture. Only a select group of 23 species are considered of major importance for local apiculture industry.

**Supplementary Information:**

The online version contains supplementary material available at 10.1186/s13002-024-00681-0.

## Background

Apiculture (beekeeping) in Mexico is one of the main economic agricultural activities with an average production of 57,000 tons of honey per year; placing the country in the sixth place globally [1]. Beekeeping in the country based on the level of technical development and type of honey (both physicochemical and organoleptic characteristics); and is divided into five regions (north, center, highlands, Pacific, Gulf and southeast or Yucatán peninsula (YP) (made up of the states of Campeche, Quintana Roo and Yucatán) [2]. The YP is one of the major apiculture production areas of the region with around 30–35% of the *Apis mellifera* L. bee colonies being located here; and for exporting 80–95% of the local honey to the international market [3].

Honey from the YP is appreciated in the international markets for its organoleptic characteristics (color, aroma and flavor) depending on the specific biotic and abiotic conditions of the region [4]. However, honey production varies depending on where the apiaries are established and the availability of floral resources (nectar and pollen) that the plants supply throughout the year, seasonality (dry, rainy and northern or nortes) and diversity and abundance of melliferous flora (MF) present in each type of vegetation [5]. MF is the set of plant species that produce substances or elements that the bees collect as their food (pollen and nectar) [6].

The YP has a flora of 2329 taxa in 956 genera and 161 families as natives [7, 8]. This diversity of plants favors an enormous MF potential for apiculture in the region reported in different studies at the local, state and regional levels [5]. But they vary in the number of species and accepted names; some even include synonyms as accepted names in the same study, leading to under/over-estimates of the total MF of the region, ranging from 370 species [9] to 849–900 species [4, 10, respectively]. Hence, the annual production of honey depends on the seasonality associated with the diversity and phenology of MF, so its production is divided into harvest from January to May (dry = prolonged dry period of reduced or zero rainfall), postharvest from June to September (rainy = high humidity with rainfall) and pre-harvest from October to December (nortes = period of north winds, with high humidity and rain or non-humid, with low temperature) [2, 5].

Although a wide diversity of MF has been reported in the region, only a select group of plants (33–40 species) are known to beekeepers where honey production is obtained during the harvest cycle [11, 12]. Therefore, it is indicated that 90% of the annual honey production of the harvest cycle comes from the nectar flow of *Viguiera dentata* (Cav.) Spreng. (42%, flowering between December and February) and *Gymnopodium floribundum* Rolfe (48%, flowering between March and May) and the remaining 10%, comes from species of legumes (Fabaceae) and climbers of Sapindaceae and Convolvulaceae [2].

On the other hand, it is mentioned that in the postharvest cycle, there is a shortage of food for bees, due to the limited availability of flowering plants that are found around the apiaries [5]. Coupled with anthropogenic factors that limit the availability of bee food with the loss of MF surrounding the apiaries due to the high rates of deforestation in the YP [13]. Furthermore, the extensive use of pesticides and other agro-chemicals on the standing crops close to apiaries; negatively impacts the MF on which the bees forage killing them in significant numbers.

Therefore, it is necessary to have a solid scientific base on MF in terms of its diversity, growth habits, phenology, seasonality, apiculture cycles and types of vegetation where they grow. This knowledge can contribute significantly to complement the information on the flowering periods, assisting higher production of honey throughout the year; and increase the profits of the local beekeepers. Furthermore, they can contribute in maintaining the balance of the local ecosystems via pollination services provided by the bees through the cross pollination of several forest plants and local crops [14].

The objective of this work has been to integrate the knowledge of the MF in the YP to determine its diversity, the types of vegetation where they grow, their growth habits and floral calendars depending on the seasonality and the beekeeping cycle, as a basis for floral selection strategies and locating suitable high apiculture production areas for local beekeepers for generating better average annual income.

## Methods

### Study area

The YP is located in the southeastern portion of the Mexican Republic, comprising of the states of Campeche, Quintana Roo and Yucatán, with an area of approximately 140,000 km^2^ (~ 7% of the national territory); together with the northern part of Belize (Belize, Corozal and Orange Walk districts) and Guatemala (a large part of the department of Petén) constituting the Yucatán Peninsula Biotic Province [7]. According to the Köppen classification modified by García [15], the YP climate is tropical with warm, humid and semi-arid subvariants, characterized by summer rains. Three seasons are identified: (1) hot and dry season (March–May), (2) rainy season (June–October) and (3) winter storms with occasional short rains “nortes” (November–February) [16]. The average annual temperature in the region ranges between 24 and 28 °C, with two thermal zones (west and east) separated by an isothermal limit of 26 °C that goes from the north (Progreso, Yucatán) to the south of the YP (Calakmul Reserve, Campeche). Most of the area receives precipitation between 1000 and 1200 mm of rain, with a precipitation gradient that goes from a dry zone with < 600 mm (northwest of the YP) to a humid zone with > 1500 mm (south and southeast of the YP) [17, 18]. The vegetation of the YP has been described by various authors [19–21], reporting around 16 types of vegetation and standing out for their coverage are the low deciduous forest (northern portion of the YP), the medium subdeciduous forest (central and northern portion of the YP) and medium semi-evergreen forest (southern portion of the YP).

### Data collection and analysis

The information available on the MF species of YP was obtained from academic search engines and scientific journals following the snowball approach [22] using the following keywords: honey flora, beekeeping and Yucatan peninsula. The approach includes random selection of a reference, including relevant information until completing a number of authors that can be considered the main ones on MF. The cut-off point in the search was established when the paper titles and the authors cited begin to repeat themselves; and the number of new references to the list drops significantly. The information collected include local and regional literature from 1981 to 2022 (mostly scientific reports and reviews, under graduate and post graduate student theses, published floras, floristic lists, production manuals, peer reviewed books and book chapters) without any language restrictions (English and Spanish) obtained from the databases of publisher platforms (Scopus-Elservier, Springer, Taylor & Francis Group, Wiley), digital libraries (https://www.proquest.com, https://www.redalyc.org/) and academic social networks (https://www.academia.edu, https://www.researchgate.net). The studies were selected based on the following combined criteria: (1) The study covers the area of the YP, (2) the selected species are the product of palynological analysis, beekeeper interview records, and field observations of researchers and producers. (3) MF studies that only presented a total number of species without a floristic list were excluded.

For the descriptive analyzes of MF, a database was created in a spreadsheet with information on family, species, type of vegetation, growth habits, distribution origin (native, naturalized, cultivated, endemic) and floral calendars (Additional file 1: Table S1). The updating of the scientific names of the MF (synonyms, accepted names, excluded species) was based on Flora of the Yucatán peninsula [23], following the classification of Angiosperm system Phylogeny Website [24]. Association graphs were created with the Pajek v.5.1.4 program [25] between the seasons of the year and the months of MF phenology in the YP. In order to know the MF species with greater frequency in the studies, the relative citation frequency index (CFI) was used, which does not involve the variable u (use-category) and is obtained by dividing the number of authors who mention the species. This index varies when the species is not included (0) to when all the authors mention the species on all occasions (1) [26]. The index was transformed into a percentage in order to generate four frequency intervals, where the first two intervals were considered as those that include the most important species for beekeeping. Subsequently, maps were created to identify the distribution areas (shaded boxes) of the species of the first interval, this through 5 × 5 km abundance grids using the ArcGis version 10.6.1 program. The distribution data of the species projected on the maps were obtained from information from the databases of the CICY, ECOSUR and MEXU herbaria (acronyms according to Thiers [27]) housed in the database of the REMIB nodes of CONABIO. (https://www.snib.mx/), to later project them onto a layer of vegetation from INEGI [28].

The species flowering calendar was established with the information obtained from the MF studies analyzed. In addition, it was complemented with the information of the herbarium specimens available in the Digital Flora Database of the Yucatan Peninsula of the CICY (Centro de Investigación Científica de Yucatán, A.C.) [23]). The flowering calendar for regional use was captured by months of the year and was divided into three seasons (dry, rains and nortes) that correspond to the production cycle or beekeeping cycle (harvest: January to May, postharvest: June to September and pre-harvest: October–December) [5].

## Results

**Fig. 1:**
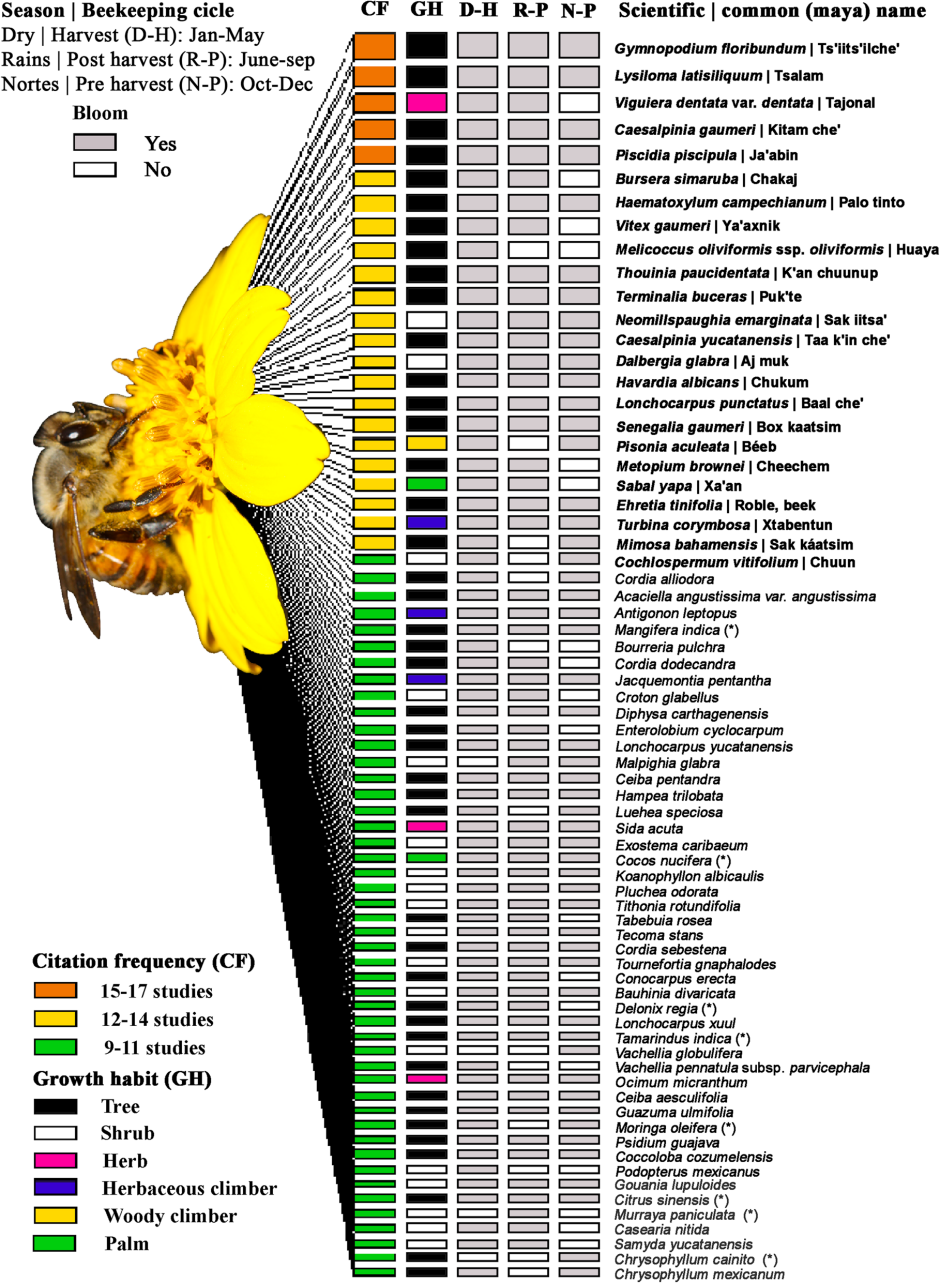
Interaction networks by citation frequency, growth habit and flowering period during the dry, rainy and northern seasons of the main species of melliferous flora in the Yucatan Peninsula, Mexico

**Fig. 2:**
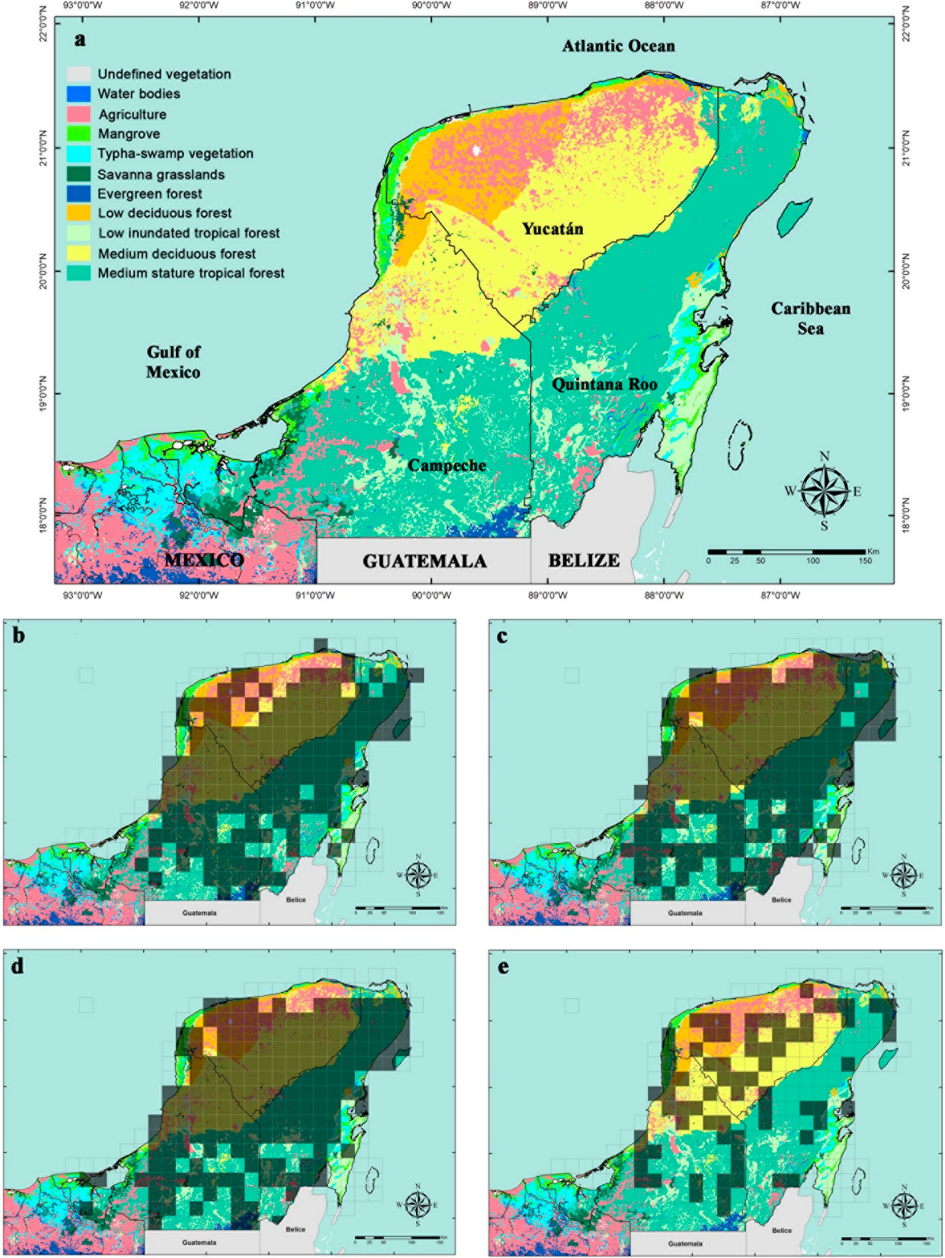
Vegetation types and Cells of 5 × 5 m of the richness of four of the main species of melliferous flora in the Yucatan Peninsula. The shaded boxes indicate areas of distribution of the species. **a** Vegetation types. **b** *Gymnopodium floribundum*. **c** *Lysiloma latisiliquum*. **d** *Piscidia piscipula*. **e** *Viguiera dentata*

**Fig. 3:**
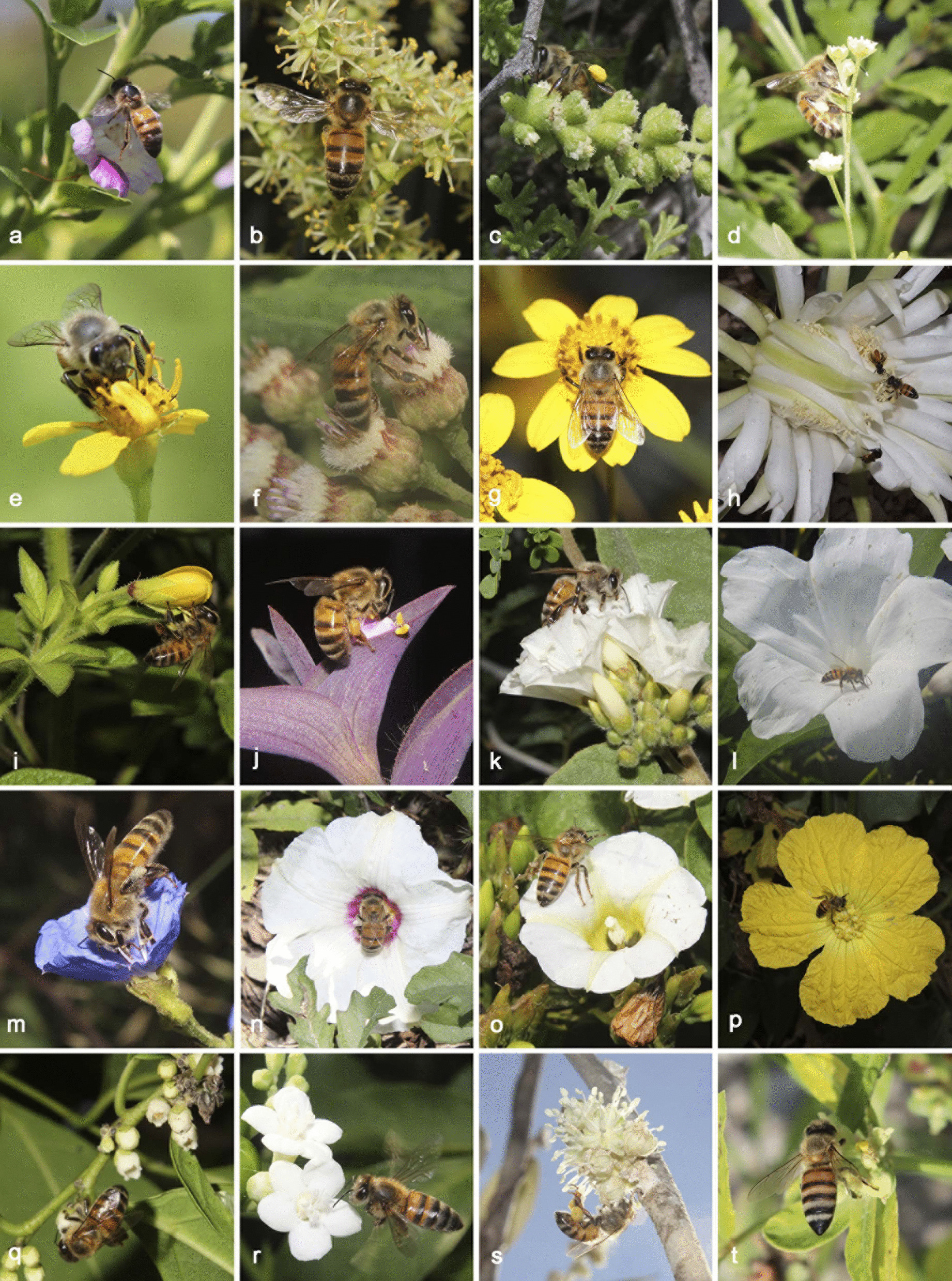
Melliferous flora with *Apis mellifera*. **a** *Bravaisia berlandieriana*, **b** *Sabal mexicana*. **c** *Ambrosia hispida*. **d** *Parthenium hysterophorus*. **e** *Acmella pilosa*. **f** *Pluchea carolinensis*. **g** *Viguiera dentata*. **h** *Acanthocereus tetragonus*. **i** *Corynandra viscosa*. **j** *Tradescantia pallida*. **k** *Convolvulus nodiflorus*. **l** *Ipomoea carnea* ssp. *fistulosa*. **m** *Jacquemontia pentantha*. **n** *Distimake dissectus*. **o** *Turbina corymbosa*. **p** *Luffa aegyptiaca*. **q** *Diospyros yatesiana*. **r** *Cnidoscolus souzae*. **s** *Croton peraeruginosus*. **t** *Euphorbia cyathophora*. Photographs: W. Cetzal-Ix

**Fig. 4:**
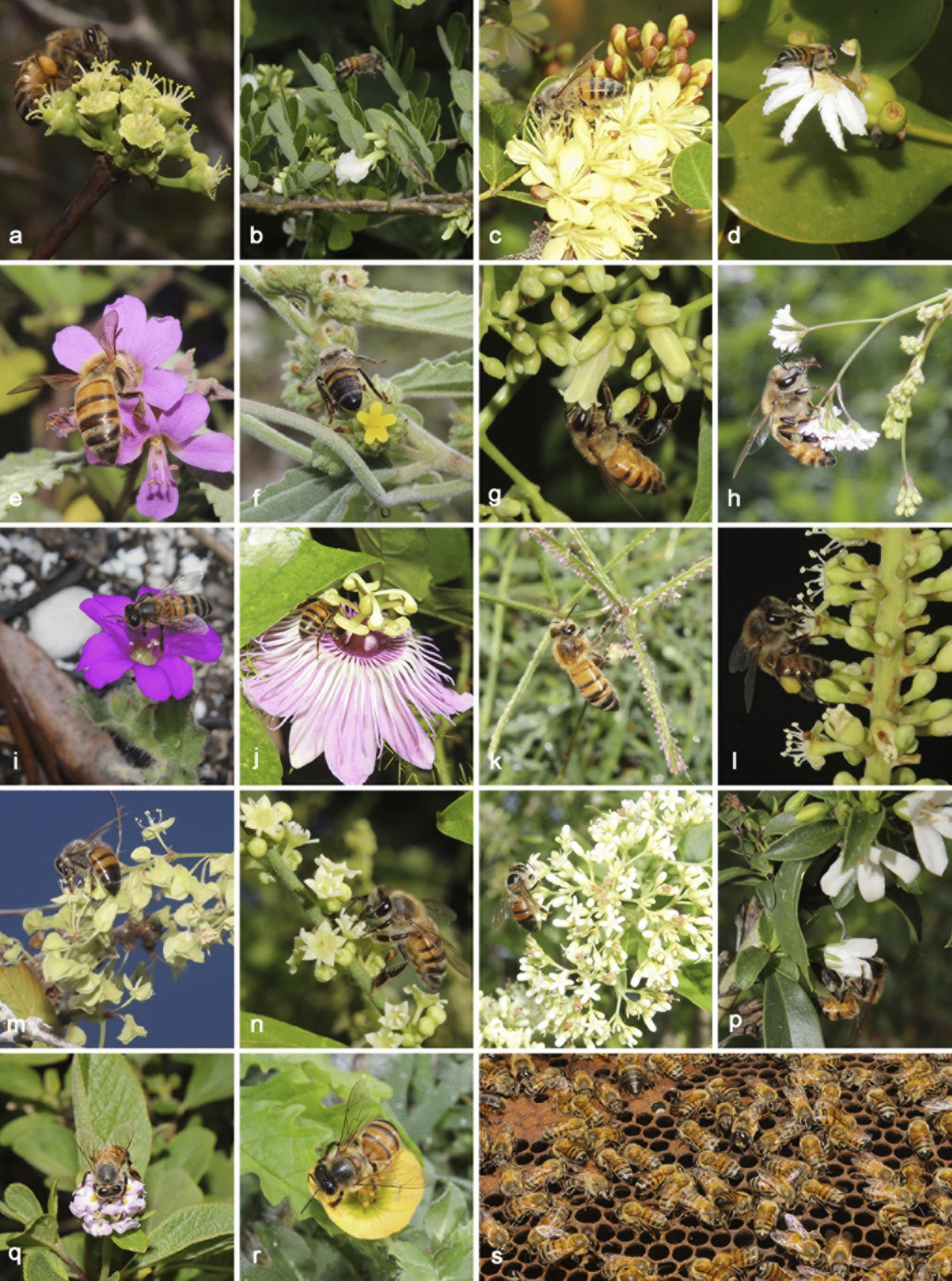
Melliferous flora with *Apis mellifera*. **a** *Euphorbia schlechtendalii*. **b** *Dalbergia glabra*. **c** *Haematoxylum campechianum*. **d** *Scaevola plumieri*. e. *Melochia tomentosa*. **f** *Waltheria americana*. **g** *Cedrela odorata*. **h** *Boerhavia erecta*. **i** *Okenia hypogaea*. **j** *Passiflora foetida*. **k** *Dactyloctenium aegyptium*. **l** *Coccoloba uvifera*. **m** *Gymnopodium floribundum*. **n** *Gouania lupuloides*. **o** *Machaonia lindeniana*. **p** *Capraria Mexicana*. **q** *Lantana involucrata*. **r** *Kallstroemia pubescens*. **s** *Apis mellifera*. Photographs: W. Cetzal-Ix

Out of the of 18 MF studies recorded in the YP between 1981 and 2022 (Table 1); five were based on palynological analysis, five on producer interviews, and seven on field methods or observations. Based on the MF citation frequency of the analyzed studies, five MF species were repeatedly found in 17 research publications (Figs. 1, 2), 18 among 8–9 studies (Fig. 1), 47 were recorded in 15 studies, and 851 species were found between 2–3 studies. Derived from the compilation of these studies, a total of 935 MF taxa were recorded for YP, distributed across 98 families and 498 genera (Additional file 1: Table S1, Figs. 3, 4).

**Table 1:** Comparison of the chronological number of melliferous flora species included by different authors in the Yucatan peninsula

Authors	FA	GE	TA	SP	SSP	VA	SY	ND	TE	MI	AN	C
Souza-Novelo et al. (1981)	78	225	357	294	4	9	108	2	48	29	177	[52]
Suárez-Molina (1981)	34	108	133	113	2	6	41	–	12	7	74	[53]
Chemas y Rico-Gray (1991)	19	32	37	30	1	2	4	–	4	–	29	[54]
Rico-Gray et al. (1991)	30	78	89	82	–	4	14	–	3	–	74	[47]
Villegas-Durán et al. (1998)	41	157	217	183	10	13	45	–	11	12	156	[55]
Ayala-Arcipreste (2001)	21	51	56	45	2	2	12	7	7	–	37	[56]
Arellano et al. (2003)	104	422	928	857	22	32	241	5	18	2	668	[10]
Porter-Bolland (2003)	29	69	101	74	3	4	16	15	8	2	65	[36]
Zamora-Crescencio (2003)	2	7	7	7	–	–	1	–	–	–	6	[57]
Chi-Quej (2009)	21	32	34	32	1	1	5	–	–	–	29	[58]
Zamora-Crescencio et al. (2009)	9	15	16	16	–	–	3	–	1	–	14	[59]
Villanueva-Gutiérrez et al. (2009)	23	32	44	30	1	3	5	7	–	1	29	[35]
Alfaro-Bates et al. (2010)	18	32	33	30	1	2	8	–	1	1	24	[33]
Alfaro-Bates et al. (2011)	17	40	50	43	3	4	5	–	–	–	45	[12]
Carnevali et al. (2010)	25	75	99	85	3	11	14	–	15	2	61	[7]
CONABIO & AECID (2011)	33	70	79	71	8	–	11	–	17	–	63	[41]
Coh-Martínez et al. (2019)	26	50	56	52	3	1	–	–	–	–	56	[5]
Briceño-Santiago et al. (2022)	29	55	64	54	–	–	2	–	–	1	54	[60]
This study	98	498	935	876	18	41	–	–	–	–	–	

FA, Family; GE, Genus; TA, Taxa; SP, Species; SSP, Subspecies; VA, Variety; SY, Synonym; ND, Not determined; TE, Taxa excluded; MI, Misidentification; AN, Accepted name; C, Citation

**Fig. 5:**
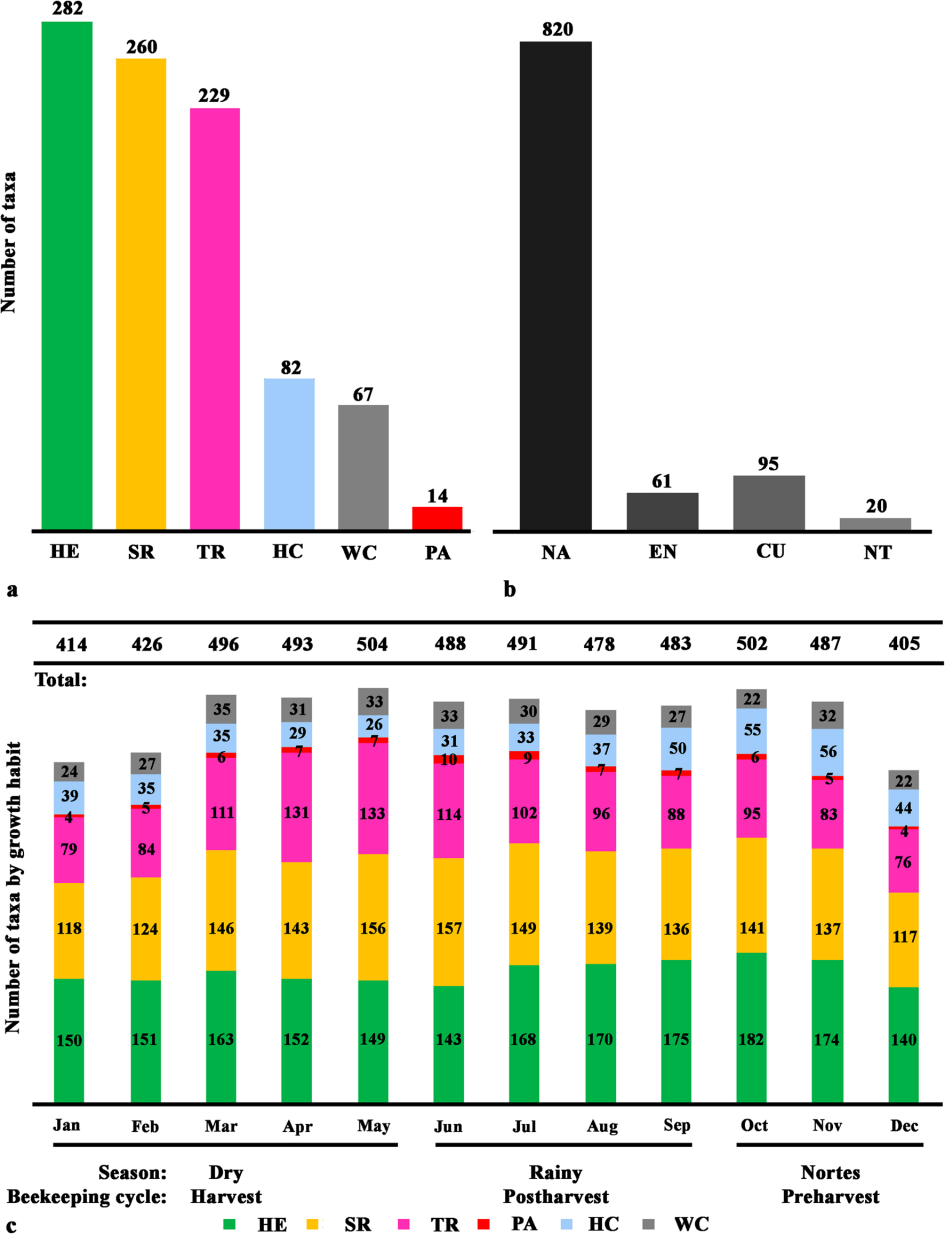
Melliferous flora. **a** Number of species per growth habit. **b** Botanical origin. c. Number of species and growth habit that bloom per month and season

In growth habits, the highest number of species was recorded in herbs (282), shrubs (260) and trees (229) and the lowest in herbaceous climbers (82), woody climbers (67), palms (14) and parasitic plant (1) (Fig. 5a). Regarding their distribution origin, 820 are native (61 endemic), 20 naturalized and 95 cultivated (Fig. 5b). The state in the YP with the highest MF diversity was Campeche with 812 taxa, followed by Quintana Roo (786 taxa) and Yucatán (767 taxa).

During the dry season, there was an increase in flowering species (March to May), with May having the highest number of flowering taxa (504), as well as October (502) when the northern season begins in the YP (Fig. 5c). On the other hand, December and January (405 and 414, respectively) had the lowest number of flowering species (Fig. 5c). The greatest diversity of MF was found in the dry season with 788 taxa, followed by the rainy season with 728 taxa and finally the north with 626 taxa.

A total of 182 herbs showed flowering during the beginning of the nortes (October), while May and June are the ones that showed the highest flowering of shrubby species. On the other hand, the trees showed flowering in the driest season of the year (April–May) with a total of 131 and 133 taxa, respectively (Fig. 5c). Climbing species are more frequent in October and November (55–56 taxa respectively) when honey production decreases in the YP (Fig. 5c).

**Fig. 6:**
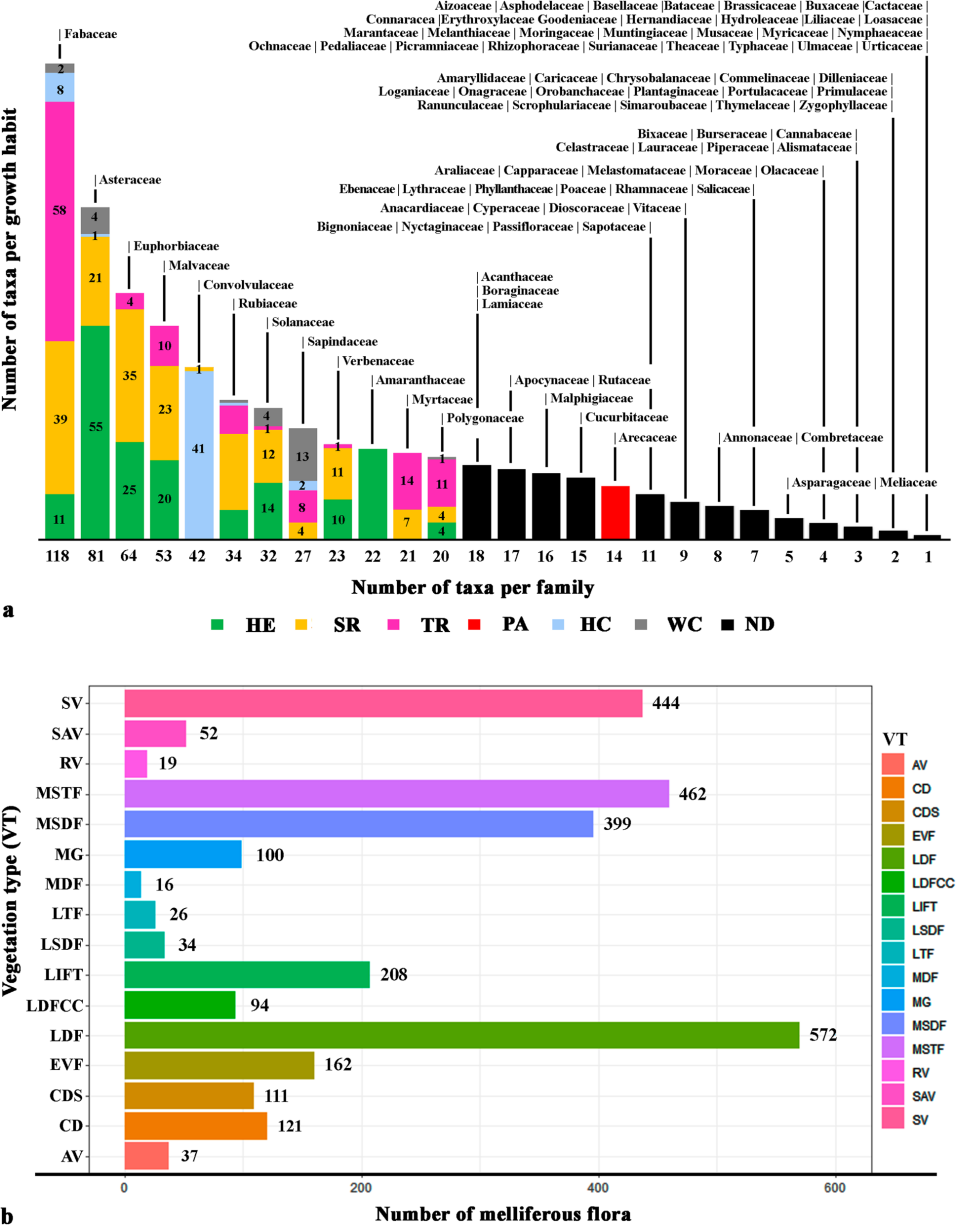
Melliferous flora per family (**a**) and vegetation types (**b**)

The botanical families of MF major represented in this study are Fabaceae, Asteraceae and Euphorbiaceae with 118, 81 and 61 taxa, respectively (Fig. 6a). Regarding MF diversity by vegetation type, the highest number was recorded in the low deciduous forest (LDF) with 572 taxa, followed by the medium subevergreen forest (MSTF) with 462 taxa and secondary vegetation (SF) with 444 taxa. In the growth habits of the MF by vegetation type, the LDF presented the greatest diversity of species of herbs (168), shrubs (165) and climbers (60), while the MSTF presented the greatest diversity of tree species (140), followed by the LDF with 136 species, and the MSTF presented the highest diversity of woody climbing species with 47 (Fig. 6b). The species reported as most common and important for beekeeping (~ 12 to 16 studies) are present in most types of vegetation in the region. This pattern is also observed when species reported in the range of 9–11 studies are included, for example, *Acaciella angustissima* var. *angustissima* (Additional file 2: Fig. S1).

In reporting the unique MF by the type of vegetation; 144 species were recorded. Out of these, 95 are native (3 endemic), 44 cultivated and 5 naturalized. Similarly, by growth habit, 51 herbs, 39 trees, 33 shrubs, 10 lianas, 8 climbers and 3 palms were found. The highest number of unique species was found in the secondary vegetation, followed by LDF and medium stature tropical forest (Additional file 3: Fig. S2).

## Discussion

### Melliferous flora of importance for honey production

A total of 23 species of MF play a very important role for beekeeping in the YP, within these species is *G. floribundum* “ts'iitsilche” (Mayan name), which is considered the main source of nectar production for bees in the central and northern portion of the YP [2, 29]. The honey produced with *G. floribundum* is of high value for beekeepers, since it is in demand in European markets for its quality and flavor [30]. However, Villanueva-Gutiérrez [11] mentions that in addition to *G. floribundum*, there are other highly important honey species for honey production such as *V. dentata* “tajonal” (Spanish name), *Metopium brownei* “chechem” (Mayan name), *Bursera simaruba* “chaká” (Mayan name) and *Thouinia canescens* “kaan-chunub” (Mayan name), which were found among the 16 most important species for honey production. It also agrees with what was reported by SIAP [31], CONABIO [32] and Alfaro-Bates et al. [33], which indicates that the preferred food sources for *A. mellifera* in the YP come from *B. simaruba*, *G. floribundum*, *L. latisiliquum*, *P. piscipula*, *Turbina corymbosa* “xtabentún” (Mayan name) and *V. dentata*.

The previous MF species considered as the main food sources were confirmed by Villanueva-Gutiérrez [34], through load samples (palynological analysis) in European and African bees in apiaries established in the Quintana Roo. Likewise, Villanueva-Gutiérrez et al. [35] using similar method confirmed that *B. simaruba*, *P. piscipula* and *V. dentata* are priorities for honey production in the YP, agreeing with the information obtained through surveys to producers by Porter-Bolland [36] and Zapata-Cauich [37].

Currently, various authors have continued to conduct interviews with producers in the YP, for example, Aguilar-Hernández et al. [38] found *B. simaruba*, *P. piscipula* and *V. dentata* as the most important nectar-pollinator plants during the harvest season in Quintana Roo. Coh-Martínez et al. [5] recorded 56 MF species using the same method; indicating that beekeepers only depend on 18 species (includes the aforementioned) to obtain their honey production in the Xmaben community in Campeche. This agrees when the MF studies of the region are analyzed together, with 15–40 species considered to be of greater importance for beekeeping.

However, MF species reported to be of high importance for honey production vary in the number of flowers available per year, seasonality, quality and quantity of nectar generated, and its abundance in different types of vegetation [39, 40]. Therefore, it is necessary to evaluate these aspects different scales of the landscape in the YP, as well as the pollen grains in honey from apiaries to identify their presence and abundance; flowering periods and distribution patterns to determine their local utility.

### Growth habit

In the growth habits of MF at the regional level, a greater number of herbs were found, followed by shrubs and trees; however, the beekeepers in the interviews of the different local studies indicate that the trees are of greater importance for the production of honey, which is reflected in the different studies that are based on surveys. For example, Carnevali et al. [7] include 99 MF species, of these, 42 correspond to trees and 36 to herbs, followed by shrubs (11), lianas (6) and herbaceous climbers (4). While CONABIO & AECID [41] records 93 species, of these, 36 are trees, 17 shrubs, followed by herbs (13), herbaceous climbers (5), woody climbers (2) and palms (2). This same pattern is also observed in local studies of the predominance of tree species, Porter-Bolland [36] of 101 MF species recorded in the mountainous area of Campeche, a total of 83 are trees and 10 shrubs, followed by climbing species (5) and vines (3). Likewise, Coh-Martínez et al. [5] reported for this same area, a greater diversity of trees (39), followed by shrubs (6), herbs (5), climbers (4) and lianas (2).

The botanical families of MF best represented in this study are Fabaceae, Asteraceae and Euphorbiaceae; these families also stand out within the five families with the highest number of species at the national and regional level, Asteraceae (3,057, 147), Fabaceae (1,903, 230), Orchidaceae (1,213, 132), Poaceae (1,047, 216) and Euphorbiaceae with (714, 113) [7, 8].

### Melliferous flora by seasons

The lowest number of flowering MF species was recorded from December to February (in the transition from nortes to dry season), although in general the three seasons recorded a similar number of MF species (Fig. 5). However, the most important species for beekeeping in the YP were recorded in the dry season, when trees in dry forests lose their leaves from 50 to 75% and in humid forests from 25% as a strategy to resist seasonal drought [7, 32, 42]. During the dry season from March to April (harvest period in beekeeping), the highest flowering peak of plants occurs (mainly legume species) for their subsequent fruiting throughout the rainy season [43]. The flowering pattern of increase or decrease in MF during the climatic seasons or for the beekeeping cycle cannot be easily observed at a regional scale, but when it is evaluated at a local scale, the flowering of MF increases in the dry seasons and decreases with the rain season [5]. This observed pattern is possibly due to the fact that the studies include only MF species of high importance for the beekeeping cycle derived from the knowledge of the interviewed producers. Considering that the honeys obtained during the dry season are of higher quality because they present a lower amount of moisture (greater than or equal to 20%) [12, 44], which contributes to reducing the presence of microorganisms that cause honey fermentation [45].

The rainy season in the YP is considered by beekeepers as a time of scarcity of food for bees due to the limited availability of floral resources [40]. A similar diversity of MF was recorded here with respect to other stations, but this flora is considered by beekeepers as of little nutritional importance for bees; hence they tend to incorporate food supplements to prevent the escape of bees from the hive [42]. In addition, beekeepers indicate that the honey produced in the rainy season is of poor quality due to the high humidity that allows easy fermentation of honey, reduction in storage time and changes in its organoleptic properties [32, 42]. At the beginning of this season (June), the most important species for beekeeping are still in bloom; but they decrease in July and August (Fig. 5), then the availability of herbaceous species increases, allowing the maintenance of bee hives.

Regarding the nortes season, a similar high number of MF flowering species were also found in October and November; but with a decrease in December. In beekeeping, this season is considered the recovery of bee colonies due to the increase in the availability of nectar food sources [2]. In this season, the most important species for bees are climbing herbaceous plants of the Convolvulaceae family that can grow in disturbed sites or with open spaces or roadsides where their establishment is favored by the availability of light in the different types of vegetation in the region [5, 42].

### Species distribution

The distribution of the most important species for apiculture has been recorded in the northern and central portions of the YP in the low and medium deciduous and subdeciduous forests [7]. For example, *V. dentata* grows mainly in low deciduous forest, low forest with columnar cacti, low floodplain forest, semideciduous medium forest, and in secondary vegetation and coastal dunes. For their part, *G. floribundum* and *L. latisiliquum* also grow in these same types of predominant vegetation throughout the north and center of the region [23]. The collections in the southern zone of these species are scarce, due to the fact that they dominate more humid ecosystems such as MSFT and low flooded forests [46].

The state with the highest number of MF species at the regional level is Campeche, followed by Quintana Roo and Yucatán, influencing Campeche to be the largest honey-producing area, in addition to the fact that it has a tradition inherited from pre-Hispanic times of caring for bees and its existing MF [14, 47]. Hence, the rural communities of Campeche have been able to take advantage of this wide diversity of MF of the dry and humid regions for the production of honey [40, 48]. Furthermore, it is important to mention that organic honey is exported from the municipality of Calakmul in Campeche, with humid forests; since about half of its territory is located within the Calakmul Biosphere Reserve, an extensive protected area where one can take advantage of the beekeeping as an economic alternative that does not affect the ecosystem [42]. However, in terms of productivity, Yucatan is the main producer and exporter of honey at the national level [31]. In 2015, out of 15,058 tons of honey produced in Mexico; Yucatán contributed 45%, Campeche 37% and Quintana Roo 18% [31].

The honey obtained in the YP, according to its MF composition, is classified as monofloral (with a dominant type of pollen > 45%) and multifloral (with several types of pollen < 45%) [33]. Some authors indicate that 58% of the honey produced in Yucatan is monofloral (obtained from *Viguiera dentata*, *Mimosa bahamensis* and *Bursera simaruba*) and multifloral in Campeche (with 10 types of honey) and Quintana Roo (with five types of honey) [49]. Alfaro-Bates et al. [33] indicate that these differences in the types of honey are due to the diversity of MF that Campeche and Quintana Roo have with respect to Yucatan, since bees have more MF options available for their food. However, in the dry and humid forests of the YP (in a gradient from south to north) these floristic elements considered as monofloral or of the majority of the MF species of high importance for honey production, share the different types of vegetation and are distributed homogeneously throughout the region (Figs. 1 and 2). Even so, the populations of these species differ in their flowering periods and flowering peaks depending on where they grow, being probably monofloral according to this classification based on the type of vegetation, peak and favorable year of flowering when the honey is obtained (pre-harvest, harvest and postharvest) for melissopalynological analysis. In the YP, monofloral honeys possibly come from apiaries established in core or transition areas of mangroves in coastal areas with particular and dominant species such as *Avicennia germinans* (Additional file 2: Fig. S1); this is when their harvest is carried out in the rainy season and when the flowering of the plants decreases most important species for beekeeping (Laynes-Magaña et al., in prep.).

### Apiculture challenges

The following challenges are directly related to the scarcity or lack of floral food (nectar and pollen) impacting the local bee colonies, making them more susceptible to attacks by various pathogens. Among the challenges facing apiculture in the region are diseases such as varroosis (*Varroa destructor* Anderson & Trueman), pest infestations by *Aethina tumida* Murray as well as rapid deforestation and habitat fragmentation of areas where the apiaries are located [5, 13]. Furthermore, the availability of bee food during the rainy season when the number of species in bloom decreases; causing a dip in the breeding percentages in the bee colony or the bees abandon their nests/hives in search of nectar and pollen. Hence, the beekeepers have to provide food supplements in the form of sugar syrup for the subsistence of the bees [2]. This decline in local bee populations for floral resources has been analyzed in European and Africanized bees during October in the Sian Ka'an Reserve in Quintana Roo, Mexico by Ceballos-Martínez [50] and Villanueva-Gutiérrez et al. [51].

## Conclusion

Of the 925 species of MF registered at the regional level, a high number of species have flowering throughout the year; however, not all of these species are considered useful for local commercial apiculture. The quality and quantity of nectar varies considerably between the different species. Furthermore, the availability of flora varies with respect to the distribution, abundance, quantity and quality of the nectar; as well as the amount of pollen generated. The integrated MF species are important for bee feeding and foraging can be selected from different reforestation and habitat restoration programs of the sites where apiaries are located. This will promote increased honey production as well as help in successful conservation of the local ecosystem. Finally, the flowering periods of the 23 local species important for beekeeping can serve as the basis for selection, establishment and enrichment of floral assemblages around the local apiaries. Such an approach, will successfully provide year long food (nectars and pollen) for the bees throughout the honey production cycle. This strategy can help avoid the unfortunate periods of bee starvation making them weak and susceptible to various diseases and pathogens; and prevent them from swarming away from the existing bee colonies for better foraging and feeding alternatives available to the bees.

### Supplementary Information

**Additional file 1**.** Table S1**: Checklist of melliferous flora of Yucatan peninsula, Mexico. SVN = Spanish vernacular name. MVN = Mayan vernacular name. Growth habit (GH): HE = Herb, PA = Palm, SH = Shrub, TR = Tree, HC = Herbaceous climber, WC = Woody climber. Vegetation types (VT): CD = Coastal dune, CDS = Coastal dune scrub, MG = Mangrove, SAV = Savanna grasslands, EVF = Evergreen forest, LTF = Low tropical forest, LDF = Low deciduous forest, LDFCC = Low deciduous forest with columnar cacti, LIFT = Low inundated tropical forest, LSDF = Low subdeciduous forest, MDF = Medium deciduous forest, MSDF = Medium subdeciduous forest, MSTF = Medium stature tropical forest, AV = Aquatic vegetation, SV = Secondary vegetation, RV = Riparian vegetation. Origin distribution (OD): NA = Native, NT = Naturalized, CU = Cultivated, EN = Endemic. Distribution per state in Yucatan peninsula (DYP): CA = Campeche, QR = Quintana Roo, YU = Yucatán. Months of year (J, F, M, A, M, J, J, S, O, N, D).

**Additional file 2**. **Figure S1**: Network of interaction between the types of vegetation and most important species for beekeeping in the Yucatan peninsula. Number below the boxes represent the numbers of the images to indicate their scientific name.

**Additional file 3**. **Figure S2**: Interaction networks between vegetation types and species recorded as unique to these ecosystems (including growth habit, botanical origin, endemism and flowering period with respect to the climatic season and beekeeping cycle). NT = Number of taxa by type of vegetation.

## Data Availability

All data are available in this paper.

## Abbreviations

CFI: Citation frequency index
LDF: Low deciduous forest
MF: Melliferous flora
MSTF: Medium subevergreen forest
SF: Secondary vegetation
YP: Yucatán peninsula
