# Supplementary material for: A review of the melliferous flora of Yucatan peninsula, Mexico, on the basis for the honey production cycle
Source: J Ethnobiol Ethnomed. 2024 Mar 25;20:40. doi: 10.1186/s13002-024-00681-0 (PMC10964660; doi:10.1186/s13002-024-00681-0)
Supplement: Supplementary file 1 — Additional file 1. Table S1: Checklist of melliferous flora of Yucatan peninsula, Mexico. SVN = Spanish vernacular name. MVN = Mayan vernacular name. Growth habit (GH): HE = Herb, PA = Palm, SH = Shrub, TR = Tree, HC = Herbaceous climber, WC = Woody climber. Vegetation types (VT): CD = Coastal dune, CDS = Coastal dune scrub, MG = Mangrove, SAV = Savanna grasslands, EVF = Evergreen forest, LTF = Low tropical forest, LDF = Low deciduous forest, LDFCC = Low deciduous forest with columnar cacti, LIFT = Low inundated tropical forest, LSDF = Low subdeciduous forest, MDF = Medium deciduous forest, MSDF = Medium subdeciduous forest, MSTF = Medium stature tropical forest, AV = Aquatic vegetation, SV = Secondary vegetation, RV = Riparian vegetation. Origin distribution (OD): NA = Native, NT = Naturalized, CU = Cultivated, EN = Endemic. Distribution per state in Yucatan peninsula (DYP): CA = Campeche, QR = Quintana Roo, YU = Yucatán. Months of year (J, F, M, A, M, J, J, S, O, N, D). [file 13002_2024_681_MOESM1_ESM.docx]

**Supplementary material Table S1** Checklist of melliferous flora of Yucatan peninsula, Mexico. SVN = Spanish vernacular name. MVN = Mayan vernacular name. Growth habit (GH): HE = Herb, PA = Palm, SH = Shrub, TR = Tree, HC = Herbaceous climber, WC = Woody climber. Vegetation types (VT): CD = Coastal dune, CDS = Coastal dune scrub, MG = Mangrove, SAV = Savanna grasslands, EVF = Evergreen forest, LTF = Low tropical forest, LDF = Low deciduous forest, LDFCC = Low deciduous forest with columnar cacti, LIFT = Low inundated tropical forest, LSDF = Low subdeciduous forest, MDF = Medium deciduous forest, MSDF = Medium subdeciduous forest, MSTF = Medium stature tropical forest, AV = Aquatic vegetation, SV = Secondary vegetation, RV = Riparian vegetation. Origin distribution (OD): NA = Native, NT = Naturalized, CU = Cultivated, EN = Endemic. Distribution per state in Yucatan peninsula (DYP): CA = Campeche, QR = Quintana Roo, YU = Yucatán. Months of year (J, F, M, A, M, J, J, S, O, N, D).

| **Family** | **Taxa** | **SVN** | **MVN** | **GH** | **VT** | **OD** | **DYP** | **J** | **F** | **M** | **A** | **M** | **J** | **J** | **A** | **S** | **O** | **N** | **D** |
| --- | --- | --- | --- | --- | --- | --- | --- | --- | --- | --- | --- | --- | --- | --- | --- | --- | --- | --- | --- |
| Acanthaceae | *Aphelandra scabra* (Vahl) Sm. | Cola de gallo | Chak anal | HE | LDF, LDFCC, LTF, MSDF, SV | NA | CA, QR, YU | + | + | + | + | + | + | + | + | + | + | + | + |
| Acanthaceae | *Bravaisia berlandieriana* (Nees) T. F. Daniel | - | Juluub | SH | AV, CD, CDS, MG, LDF, LDFCC, LIFT | NA | CA, QR, YU | + | + | + | + | + |  | + |  |  |  | + |  |
| Acanthaceae | *Dicliptera sexangularis* (L.) Juss. | - | K’u wech | HE | CDS, LDF, MSDF, MSTF, SV | NA | CA, QR, YU | + | + | + | + |  | + |  |  |  | + | + |  |
| Acanthaceae | *Elytraria bromoides* Oerst. | Lengua de sapo | Chjiolu’um | HE | LDF, MSDF | NA | CA, QR, YU |  |  |  |  |  | + | + |  |  |  |  |  |
| Acanthaceae | *Elytraria imbricata* (Vahl) Pers. | Papachillo | Kaba xaan | HE | LDF, LDFCC, MSDF, SV | NA | CA, QR, YU | + | + | + | + | + |  | + | + | + | + | + | + |
| Acanthaceae | *Henrya insularis* Nees ex Benth. |  | Aka’ xiiw | HE | LDF, MSDF | NA | YU | + | + | + | + |  |  |  |  |  |  |  |  |
| Acanthaceae | *Justicia brandegeeana* Wassh. & L. B. Sm. | - | - | HE | LDF, SV | CU | QR |  |  |  |  |  |  |  |  |  | + |  | + |
| Acanthaceae | *Justicia breviflora* (Nees) Rusby | - | - | HE | LDF | NA | CA, QR | + | + | + | + | + |  |  | + |  |  |  |  |
| Acanthaceae | *Justicia campechiana* Standl. ex Lundell | Damiana | - | SH | LIFT, MSDF | NA | CA, QR, YU | + |  | + |  | + |  |  |  |  |  | + |  |
| Acanthaceae | *Justicia carthagenensis* Jacq. | Cruz k’aax | Took’ sits’ | HE | CD, CDS, LDF, LDFCC, LIFT, MG, MSDF, SV | NA | CA, QR, YU | + | + | + | + |  | + | + | + | + | + | + | + |
| Acanthaceae | *Justicia lundellii* Leonard. | - | Aka’ xiiw | HE | LDF, LDFCC, MSDF, SV | NA | CA, QR, YU | + | + | + |  |  |  | + | + | + | + | + | + |
| Acanthaceae | *Odontonema callistachyum* (Schltdl. & Cham.) Kuntze | Canutillo | - | HE | LDF, RV | CU | YU |  | + |  |  | + |  |  |  | + |  | + |  |
| Acanthaceae | *Odontonema tubaeforme* (Bertol.) Kuntze | Pavón rojo | - | SH | EVF, SV | NA | CA, QR, YU | + | + | + | + | + | + | + |  |  |  |  |  |
| Acanthaceae | *Ruellia blechum* L. | Sornia | Aka’xiiw | HE | CDS, MG, MSDF, MSTF, SV | NA | CA, QR, YU | + | + | + | + | + |  |  | + | + | + | + | + |
| Acanthaceae | *Ruellia inundata* Kunth | Me’ex chivo | Chak mul | HE | MSDF, MSTF, LDF, SV | NA | CA, QR, YU | + | + | + |  |  |  |  |  |  | + | + | + |
| Acanthaceae | *Ruellia nudiflora* (Engelm. & A. Gray) Urb. | Berraco xiiw | Che’su’uk | HE | CD, LDF, MSDF, MSTF, SV | NA | CA, QR, YU | + |  | + | + | + | + | + | + | + | + | + | + |
| Acanthaceae | *Ruellia paniculata* L. | - | - | HE | LDF, LIFT, MG, MSTF | NA | CA, YU | + | + | + | + | + |  |  |  |  | + | + | + |
| Acanthaceae | *Ruellia pereducta* Standl. ex Lundell | - | - | HE | MSTF, EVF | NA | CA, QR, YU |  | + | + | + | + | + |  |  |  |  |  |  |
| Acanthaceae | *Tetramerium nervosum* Nees | Corrimiento xiiw | Aka’ xiiw | HE | LDF, MSTF | NA | CA, QR, YU | + | + | + | + | + |  |  | + | + | + | + | + |
| Aizoaceae | *Sesuvium portulacastrum* (L.) L. | Verdolaga de playa | Ts’a’aykann | HE | AV, CD, CDS, MG | NA | CA, QR, YU |  |  | + |  | + | + | + | + | + | + | + | + |
| Alismastaceae | *Echinodorus berteroi* (Spreng.) Fassett | Planta de celofán | - | HE | AV, LDF, SV | NA | YU | + | + | + |  |  |  |  | + | + | + | + | + |
| Alismastaceae | *Echinodorus subalatus* (Mart.) Griseb. | Flor de agua | Kibix | HE | AV, LDF, SV | NA | CA, QR, YU | + | + | + | + | + |  | + | + | + | + | + | + |
| Alismataceae | *Sagittaria lancifolia* L. subsp. *media* (Micheli) Bogin | Lirio | - | HE | AV, MSTF, SV | NA | CA | + | + | + | + | + | + | + | + | + | + | + | + |
| Amaranthaceae | *Achyranthes aspera* L. | Zorrillo | Payche’ | HE | LDF, SV | NT | CA, QR, YU | + |  | + |  |  | + |  |  | + |  | + | + |
| Amaranthaceae | *Alternanthera halimifolia* (Lam.) Standl. | Diamante | Sak payche | HE | CD | NA | YU |  | + |  |  |  |  |  |  |  |  |  |  |
| Amaranthaceae | *Alternanthera lanceolata* Schinz | - | Zakmuul | HE | MSTF, RV | NA | CA |  |  |  |  |  |  | + | + | + | + | + |  |
| Amaranthaceae | *Alternanthera ramosissima* Chodat | - | Zakmuul | HE | LDF, SV | NA | YU |  |  |  |  | + | + | + | + | + | + | + | + |
| Amaranthaceae | *Alternanthera tenella* Colla | - | - | HE | SV | NA | CA, QR, YU | + |  | + |  |  |  |  |  |  |  |  | + |
| Amaranthaceae | *Amaranthus dubius* Mart. ex Thell. | Verdolaga de costa | Tees ma’ax | HE | LIFT, MSDF | NA | CA, QR, YU | + | + | + | + |  |  |  |  | + | + | + | + |
| Amaranthaceae | *Amaranthus greggii* S. Watson. | - | Xtez | HE | CD, CDS, MG | NA | CA, QR, YU |  |  |  |  |  |  | + | + | + |  |  |  |
| Amaranthaceae | *Amaranthus hybridus* L. | Amaranto | - | HE | LDF, SV | NA | CA, QR, YU | + |  |  |  | + |  |  |  |  |  |  | + |
| Amaranthaceae | *Amaranthus spinosus* L. | - | Tees, k’iix tees | HE | MSDF | NA | CA, QR, YU | + | + | + | + | + | + | + | + | + | + | + | + |
| Amaranthaceae | *Celosia argentea* L. var. *margaritacea* | Cresta de gallo | - | HE | LDF, SV | CU | YU | + | + | + | + | + | + | + | + | + | + | + | + |
| Amaranthaceae | *Celosia chiapensis* Brandegee | Chia | Sabak-pox | HE | MSTF, SV | CU | YU |  |  |  | + |  |  |  |  |  | + | + |  |
| Amaranthaceae | *Celosia nitida* Vahl. | Mano de león | Halalnal | HE | LDF | NA | CA, QR, YU | + |  |  |  |  |  |  |  |  |  |  |  |
| Amaranthaceae | *Celosia virgata* Jacq. | - | Sak payche’ | HE | LDF, SV | NA | CA, QR, YU |  | + | + |  |  |  |  |  |  |  |  |  |
| Amaranthaceae | *Chamissoa altissima* (Jacq.) Kunth var. *altissima* | Epazote | Lukumxiu | HE | SV | NA | QR, YU | + | + | + |  |  |  |  |  |  |  | + | + |
| Amaranthaceae | *Chenopodium ambrosioides* L. | Apazote | - | HE | CD, LDF | NT | CA, QR, YU | + |  |  | + | + | + | + | + |  |  | + |  |
| Amaranthaceae | *Gomphrena filaginoides* M. Martens & Galeotti. | - | Sak mul | HE | LDF, SV | NA | CA, YU |  |  |  |  |  |  |  |  | + | + | + | + |
| Amaranthaceae | *Gomphrena globosa* L. | Amaranto | - | HE | MSDF | NA | YU |  |  | + |  | + |  |  |  |  | + | + | + |
| Amaranthaceae | *Gomphrena serrata* L. | Amor seco | - | HE | LIFT, LDF, MSTF | NA | CA, YU |  |  |  |  |  | + | + | + | + | + | + | + |
| Amaranthaceae | *Iresine diffusa* Humb. & Bonpl. ex Willd. | - | - | HE | CD, CDS, SV | NA | CA, QR, YU | + |  | + |  |  | + |  |  |  |  | + |  |
| Amaranthaceae | *Salicornia bigelovii* Toor. | - | - | HE | CD, MG | NA | CA, QR, YU |  | + | + | + | + |  | + | + | + |  | + |  |
| Amaranthaceae | *Sarcocornia perennis* (Mill.) A. J. Scott | Sosa de las salinas | - | HE | CD | NA | QR, YU |  |  |  |  |  |  |  |  |  | + |  |  |
| Amaranthaceae | *Suaeda mexicana* (Standl.) Standl. | - | - | HE | CD, MG | NA | YU |  | + |  |  |  |  | + |  | + |  |  |  |
| Amaryllidaceae | *Crinum americanum* L. | Lirio | - | HE | AV, MG | CU | CA, QR, YU |  |  | + |  |  | + |  |  |  |  |  |  |
| Amaryllidaceae | *Zephyranthes carinata* Herb. | - | - | HE | SV | CU | CA, QR, YU |  |  |  |  | + | + | + |  |  |  |  |  |
| Anacardiaceae | *Anacardium occidentale* L. | Marañón | - | TR | LTF, SV | CU | CA, YU |  |  | + | + | + |  |  |  |  |  |  | + |
| Anacardiaceae | *Astronium graveolens* Jacq. | Amargoso | K’ulensiis | TR | LDF | NA | CA, QR, YU |  |  |  | + | + |  |  |  |  |  |  |  |
| Anacardiaceae | *Mangifera indica* L. | Mango | - | TR | SV | CU | CA, QR, YU |  | + | + |  | + | + |  | + |  |  | + | + |
| Anacardiaceae | *Metopium brownei* (Jacq.) Urb. | Chechén negro | Cheechem | TR | CD, EVF, LDF, MSDF, MSTF | NA | CA, QR, YU |  |  | + | + | + |  | + | + |  |  |  |  |
| Anacardiaceae | *Mosquitoxylum jamaicense* Krug & Urban | - | - | TR | LTF, MSTF | NA | CA, QR |  |  |  |  |  | + | + | + |  | + |  |  |
| Anacardiaceae | *Spondias mombin* L. | Jobo | Kan abal | TR | LSDF, MSDF | CU | CA, QR, YU |  |  |  |  |  |  | + |  |  |  |  |  |
| Anacardiaceae | *Spondias purpurea* L. | Ciruela | Xkinin | TR | LDF | CU | CA, QR, YU |  | + |  | + | + |  |  |  | + |  |  |  |
| Anacardiaceae | *Spondias radlkoferi* Donn. Sm. | Jobo | - | TR | MSTF, SV | NA | CA |  |  |  | + | + | + |  |  |  |  |  |  |
| Anacardiaceae | *Toxicodendron radicans* (L.) Kuntze | Hiedra venenosa | - | TR | LDF, SV | CU | CA, QR, YU |  |  | + | + | + | + |  |  | + | + | + |  |
| Annonaceae | *Annona cherimola* Mill. | - | - | TR | LDF, SAV | CU | YU |  |  | + | + | + |  | + | + | + |  |  |  |
| Annonaceae | *Annona diversifolia* Saff. | Ilama | - | TR | EVF, LDF, SAV | CU | YU |  |  |  |  |  | + | + | + | + | + |  |  |
| Annonaceae | *Annona glabra* L. | Corcho | - | TR | EVF, LDF, LIFT, MSDF | NA | CA, QR, YU |  |  |  | + | + |  | + |  |  | + |  |  |
| Annonaceae | *Annona muricata* L. | Guanabana | Tokob | TR | LDF, SAV | CU | CA, QR, YU |  |  |  |  |  | + | + | + |  |  |  |  |
| Annonaceae | *Annona primigenia* Standl. & Steyerm. | Anonillo | - | TR | EVF | NA | CA, QR |  |  |  | + |  | + |  |  |  |  |  |  |
| Annonaceae | *Annona purpurea* Moc. & Sessé ex Dunal | Ahate | - | TR | LDF, SAV | CU | YU |  |  |  | + | + | + |  | + | + | + | + | + |
| Annonaceae | *Annona reticulata* L. | Anona dulce | K’an op | TR | LDF, SAV | CU | CA, QR, YU |  |  |  | + |  | + | + |  | + | + |  |  |
| Annonaceae | *Annona squamosa* L. | Saramuyo | Ts’almuy | TR | LDF, SAV | CU | CA, QR |  |  |  |  | + | + |  |  | + |  | + |  |
| Apocynaceae | *Asclepias curassavica* L. | Cancerina | Pool kuuts’ | HE | CDS, EVF, LDF, LDFCC, LIFT, MSDF, MSTF | NA | CA, QR, YU | + | + | + | + | + | + | + | + | + | + | + | + |
| Apocynaceae | *Aspidosperma megalocarpon* Müll. Arg. | - | - | TR | EVF, MSDF | NA | CA |  |  |  | + |  |  | + | + |  |  |  |  |
| Apocynaceae | *Cascabela gaumeri* (Hemsl.) Lippold | Campanilla | Akits | SH | CDS, LDF | NA | CA, QR, YU |  | + | + | + | + |  | + | + | + |  | + | + |
| Apocynaceae | *Cameraria latifolia* L. | Cheechen blanco. | Sak Cheechen | TR | LDF, LIFT, MG, MSDF | NA | CA, QR, YU |  |  | + | + | + | + | + | + | + |  | + | + |
| Apocynaceae | *Dictyanthus aeneus* Woodson | - | - | HC | LDF, LDFCC, LIFT, MG | NA | CA, YU |  |  |  |  |  | + | + | + | + | + | + | + |
| Apocynaceae | *Dictyanthus yucatanensis* Standl. | - | - | HC | LDF, LIFT, LDFCC, MG, MSDF, MSTF | NA | CA, QR, YU |  |  |  |  |  | + | + | + | + | + | + |  |
| Apocynaceae | *Funastrum bilobum* (Hook. & Arn.) J. F. Macbr. | Cormollote | Cawayat | HE | EVF, LDF, MSDF | NA | CA, QR, YU |  |  |  |  |  |  |  |  |  | + | + | + |
| Apocynaceae | *Funastrum clausum* (Jacq.) Schltr. | Isipó de leche | Ya’ax aak’ | HE | LSDF, SV | NA | CA, YU |  |  |  |  |  |  |  |  |  | + | + | + |
| Apocynaceae | *Macroscepis diademata* (Ker Gawl.) W.D. Stevens | - | Aak’tóom paap | HC | EVF, LDF, MSDF | NA | CA, QR, YU |  |  |  |  |  | + | + | + |  |  |  |  |
| Apocynaceae | *Matelea crassifolia* Woodson | - | Mejen k’ek’en | HC | LDF, MSTF | NA | YU | + |  |  |  | + | + | + | + | + | + |  |  |
| Apocynaceae | *Matelea gentlei* Lundell & Standl. | - | - | HC | LDF, MSDF, MSTF | NA | CA, QR, YU |  | + | + |  |  | + | + | + | + |  |  | + |
| Apocynaceae | *Nerium oleander* L. | Adelfa | - | SH | LDF, SV | CU | CA, QR, YU |  | + |  |  |  |  |  |  |  |  |  |  |
| Apocynaceae | *Oxypetalum cordifolium* (Vent.) Schltr. | - | - | HE | SV | CU | CA, QR |  |  |  |  |  |  |  |  |  |  | + | + |
| Apocynaceae | *Plumeria obtusa* L. | Flor de mayo | Aak’its | TR | LDF, LDFCC, LTF, MSDF, SV | NA | CA, QR, YU |  |  |  |  | + | + |  |  |  |  |  |  |
| Apocynaceae | *Plumeria rubra* L. | Flor de mayo | Sak nikté | TR | LDF, SV | NA | CA |  |  |  | + | + | + |  | + |  |  |  |  |
| Apocynaceae | *Tabernaemontana amygdalifolia* Jacq. | Cojón de caballo | Uts’um péek’ | SH | LDF, MSDF | NA | CA, QR, YU |  | + |  | + | + |  |  | + | + |  |  |  |
| Apocynaceae | *Tabernaemontana coronaria* (Jacq.) Willd. | - | - | SH | MSDF, SV | CU | CA, QR, YU |  |  |  |  | + |  |  |  |  |  | + |  |
| Apocynaceae | *Thevetia ahouai* (L.) A. DC. | Cojón de venado | - | SH | EVF, MDF, MSDF, MSTF, SV | NA | CA, QR, YU |  |  | + | + | + | + | + | + | + | + |  | + |
| Araliaceae | *Dendropanax arboreus* (L.) Decne. & Planch. | - | Sak chakaj | TR | EVF, LDF, MSDF, MSTF | NA | CA | + | + | + | + | + | + | + | + | + | + | + | + |
| Araliaceae | *Hydrocotyle bonariensis* Lam. | Corona de santa | - | HE | LIFT, RV | NA | CA, QR, YU | + | + |  | + |  | + | + |  |  | + | + | + |
| Araliaceae | *Hydrocotyle umbellata* L. | Ombligo de venus | - | HE | LIFT, RV | NA | CA |  |  | + | + | + | + | + | + |  |  |  |  |
| Araliaceae | *Hydrocotyle verticillata* Thunb | Lochita | - | HE | AV, LIFT | NA | CA | + | + | + | + |  |  |  | + |  |  | + |  |
| Arecaceae | *Acoelorraphe wrightii* (Griseb. & H. Wendl.) H. Wendl. ex Becc. | Guano prieto | - | PA | EVF, MG | NA | CA, QR, YU |  | + | + | + | + |  |  |  |  |  |  |  |
| Arecaceae | *Acrocomia aculeata* (Jacq.) Lodd. ex Mart. | Cocoyol | Tuk’ | PA | MSTF, SAV, SV | NA | CA, QR, YU |  |  |  |  |  | + | + | + |  |  |  |  |
| Arecaceae | *Attalea cohune* Mart. | Corozo | - | PA | EVF, LTF, MSTF | NA | CA |  | + |  |  |  | + |  |  |  |  |  |  |
| Arecaceae | *Chamaedorea seifrizii* Burret | - | Xiat | PA | CDS, LDF, MSDF, MSTF | NA | CA, QR, YU |  | + | + | + | + | + | + |  |  |  |  |  |
| Arecaceae | *Cocos nucifera* L. | Cocotero | - | PA | CD, SV, RV | CU | CA, QR, YU | + | + | + | + | + | + | + | + | + | + | + | + |
| Arecaceae | *Cryosophila stauracantha* (Heynh.) R. Evans | Escoba | - | PA | EVF, MSDF | NA | CA, QR, |  |  |  |  |  |  |  |  | + | + | + |  |
| Arecaceae | Desmoncus *orthacanthos* Mart. | - | Bayal | PA | EVF, MSTF | NA | CA, QR |  |  | + | + | + | + | + |  |  |  |  |  |
| Arecaceae | *Gaussia maya* (Cook) H. Quero et R. W. Read | - | - | PA | MSTF | NA, EN | CA, QR |  |  | + | + | + | + | + | + | + |  |  |  |
| Arecaceae | *Pseudophoenix sargentii* H. Wendl. ex Sarg. | Palma de guinea | Kuka', ya'ax jalalche' | PA | CDS, LDF, MSDF | NA | QR, YU |  |  |  |  |  | + | + | + | + | + |  |  |
| Arecaceae | *Roystonea dunlapiana* P. H. Allen | Palma real | - | PA | LIFT, SV | NA | CA, QR | + |  |  |  |  | + | + | + | + | + | + | + |
| Arecaceae | *Roystonea regia* (Kunth) O. F. Cook. | Palma real | - | PA | LIFT, SV | NA | CA, QR |  |  |  | + | + | + | + | + | + | + | + | + |
| Arecaceae | *Sabal mexicana Mart.* | Guano bon | Bon xa’an | PA | CDS, LDF, MSDF, SV | NA | CA, YU | + | + | + | + |  |  |  |  |  |  |  |  |
| Arecaceae | *Sabal yapa C.* Wright. ex Becc. | Guano macho | Julok' xa'an, xa'an | PA | CDS, EVF, MSTF, LDF, LIFT, LDFCC | NA | CA, QR, YU |  |  |  |  | + | + | + |  |  |  |  |  |
| Arecaceae | *Thrinax radiata* Lodd. ex Schult. & Schult. f. | - | Ch'íit | PA | CD, CDS, MG | NA | CA, QR, YU | + | + | + | + | + | + | + | + | + | + | + | + |
| Asparagaceae | *Agave angustifolia* Haw. var. *angustifolia* | Henequén de playa | Kij, ch’elem | HE | CDS, LDF, LDFCC, LIFT, MSDF, SV | NA | CA, QR, YU | + | + | + | + |  |  |  |  | + | + | + | + |
| Asparagaceae | *Agave fourcroydes* Lem. | Henequén. | Ki | HE | LDF, SV | CU | YU |  |  | + | + | + |  |  |  |  |  |  |  |
| Asparagaceae | *Agave sisalana* Perrine ex Engelm. | Sisal | Ya’ax ki | HE | SV | CU | CA, QR, YU |  |  |  | + |  |  |  |  |  |  |  | + |
| Asparagaceae | *Dracaena marginata* hort. | Dracena | - | HE | SV | CU | CA, QR, YU | + |  |  |  | + |  |  |  |  |  |  |  |
| Asparagaceae | *Echeandia luteola* Cruden | - | - | HE | LDF, LIFT, SV | NA, EN | CA, QR, YU |  |  |  |  |  |  |  |  | + | + |  |  |
| Asphodelaceae | *Aloe vera* (L.) Burm. f. | Sábila | - | HE | SV | CU | CA, QR, YU | + |  |  |  |  |  |  |  |  |  |  |  |
| Asteraceae | *Acmella oppositifolia* (Lam.) R. K. Jansen var. *oppositifolia* | Lagunea | K'utumbuy | HE | MSDF, SV | NA | CA, QR, YU | + |  |  |  |  |  | + | + | + | + | + | + |
| Asteraceae | *Ageratum conyzoides* L. | Pasmo xiiw | - | HE | MSDF, MSTF, SV | NA | QR | + | + | + | + | + | + | + | + | + | + | + | + |
| Asteraceae | *Ageratum corymbosum* Zuccagni ex Pers. | - | - | HE | MSDF, SV | NA | QR, YU |  |  |  |  |  |  | + | + | + | + | + | + |
| Asteraceae | *Ageratum houstonianum* Mill. | Agerato | - | HE | LDF, MSDF, MSTF, SV | NA | CA, QR, YU | + |  |  |  |  |  |  |  | + | + | + | + |
| Asteraceae | *Ageratum maritimum* Kunth | - | Hauay-ché | HE | CD, LIFT, MCD, MG, SV | NA | CA, QR, YU | + | + | + | + | + | + |  |  |  | + | + | + |
| Asteraceae | *Ambrosia hispida* Pursh | Altanisa de mar | Muuch’ kook | HE | CD, MCD, MG, LIFT | NA | CA, QR, YU |  |  | + | + | + | + | + | + | + | + |  |  |
| Asteraceae | *Ambrosia peruviana* Willd. | Artemisa | Apazote xiiw | HE | CD, LDF, MSDF, MSTF, SV | NA | CA, QR |  |  | + | + | + | + | + | + | + | + |  |  |
| Asteraceae | *Artemisia vulgaris* L. | Ajenjo altamisa | Tsi’tsi’n | HE | SV | CU | CA, QR, YU |  |  |  |  |  | + | + | + |  |  |  |  |
| Asteraceae | *Aster subulatus* Michx. var. *subulatus* | Lechuga de monte | - | HE | SV | NA | CA, QR |  |  |  |  |  |  |  | + | + | + | + |  |
| Asteraceae | *Baccharis dioica* Vahl | - | - | SH | CD, MCD | NA | QR, YU |  |  | + | + | + | + | + | + | + | + | + |  |
| Asteraceae | *Baltimora recta* L. | Limoncillo | Xk'aanam | HE | LDF, SV | NA | CA, QR, YU | + | + | + | + |  |  |  | + | + | + | + | + |
| Asteraceae | *Bidens cynapiifolia* Kunth | - | Matsa ch’ich  bu’ul | HE | MSDF, MSTF | NA | CA, YU |  |  |  |  |  |  | + | + | + | + | + |  |
| Asteraceae | *Bidens reptans* (L.) G. Don | - | - | HC | MSDF, MSTF, SV | NA | CA, QR, YU | + | + | + | + | + | + | + | + | + | + | + | + |
| Asteraceae | *Bidens riparia* Kunth var. *riparia* | - | Ch'ik bu'ul | HE | SV | NA | CA |  |  |  |  |  |  |  |  | + | + | + |  |
| Asteraceae | *Bidens pilosa* L. | Té de milpa | K’an tumbuub | HE | LDF, MSTF, MSDF, SV | NA | CA, QR, YU | + | + | + | + | + | + | + | + | + | + | + | + |
| Asteraceae | *Borrichia arborescens* (L.) CD. | Margarita de mar | K’an lool xiiw | SH | CD, MCD | NA | CA, QR, YU |  |  | + | + | + | + | + | + | + | + | + | + |
| Asteraceae | *Borrichia frutescens* (L.) CD. | - | Tsooj | HE | CD, MCD | NA | CA, QR, YU |  |  | + | + | + | + | + | + |  |  |  |  |
| Asteraceae | *Brickellia diffusa* (Vahl) A. Gray | - | - | HE | LDF, MSTF | NA | CA, QR | + | + |  |  |  |  |  |  |  |  |  | + |
| Asteraceae | *Calea jamaicensis* (L.) L. | Malvavisco silvestre | tu’ xikin | SH | LDF, LDFCC, MCD, MSDF, MSTF | NA | YU |  |  |  |  |  |  | + | + | + | + | + | + |
| Asteraceae | *Calea megacephala* B. L. Rob. & Greenm. | - | - | HE | LDF, SAV | CU | YU |  |  |  |  |  |  |  | + |  |  |  |  |
| Asteraceae | *Calea urticifolia* (Mill.) DC. var. *yucatanensis* Wussow, Urbatsch & G. A. Sullivan | - | Xikin, tu’ xikin | HE | CDS, LDF, LDFCC | NA | QR, YU | + | + | + | + | + | + | + | + | + | + | + | + |
| Asteraceae | *Calea urticifolia* (Mill.) CD. var. *urticifolia* | - | Xikin | SH | CDS, LDF, LDFCC | NA | CA, QR, YU | + | + | + | + | + | + | + | + | + | + | + | + |
| Asteraceae | *Chromolaena lundellii* R. M. King & H. Rob. | - | - | SH | LDF, MSTF, MSDF | NA | CA, QR, YU | + | + | + |  |  |  |  |  |  | + | + | + |
| Asteraceae | *Chromolaena odorata* (L.) R. M. King & H. Rob. | - | Tok’abam | HE | CDS, LDF, LDFCC, LIFT, MSDF, MSTF, SV | NA | CA, QR | + | + | + |  |  |  |  |  |  | + | + | + |
| Asteraceae | *Cirsium horridulum* Michx. | Cardo | Oomil | HE | MSDF, MSTF | NA | CA, QR, YU |  |  | + | + | + | + | + | + | + | + | + | + |
| Asteraceae | *Conyza bonariensis* (L.) Cronquist | Apazote de monte | - | HE | SV | NA | CA, QR, YU | + | + | + | + | + | + | + | + | + | + | + | + |
| Asteraceae | *Conyza canadensis* (L.) Cronquist | Lechuga | Soi kay | HE | SV | NA | CA, QR, YU | + | + | + | + | + | + | + | + | + | + | + | + |
| Asteraceae | *Cosmos sulphureus* Cav. | - | - | HE | SV | NT | CA, QR, YU | + | + | + | + | + | + | + | + | + | + | + | + |
| Asteraceae | *Critonia campechensis* (B. L. Rob.) R. M. King & H. Rob. | Corrimiento aak’ | Sak cáncer | SH | MSDF, MSTF, SV | NA, EN | CA, QR, YU | + | + | + |  |  |  |  | + | + | + | + | + |
| Asteraceae | *Critonia daleoides* CD. | Tok’kabal | - | SH | MSDF, MSTF, SV | NA | CA, QR | + | + | + | + |  |  |  |  |  |  |  | + |
| Asteraceae | *Critonia morifolia* (Mill.) R. M. King & H. Rob. | Anis xiiw | K’an lool xiiw | SH | LDF, LIFT, MSDF, MSTF, SV | NA | QR | + | + | + | + | + | + |  |  |  |  |  |  |
| Asteraceae | *Critoniopsis oolepis* (S. F. Blake) H. Rob. | - | Op’tsiimin | HE | LDF | NA, EN | CA, QR, YU | + | + | + | + |  |  |  |  |  |  |  |  |
| Asteraceae | *Delilia biflora* (L.) Kuntze | - | Soi kay | HE | MSDF, MSTF, SV, LDF, LDFCC, LIFT | NA | CA, QR, YU | + |  |  |  |  |  |  | + | + | + | + | + |
| Asteraceae | *Eclipta prostrata* (L.) L. | Zarzaparrilla de tago | - | HE | LIFT, LDF, SV | NA | CA, QR | + | + | + | + | + | + | + | + | + | + | + | + |
| Asteraceae | *Egletes liebmannii* Sch. Bip ex Klatt | - | - | HE | AV, LIFT | NA | CA, QR | + | + | + | + | + | + | + | + |  |  |  |  |
| Asteraceae | *Emilia fosbergii* Nicolson | Pincelillo | - | HE | LDF, MSDF, MSTF, SV | NT | CA, QR | + | + | + | + | + | + | + | + | + | + | + | + |
| Asteraceae | *Eremosis oolepis* (SF Blake) Gleason | Axihuitl | Sunchín | HE | LDF, LTF | NA, EN | CA, QR | + | + | + | + |  |  |  |  |  |  |  |  |
| Asteraceae | *Flaveria linearis* Lag. | Girasol | - | HE | CD, CDS, LDF, LIFT, LDFCC, MG, MSDF, MSTF, SV | NA | CA, QR | + | + | + | + | + | + | + | + | + | + | + | + |
| Asteraceae | *Flaveria trinervia* (Spreng.) C. Mohr | - | k’an lool xiiw | HE | CD, CDS, LDF, LIFT, LDFCC, MG, MSDF, MSTF, SV | NA | CA, QR |  |  | + | + | + | + | + | + | + | + | + | + |
| Asteraceae | *Fleischmannia microstemon* (Cass.) R. M. King & H. Rob. | - | - | HE | LDF, MSDF, SV | NA | QR, YU | + | + | + | + |  |  |  |  |  | + | + | + |
| Asteraceae | *Fleischmannia pycnocephala* (Less.) R.M. King & H. Rob. | - | - | HE | CDS, LDF, LDFCC, LIFT, MSDF, MSTF, SV | NA | CA, QR, YU | + | + | + | + | + | + | + | + |  |  |  | + |
| Asteraceae | *Goldmanella sarmentosa* (Greenm.) Greenm. | Tajonal | - | HE | EVF, MSDF, MSTF | NA | CA, QR, YU | + | + | + | + | + | + | + | + |  |  | + | + |
| Asteraceae | *Harleya oxylepis* (Benth.) S. F. Blake | Tajonal | K’antun boob” | HE | LDF, MSDF, SV | NA | QR |  |  | + | + |  |  |  |  |  |  |  |  |
| Asteraceae | *Hebeclinium macrophyllum* (L.) CD. | Corcho | Arepa xiw | HE | MSDF, MSTF | NA | CA, QR | + | + | + | + | + | + | + | + | + | + | + | + |
| Asteraceae | *Helianthus annuus* L. | Girasol | Hon-tolok | HE | SV | CU | CA, QR, YU |  |  | + | + | + | + | + |  |  |  |  |  |
| Asteraceae | *Koanophyllon albicaule* (Sch. Bip. ex Klatt) R. M. King & H. Rob. | Margarita | Teresita ka’ax | SH | CDS, LDF, LDFCC, MSDF, MSTF, SV | NA | CA, QR, YU | + | + | + | + | + | + | + | + | + | + | + | + |
| Asteraceae | *Lagascea mollis* Cav. | Tajonal | k’antun boob | HE | CDS, LDF, LDFCC, LIFT, MSDF, MSTF, SV | NA | CA, YU | + |  |  |  |  | + | + | + | + | + | + | + |
| Asteraceae | *Lasianthaea fruticosa* (L.) K. M. Becker var. *fruticosa* | - | Tank’as aak’ | SH | CDS, LDF, LDFCC, LIFT, MSDF, MSTF, SV | NA | CA, QR, YU | + | + | + | + | + | + | + | + | + | + | + | + |
| Asteraceae | *Melampodium divaricatum* (Rich.) CD. | - | - | HE | SV | NA | CA, QR, YU | + | + | + | + | + | + | + | + | + | + | + | + |
| Asteraceae | *Melampodium gracile* Less. | Bejuco raspador | - | HE | LDF, SV | NA | CA, YU | + | + | + | + | + | + | + | + | + | + | + | + |
| Asteraceae | *Melanthera nivea* (L.) Small | Levisa xiiw | sooj | HE | CDS, LDF, LDFCC, MSDF, MSTF, SV | NA | CA, QR, YU | + | + | + | + | + | + | + | + | + | + | + | + |
| Asteraceae | *Milleria quinqueflora* L. | Árbol maravilla falso girasol and árnica de la tierra | - | HE | CDS, LDF, LDFCC, MSDF, MSTF, SV | NA | CA, QR, YU | + | + |  |  |  |  |  | + | + | + | + | + |
| Asteraceae | *Montanoa atriplicifolia* (Pers.) Sch. Bip. | Margarita | jom tolok | SH | CDS, LDF, LDFCC, MSDF, MSTF, SV | NA | CA, QR, YU | + | + | + | + | + | + |  |  | + | + | + | + |
| Asteraceae | *Neurolaena lobata* (L.) Cass. | - | ya’ax chulkeej | SH | CDS, LDF, LDFCC, MSDF, MSTF, SV | NA | CA, QR, YU | + | + | + | + | + | + | + | + | + | + | + | + |
| Asteraceae | *Otopappus curviflorus* (R. Br.) Hemsl. | Hierba de san juan | Xiiw in | WC | LDF, MSDF, MSTF | NA | CA, QR, YU | + | + | + |  |  |  |  |  |  |  | + | + |
| Asteraceae | *Otopappus guatemalensis* (Urb.) R.L. Hartman & Stuessy | Incienso aak | sak taj | WC | LDF, MSDF, MSTF | NA, EN | CA, QR, YU | + | + | + | + | + | + |  |  |  |  | + | + |
| Asteraceae | *Otopappus scaber* S. F. Blake | Bejuco raspador | - | WC | LDF, MSDF, MSTF | NA | CA, QR, YU | + | + |  |  |  |  |  |  | + | + | + | + |
| Asteraceae | *Parthenium hysterophorus* L. | - | - | HE | CD, MSDF, SV | NA | CA, QR, YU | + | + | + | + | + | + | + | + | + | + | + | + |
| Asteraceae | *Pluchea carolinensis* (Jacq.) G. Don | Tuete | - | SH | AV, CD, CDS, LDF, LDFCC, LIFT, MSDF, MSTF, SV | NA | CA, QR, YU |  | + | + | + | + | + |  |  |  |  |  |  |
| Asteraceae | *Pluchea odorata* (L.) Cass. | Tajonal | Sak xo’ xiiw | HE | CDS, LDF, LIFT, MSDF | NA | CA, QR, YU | + | + | + | + | + | + | + | + | + | + | + | + |
| Asteraceae | *Porophyllum punctatum* (Mill.) S. F. Blake | - | Uk’iil | SH | CD, LDF, LDFCC, LIFT, SV | NA | CA, QR, YU | + | + | + | + | + | + | + | + | + | + | + | + |
| Asteraceae | *Porophyllum ruderale* var. *macrocephalum* (CD.) Cronquist | Papaloquelite | P’eech’ uk’iil | HE | LDF, LDFCC, LIFT, SV | NA | CA, QR, YU | + | + | + |  |  |  | + | + | + | + | + | + |
| Asteraceae | *Pseudelephantopus spicatus* (B. Juss. ex Aubl.) C. F. Baker | - | - | HE | LDF, SV | NA | CA, YU | + | + | + | + | + | + | + | + | + | + | + | + |
| Asteraceae | *Pseudoconyza viscosa* (Mill.) D'Arcy | - | - | HE | CD, CDS, LDF, MG, SV | NA | CA, QR, YU |  |  | + | + | + |  |  |  |  |  |  |  |
| Asteraceae | *Pseudogynoxys chenopodioides* (Kunth) | Estrellita del cielo | Kusam | WC | CD, CDS, LDF, LIFT, MG, MSDF, MSTF, SV | NA | CA, QR, YU | + | + | + | + | + | + | + | + | + | + | + | + |
| Asteraceae | *Sanvitalia procumbens* Lam. | Ojo de gallo | k’antoom buub | HE | LDF, MSDF, MSTF | NA | CA, QR, YU | + | + | + | + | + | + | + | + | + | + | + | + |
| Asteraceae | *Sclerocarpus divaricatus* (Benth.) Benth. & Hook. f. ex Hemsl. | Tajonal | k’antoom boob | HE | LDF, MSDF, MSTF | NA | CA, QR, YU | + | + | + | + | + | + | + | + | + | + | + | + |
| Asteraceae | *Sphagneticola trilobata* (L.) Pruski | Margarita rastrera | k’utumbuy | HE | CD, CDS, LDF, MG, SV | NA | CA, QR, YU |  |  | + | + | + |  |  |  |  |  |  |  |
| Asteraceae | *Synedrella nodiflora* (L.) Gaertn. | Cerbatana | - | HE | MSDF, MSTF | NA | CA, QR, YU |  |  |  |  |  |  | + | + | + | + | + | + |
| Asteraceae | *Tagetes erecta* L. | Flor de muerto | Pujuk | HE | CD, CDS, LDF, LIFT, LDFCC, MSDF, MSTF, SV | NT | CA, QR, YU | + | + | + | + | + | + | + | + | + | + | + |  |
| Asteraceae | *Tagetes lucida* Cav. | Pericón | - | SH | SAV | NA | CA |  |  |  |  |  |  | + | + | + | + | + |  |
| Asteraceae | *Tithonia diversifolia* (Hemsl.) A. Gray | Árnica | Su'um | SH | CD, CDS, LDF, LDFCC, LIFT, MSDF, MSTF, SV | NT | CA, QR, YU | + | + | + | + | + |  |  | + | + | + | + | + |
| Asteraceae | *Tithonia rotundifolia* (Mill.) S. F. Blake | Árnica | Su'um | SH | CD, CDS, LDF, LDFCC, LIFT, MSDF, MSTF, SV | NT | CA, QR, YU | + | + | + | + | + | + | + | + | + | + | + | + |
| Asteraceae | *Tithonia tubaeformis* (Jacq.) Cass. | - | - | SH | CD, CDS, LDF, LDFCC, LIFT, MSDF, MSTF, SV | NA | CA | + | + | + | + | + | + | + | + | + | + | + | + |
| Asteraceae | *Tridax procumbens* L. | Pasmado xiiw | Ta'ulu'um | HE | CD, CDS, LDF, LDFCC, LIFT, MSDF, MSTF, SV | NA | CA, QR, YU | + | + | + | + | + | + | + | + | + | + |  |  |
| Asteraceae | *Trixis inula* Crantz | Corrimiento | Ya'ax k'an aak' | SH | CD, CDS, LDF, LDFCC, LIFT, MSDF, MSTF, SV | NA | CA, QR, YU | + | + | + | + | + |  |  | + | + | + | + | + |
| Asteraceae | *Verbesina gigantea* Jacq. | Árnica | Chul keej | HE | LDF, LDFCC, LIFT, MSDF, MSTF, SV | NA | CA, QR, YU | + | + | + | + |  |  |  |  | + | + | + | + |
| Asteraceae | *Verbesina myriocephala* Sch. Bip. ex Klatt | - | - | SH | MSDF, MSTF, LIFT | NA | QR | + |  |  |  |  |  |  |  | + | + | + | + |
| Asteraceae | *Vernonanthura patens* (Kunth) H. Rob. | - | - | SH | LDF, MSTF | NA | CA | + | + | + |  |  |  |  |  | + | + | + | + |
| Asteraceae | *Viguiera dentata* (Cav.) Spreng. var. *dentata* | Tajonal | Sak xo’ xiiw | HE | CD, CDS, LDF, LDFCC, LIFT, MSDF, MSTF, SV | NA | CA, QR, YU | + | + | + | + | + | + | + | + | + | + | + | + |
| Asteraceae | *Wedelia acapulcensis* Kunth | - | - | HE | MSDF, MSTF | NA | CA, QR, YU | + | + | + | + | + | + | + | + | + | + | + | + |
| Asteraceae | *Wedelia acapulcensis* Kunth var. *ramosissima* (Greenm.) Strother | - | - | HE | MSDF, MSTF | NA | CA, QR, YU | + | + | + | + | + | + | + | + | + | + | + | + |
| Basellaceae | *Anredera vesicaria* (Lam.) C.F. Gaertn. | - | - | HC | LDF, MSTF | NA | CA, QR, YU |  |  |  |  |  |  |  |  | + | + | + |  |
| Bataceae | *Batis maritima* L. | Alambrillo | Ts'aay kaan | SH | CDS, LDF, MG, SV | NA | CA, QR, YU |  |  | + |  | + | + | + |  |  |  |  |  |
| Bignoniaceae | *Arrabidaea floribunda* (Kunth) Loes. | - | Anilkab | WC | MSTF, MDF, MSDF | NA | CA, QR, YU | + |  | + | + | + | + | + | + | + | + | + |  |
| Bignoniaceae | *Crescentia cujete* L. | Jicara | Waas | TR | AV, CDS, LDF, LSDF, LIFT, SAV, SV | NA | CA, QR, YU |  | + | + | + | + | + | + | + |  | + | + |  |
| Bignoniaceae | *Cydista diversifolia* (Kunth) Miers | - | - | WC | LDF, MSDF, MSTF, SV | NA | CA, QR, YU |  | + | + |  | + | + | + | + | + | + | + | + |
| Bignoniaceae | *Cydista potosina* (K. Schum. & Loes.) Loes. | - | - | WC | LDF, MSTF, SV | NA | CA, QR, YU | + |  | + | + | + | + | + | + | + |  | + | + |
| Bignoniaceae | *Handroanthus chrysanthus* (Jacq.) S.O. Grose | Mauche’ | K'ank lool | WC | MSDF, MSTF, SV | NA | CA, QR, YU |  |  | + | + |  |  |  |  |  |  | + |  |
| Bignoniaceae | *Parmentiera aculeata* (Kunth) Seem. | Pepino  de árbol | Kat | SH | MSDF, MSTF | NA | CA, QR, YU |  |  | + | + | + | + | + | + | + | + | + | + |
| Bignoniaceae | *Tabebuia rosea* (Bertol.) DC. | Roble | Hok’Ab | TR | LIFT, LDF, MSDF, MSTF, SV | NA | CA, QR, YU | + | + | + | + | + | + |  |  | + |  |  |  |
| Bignoniaceae | *Tecoma stans* (L.) Juss. ex Kunth | Tronador | Xk´anlol | SH | CDS, LDF, LIFT, MG, MSDF, MSTF | CU | CA, QR, YU | + | + | + | + | + |  |  |  | + | + | + | + |
| Bignoniaceae | *Cydista aequinoctialis* (L.) Miers | - | - | WC | AV | CU | YU |  |  |  | + | + | + |  |  |  |  |  |  |
| Bignoniaceae | *Parmentiera millspaughiana* L. O. Williams | Pepino de monte | Kat ku’uk | TR | LDF, LIFT, MSDF, MSTF, SV | NA | CA, YU |  | + | + | + | + |  | + | + | + | + | + | + |
| Bignoniaceae | *Stizophyllum riparium* (Kunth) Sandwith | Frijolillo | Sak bay éek | WC | LDF, MSDF, MSTF, SV | NA | CA, QR, YU |  |  | + | + | + | + |  | + | + |  |  |  |
| Bixaceaceae | *Amoreuxia wrightii* A. Gray | - | Kabal chuun | HE | LDF | NA | CA, QR, YU |  |  |  | + |  | + |  | + |  |  |  |  |
| Bixaceaceae | *Cochlospermum vitifolium* (Willd.) Spreng. | Madera de  pasta | Chuun | SH | LDF, LDFCC, LIFT, MSTF, SV | NA | CA, QR, YU | + | + | + | + |  |  |  |  |  | + |  | + |
| Bixaceae | *Bixa orellana* L. | Achiote | K'uxub | SH | LDF, SV | CU | CA, QR, YU | + | + |  |  |  |  | + |  | + | + | + |  |
| Boraginaceae | *Bourreria mollis* Standl. | Roble | Sak bay éek | TR | EVF, LDF, LSDF, LTF, MSTF, MSDF | NA | CA, QR | + |  |  |  |  | + | + | + | + | + | + | + |
| Boraginaceae | *Bourreria pulchra* (Millsp.) Greenm. | - | Sak boj | TR | LDF, LTF, LIFT, MDF, MSDF, MSTF, SV | NA, EN | CA, QR, YU | + | + | + |  |  |  |  |  |  |  |  |  |
| Boraginaceae | *Cordia alliodora* (Ruiz & Pav.) Oken | Bojón prieto | Bojum | TR | LDF, MSDF, MSTF | NA | CA | + | + | + |  | + |  |  |  |  | + |  |  |
| Boraginaceae | *Cordia dodecandra* L*.* | Siricote | Chak k’oopte’eor | TR | LDF, MSDF, MSTF, SV | NA | CA, QR, YU |  | + | + | + | + | + |  |  |  |  |  |  |
| Boraginaceae | *Cordia gerascanthus* L. | - | Bojom | TR | LDF, LSDF, LIFT, MDF, MSTF, MSDF, SV | NA | CA, QR, YU | + | + | + | + | + |  |  |  |  |  |  |  |
| Boraginaceae | *Cordia sebestena* L. | Anacahuite, | k’oopte’ | TR | CD, CDS, LSDF, MSTF, SV | NA | CA, QR, YU |  |  | + | + | + | + | + | + | + | + | + |  |
| Boraginaceae | *Ehretia tinifolia* L. | Roble | Beek | TR | EVF, LDF, LSDF, MSDF, MSTF, SV | NA | CA, QR, YU | + |  | + | + | + | + |  |  |  | + |  |  |
| Boraginaceae | *Heliotropium angiospermum* Murray | Cola de alacrán | Nej ma’ax | HE | CD, MG, MSDF, MSTF, LDF, LTF, SV | NA | CA, QR, YU | + | + | + | + | + | + | + | + | + | + | + | + |
| Boraginaceae | *Heliotropium ternatum* Vahl | - | Xikinche’ | SH | CD, LDF, LSDF, LIFT, MSTF, SV | NA | CA, QR, YU | + |  | + | + | + | + | + | + | + | + | + | + |
| Boraginaceae | *Rochefortia spinosa* (Jacq.) Urb. | Espina de brujo | Xikinche’ | SH | EVF, MSDF, MSTF | NA | CA, QR |  |  |  | + | + |  |  |  | + |  | + |  |
| Boraginaceae | *Tournefortia glabra* L. | Canzera | Chacah | SH | EVF, LDF, MSDF, MSTF, SV | NA | CA, QR, YU |  |  |  |  | + | + | + | + | + | + | + |  |
| Boraginaceae | *Tournefortia gnaphalodes* (L.) R. Br. ex Roem. & Schult. | Tabaquillo | K’an chooch | SH | CD, CDS, MG, MSTF | NA | CA, QR, YU |  | + | + | + | + |  | + | + | + | + | + | + |
| Boraginaceae | *Tournefortia hirsutissima* L. | Tlachichinole | - | SH | EVF, LDF, MSTF, SV | NA | CA, QR |  |  | + | + | + | + | + | + |  |  |  |  |
| Boraginaceae | *Tournefortia maculata* Jacq. | - | - | WC | MSTF | NA | CA, QR |  |  |  |  |  | + | + | + |  |  |  |  |
| Boraginaceae | *Tournefortia umbellata* Kunth | - | - | WC | LDF, MSTF | NA | CA, QR, YU |  |  |  | + | + | + | + | + |  |  |  |  |
| Boraginaceae | *Tournefortia volubilis* L. | Bejuco de mico | - | WC | EVF, LDF, LSDF, LIFT, MDF, MSDF, SV | NA | CA, QR, YU |  |  |  | + | + | + | + | + | + | + | + |  |
| Boraginaceae | *Varronia curassavica* Jacq. | - | K’opeche’ | SH | LDF, LSDF, LIFT, LTF, MSDF, MSTF, SV | NA | CA, QR, YU | + |  |  |  | + | + | + | + | + | + | + | + |
| Boraginaceae | *Varronia globosa* Jacq. | Orégano silvestre | Xopche’ | SH | CD, CDS, LDF, MG MSDF, MSTF, SV | NA | CA, QR, YU |  |  |  |  |  | + | + | + | + | + | + | + |
| Brassicaceae | *Cakile lanceolata* (Willd.) O. E. Schulz subsp. *alacranensis* (Millsp.) Rodman | - | Xaal | HE | CD | NA, EN | CA, YU |  |  |  | + | + |  |  |  |  |  |  |  |
| Burseraceae | *Bursera schlechtendalii* Engl. | - | Sak chakaj | SH | LDF, LSDF | NA | CA, YU |  |  | + |  |  |  |  |  |  |  |  |  |
| Burseraceae | *Bursera simaruba* (L.) Sarg. | Palo mulato | Chakaj | TR | EVF, LDF, LDFCC, LIFT, MSTF, SV | NA | CA, QR, YU |  |  |  | + | + |  |  |  |  |  |  |  |
| Burseraceae | *Protium copal* (Schltdl. & Cham.) Engl. | Copal | Sak chakaj | TR | LTF, MSTF | NA | CA, QR, YU | + | + | + |  |  |  |  |  |  |  |  |  |
| Buxaceae | *Buxus bartlettii* Standl. | - | - | SH | MSDF | NA | QR, YU |  | + | + |  |  |  | + | + |  |  | + | + |
| Cactaceae | *Hylocereus undatus* (Haw.) Britton & Rose | Pitahaya | - | HE | LDF, SV | NA | CA, QR, YU |  |  |  |  | + | + | + | + |  |  |  |  |
| Cactaceae | *Nopalea gaumeri* Britton & Rose | Nopal | - | SH | LIFT, LDF, MDF, MSDF | NA | QR, YU | + |  |  |  |  |  |  |  |  |  |  |  |
| Cannabaceae | *Celtis iguanaea* (Jacq.) Sarg. | Iguanero | Ts'i muk | SH | CD, LDF, MSTF | NA | CA, QR, YU | + | + | + | + | + | + | + | + | + | + | + | + |
| Cannabaceae | *Celtis trinervia* Lam. | - | Ta'an che' | TR | MSDF, MSTF | NA | CA, QR, YU |  |  |  | + |  | + | + | + | + |  |  |  |
| Cannabaceae | *Trema micrantha* (L.) Blume | Zapote blanco | - | TR | EVF, LDF, MSTF, SV | NA | CA, QR, YU | + | + | + | + | + | + | + | + | + | + | + | + |
| Capparaceae | *Crateva tapia* L. | Tepemezquite | - | TR | LDF, MSDF | NA | CA, QR |  | + | + | + |  |  |  |  |  | + | + |  |
| Capparaceae | *Cynophalla flexuosa* (L.) J. Presl | Chupamiel | Chuchuk che' | SH | CDS, EVF, LDF, MG, MSDF, MSTF | NA | CA, QR, YU |  |  |  | + | + | + | + | + |  |  | + |  |
| Capparaceae | *Cynophalla verrucosa* (Jacq.) J. Presl | - | - | SH | LDF | NA | CA, QR |  |  |  | + | + | + | + |  |  |  |  |  |
| Capparaceae | *Neocapparis pachaca* (Kunth) Cornejo | - | Chooch kitam | TR | LDF, MSTF | NT | CA, QR, YU |  | + | + | + |  |  |  |  | + |  |  |  |
| Capparaceae | *Quadrella incana* (Kunth) H. H. Iltis & X. | - | Bojk'anche' | TR | CD, LDF, MSTF, MG, SAV | NA | CA, QR, YU |  |  |  | + | + | + |  |  |  | + |  |  |
| Caricaceae | *Carica papaya* L. | Papaya | Ch’iich’ | TR | EVF, LDF, MSTF, SV | NA | CA, QR, YU | + | + | + | + | + | + | + | + | + | + | + | + |
| Caricaceae | *Jacaratia mexicana* A. CD. | Bonete | Ku'umche' | TR | LDF, MDF | NA | CA, QR, YU | + |  | + |  |  |  |  |  |  |  | + | + |
| Celastraceae | *Crossopetalum parviflorum* (Hemsl.) Lundell | Pinta uña | - | SH | CDS, LDF, MSDF, MSTF | NA | CA, QR, YU |  |  | + | + |  | + | + |  |  | + |  |  |
| Celastraceae | *Crossopetalum rhacoma* Crantz | Nopal | Chaklolmakal | SH | CD, CDS, LDF, SV | NA | CA, QR, YU |  |  |  | + | + |  | + |  |  | + |  |  |
| Celastraceae | *Elaeodendron xylocarpum* (Vent.) DC. | cheechem blanco | - | TR | CDS, LDF, MG, MSDF, MSTF | NA | CA, QR, YU |  | + | + | + | + |  |  | + |  |  |  |  |
| Chrysobalanaceae | *Chrysobalanus icaco* L. | Icaco | - | SH | LSDF, MG, RV | NA | CA, QR, YU | + | + | + | + | + | + | + | + |  |  | + |  |
| Chrysobalanaceae | *Couepia polyandra* (Kunth) Rose | - | Us piib | TR | EVF | NA | CA, QR, YU |  |  |  |  |  | + |  |  |  | + | + |  |
| Combretaceae | *Combretum formosum* G. Don | Árbol de la peineta | Cepillo' xiw | WC | LDF, MSTF | NA | CA, QR | + | + |  |  |  |  |  |  |  |  |  |  |
| Combretaceae | *Combretum fruticosum* (Loefl.) Stuntz | Peine de mico | - | WC | MSTF | NA | CA, QR, YU | + | + | + |  |  |  |  |  |  |  |  |  |
| Combretaceae | *Combretum laxum* Jacq. | - | - | WC | LDF, MSTF | NA | CA, QR |  |  |  | + | + |  |  |  | + |  |  |  |
| Combretaceae | *Conocarpus erectus* L. | Mangle botoncillo | K'an che' | H | AV, CD, LDF, LIFT, MG, MSTF | NA | CA, QR, YU |  |  |  |  | + | + | + | + |  |  |  |  |
| Combretaceae | *Laguncularia racemosa* (L.) C. F. Gaertn. | Mangle blanco | Sak okom | TR | CD, LIFT, MG | NA | CA, QR, YU |  |  |  |  | + | + | + |  |  |  |  |  |
| Combretaceae | *Terminalia buceras* (L.) C. Wright | Almendro de Río | Pukte’ | TR | EVF, LIFT, LDF, MSTF | NA | CA, QR, YU | + | + | + | + | + |  |  |  |  |  | + | + |
| Combretaceae | *Terminalia catappa* L. | Almendro | - | TR | LDF, SV | CU | CA, QR, YU |  | + |  |  |  | + |  |  | + | + |  |  |
| Combretaceae | *Terminalia molinetii* M. Gómez | Almendro | - | TR | LIFT, SAV | NA | CA, QR |  |  |  |  |  | + | + |  |  |  |  |  |
| Commelinaceae | *Callisia repens* (Jacq.) L. | - | Paj ts'a | HE | LDF, MSDF, SV | NA | CA | + | + | + |  |  |  |  |  |  |  |  |  |
| Commelinaceae | *Commelina erecta* L. | Flor de la virgen | Nuub | HE | CD, LDF, SV | NA | CA, QR, YU |  | + | + | + | + | + | + | + | + | + | + | + |
| Connaraceae | *Rourea glabra* Kunth | - | wayuum aak’ | HC | MSTF, EVF, SV | NA | CA, QR, YU | + |  | + | + | + |  |  |  |  |  |  |  |
| Convolvulaceae | *Aniseia martinicensis* (Jacq.) Choisy. | - | - | HC | LIFT | NA | CA, QR |  |  |  |  |  |  |  |  | + | + | + | + |
| Convolvulaceae | *Camonea umbellata* (L.) A.R. Simões & Staples | Campanillas | - | HC | LDF, LIFT, MSTF, SV | NA | CA, QR | + | + |  | + | + |  | + | + | + |  | + | + |
| Convolvulaceae | *Convolvulus nodiflorus* Desr. | - | Sak lool aak | HC | LDF, LIFT, SV | NA | CA, QR, YU | + |  |  |  |  |  |  |  |  |  | + | + |
| Convolvulaceae | *Distimake aegyptius* (L.) A.R. Simões & Staples | - | Tso' ots' aak' | HC | LDF, MG, SV | NA | CA, QR, YU |  |  |  | + |  |  | + |  |  | + | + | + |
| Convolvulaceae | *Distimake cissoides* (Lam.) A.R.Simões & Staples | - | P'aak aak' | HC | CDS, LDF, LDFCC, LIFT, MSDF, MSTF, SV | NA | CA, QR, YU |  |  |  |  |  |  |  | + | + | + | + |  |
| Convolvulaceae | *Distimake dissectus* (Jacq.) A.R. Simões & Staples | - | - | HC | LDF, MSTF, SV | NA | CA, QR, YU |  |  | + | + | + | + | + | + | + | + | + |  |
| Convolvulaceae | *Distimake tuberosus* (L.) Simões & Staples | - | - | HC | LDF, LIFT, MSTF, SV | NA | CA, QR, YU | + |  |  |  |  |  |  |  |  | + | + | + |
| Convolvulaceae | *Evolvulus alsinoides* (L.) L. | Ojitos azulitos | Xia xiiw | HC | CDS, LDF, LIFT, MG | NA | CA, QR, YU |  | + | + | + |  | + | + |  | + | + | + | + |
| Convolvulaceae | *Evolvulus convolvuloides* (Willd. ex Schult.) Stearn | - | Jaway aak | HC | LDF | NA | YU |  |  |  |  |  |  | + |  | + | + | + |  |
| Convolvulaceae | *Evolvulus nummularius* (L.) L. | - | - | HC | LDF, MSTF | NA | CA, QR, YU | + | + | + | + |  |  |  | + | + | + | + | + |
| Convolvulaceae | *Evolvulus sericeus* Sw. | - | Jaway aak' | HC | LDF, MSTF | NA | CA, QR, YU |  |  |  |  |  | + |  | + |  |  |  | + |
| Convolvulaceae | *Ipomoea alba* L. | Trompillo | Sak p’uul | HC | CDS, LDF, LDFCC, LIFT, MSDF, MSTF, SV | NA | CA, QR, YU | + |  |  | + |  |  |  |  | + | + | + |  |
| Convolvulaceae | *Ipomoea batatas* (L.) Lam. | Batata | Iz | HC | LDF, MSDF, MSTF, SV | NT | CA, QR, YU | + | + | + |  |  |  | + | + |  |  | + | + |
| Convolvulaceae | *Ipomoea carnea* Jacq. ssp. *carnea* | Campanilla | Ke'Elil | SH | CDS, LDF, LDFCC, LIFT, SV | NA | CA, QR, YU | + | + | + | + | + | + |  |  | + | + | + |  |
| Convolvulaceae | *Ipomoea carnea* Jacq. ssp. *fistulosa* (Mart. ex Choisy) D. F. Austin | - | - | HC | LIFT, LDF, SV | NA | CA, QR, YU | + | + | + | + | + | + |  |  |  |  | + | + |
| Convolvulaceae | *Ipomoea clavata* (G. Don) Ooststr. ex J. F. Macbr. | - | Ulu’um ja’ | HC | LDF, MSDF, MSTF, SV | NA | CA, QR, YU |  | + |  |  |  |  |  |  |  | + | + |  |
| Convolvulaceae | *Ipomoea crinicalyx* S. Moore | Trompillón | - | HC | LDF, MSDF, MSTF, SV | NA | CA, QR, YU | + |  | + | + | + |  |  | + | + | + | + | + |
| Convolvulaceae | *Ipomoea hederifolia* L. | - | Ya’ax ka’anil | HC | CDS, LDF, LDFCC, MSDF, MSTF, SV | NA | CA, QR, YU | + | + | + |  |  |  |  |  | + | + | + | + |
| Convolvulaceae | *Ipomoea heterodoxa* Standl. & Steyerm. | - | - | HC | LDF, MSDF, MSTF, SV | NA | CA, QR, YU |  | + |  |  |  |  |  | + | + | + | + | + |
| Convolvulaceae | *Ipomoea imperati* (Vahl) Griseb. | - | Shantalo | HC | CD, CDS, MG | NA | CA, QR |  | + | + |  |  |  |  |  | + |  | + |  |
| Convolvulaceae | *Ipomoea indica* (Burm. f.) Merr. | - | - | HC | CDS, LDF, LDFCC, MSDF, MSTF, SV | NA | CA, QR, YU | + | + | + | + |  | + | + | + | + | + | + | + |
| Convolvulaceae | *Ipomoea muricata* (L.) Jacq. | - | Choko kat | HC | LDF, SV | NA | YU |  |  |  |  |  |  |  |  |  | + | + |  |
| Convolvulaceae | *Ipomoea pes-caprae* (L.) R. Br. | Riñonina | - | HC | LDF, MSDF, MSTF, SV | NA | CA, QR, YU | + |  | + | + | + |  |  | + | + | + | + | + |
| Convolvulaceae | *Ipomoea quamoclit* L. | Cundeamor | - | HC | LDF, MSDF, MSTF, SV | NA | CA, QR, YU |  |  |  |  |  |  |  | + | + | + |  |  |
| Convolvulaceae | *Ipomoea sagittata* Poir. | - | - | HC | MG, RV | NA | CA, QR, YU |  |  |  |  | + |  |  | + | + |  |  |  |
| Convolvulaceae | *Ipomoea splendor-sylvae* House | - | Is aak’il | HC | LDF, MSDF, MSTF, SV | NA | CA, QR, YU | + | + |  |  |  |  |  |  |  | + |  |  |
| Convolvulaceae | *Ipomoea steerei* (Standl.) L. O. Williams | - | - | HC | CDS, LDF, LDFCC, MG, MSDF, MSTF, SV | NA, EN | CA, QR, YU |  |  |  |  | + | + | + | + | + | + |  |  |
| Convolvulaceae | *Ipomoea tiliacea* (Willd.) Choisy | Contlapani | - | HC | MSDF, MSTF, SV | NA | CA, QR |  |  |  |  |  | + |  |  |  |  |  |  |
| Convolvulaceae | *Ipomoea tricolor* Cav. | Badoh negro | - | HC | LDF, MSDF, MSTF, SV | NA | CA, YU | + | + | + |  |  |  |  |  |  |  | + | + |
| Convolvulaceae | *Ipomoea triloba* L. | Flor de la mañana | - | HC | LDF, LDFCC, MSDF, MSTF, SV | NA | CA, QR, YU | + |  | + |  | + |  | + |  | + | + | + |  |
| Convolvulaceae | *Ipomoea violacea* L. | Gloria de la mañana | - | HC | CD, MG | NA | CA, QR, YU | + |  | + | + | + | + | + |  | + | + | + | + |
| Convolvulaceae | *Ipomoea heptaphylla* Sweet | - | Choko kat | HC | LDF, LIFT, SV | NA | CA, YU | + |  |  |  |  |  |  |  |  | + | + | + |
| Convolvulaceae | *Ipomoea peteri* (Kuntze) Staples & Govaerts | - | - | HC | LDF, LIFT, MSDF, MSTF, SV | NA | CA, QR, YU |  |  |  |  | + |  | + | + | + | + | + | + |
| Convolvulaceae | *Itzaea sericea* (Standl.) Standl. & Steyerm. | - | - | HC | MSTF, SV | NA | CA, QR, YU |  |  |  |  |  |  |  |  | + |  |  |  |
| Convolvulaceae | *Jacquemontia havanensis* (Jacq.) Urb. | - | - | HC | CD | NA | QR, YU | + | + | + |  |  |  |  |  | + | + | + | + |
| Convolvulaceae | *Jacquemontia oaxacana* (Meisn.) Hallier f. | - | - | HC | LDF, MSTF | NA | CA |  |  |  |  |  |  |  |  |  | + | + |  |
| Convolvulaceae | *Jacquemontia ovalifolia* (Choisy) Hallier f. subsp. *obcordata* (Millsp.) K. R. Robertson | - | - | HC | CDS, LDF | NA | YU |  | + | + |  |  |  |  |  |  |  |  |  |
| Convolvulaceae | *Jacquemontia tamnifolia* (L.) Griseb. | - | Ya`ax-ebil | HC | LDF, LIFT, MSDF, SV | NA | CA, QR, YU | + | + |  |  |  |  |  | + | + | + | + | + |
| Convolvulaceae | *Jacquemontia verticillata* (L.) Urb | - | Sik’ ke’el | HC | LIFT, LDF, MSDF, MSTF | NA | CA, QR, YU | + | + | + | + |  |  | + | + | + | + | + | + |
| Convolvulaceae | *Jacquemontia pentanthos* (Jacq.) G. Don | - | Aak’il xiiw | HC | CDS, LDF | NA | CA, QR, YU | + | + | + | + |  |  |  |  |  | + | + | + |
| Convolvulaceae | *Operculina pinnatifida* (Kunth) O’Donell | - | Aak’ k’oon tin k’iin | HC | LDF, MSDF | NA | CA, YU |  |  | + | + | + | + | + | + | + | + |  |  |
| Convolvulaceae | *Turbina corymbosa* (L.) Raf. | - | Xtabentun | HC | CD, EVF, LDF, MSTF | NA | CA, QR, YU | + | + |  | + |  | + |  |  |  | + | + | + |
| Cucurbitaceae | *Cayaponia racemosa* (Mill.) Cong. | Sandía ratón | Ta’kéej | HC | LDF, SV | NA | CA, QR, YU | + | + | + |  |  |  |  |  |  | + | + |  |
| Cucurbitaceae | *Citrullus lanatus* (Thunb.) Matsum. & Nakai | Sandía | - | HE | MDF, SV | CU | CA, QR, YU |  |  |  |  |  |  | + | + | + |  |  |  |
| Cucurbitaceae | *Cucumis melo* L. | Melón | - | HE | CD, CDS, SV | CU | CA, QR, YU | + | + | + | + |  |  | + | + | + | + |  |  |
| Cucurbitaceae | *Cucumis sativus* L. | Pepino | - | HE | SV | CU | CA, QR, YU |  |  |  |  |  |  | + |  |  |  |  |  |
| Cucurbitaceae | *Cucurbita argyrosperma* K. Koch subsp. *argyrosperma* | Chihua | - | HE | LDF, SV | NT | CA, QR, YU |  |  |  |  | + | + |  | + |  | + |  |  |
| Cucurbitaceae | *Cucurbita moschata* Duchesne | Calabaza | - | HE | LDF | CU | CA, QR, YU |  |  |  |  | + |  |  | + | + | + |  |  |
| Cucurbitaceae | *Cucurbita pepo* L. subsp. *pepo* | Calabaza de castilla | Tzol | HE | SV | CU | CA, QR, YU |  |  |  |  |  |  |  |  | + |  |  |  |
| Cucurbitaceae | *Doyerea emetocathartica* Grosourdy | - | Ciz can | WC | LDFCC, SV | NA | CA, QR, YU |  | + | + | + | + | + |  |  |  |  |  | + |
| Cucurbitaceae | *Luffa cylindrica* (L.) M. Roem. | Estropajo | Jabaplat | HC | MSDF, SV | CU | CA, QR, YU |  |  | + |  |  | + |  |  | + |  |  |  |
| Cucurbitaceae | *Melothria pendula* L. | Sandía stulub | K’uum tulub | HE | LDF, LIFT, MSDF, MSTF | NA | CA, QR, YU | + |  | + | + | + | + | + | + | + | + | + | + |
| Cucurbitaceae | *Momordica charantia* L. | - | Yakunah-ax | HC | LDF, SV | NT | CA, QR, YU |  | + |  |  |  |  |  |  | + |  |  |  |
| Cucurbitaceae | *Polyclathra cucumerina* Bertol | Calabacilla | Ponpol tsiimim | HC | MSDF, MSTF | NA | CA, QR, YU |  |  |  |  | + |  |  |  |  |  | + |  |
| Cucurbitaceae | *Rytidostylis gracilis* Hook & Arn. | Chayotillo | - | HE | LDF, LDFCC | NA | CA |  |  |  |  |  |  |  |  | + | + | + | + |
| Cucurbitaceae | *Sicana odorifera* (Vell.) Naudin | Calabaza melona | - | HC | MSTF | CU | YU | + | + |  |  |  |  |  |  |  |  |  | + |
| Cucurbitaceae | *Sicydium tamnifolium* (Kunth) Cogn. | - | Chak mots | HC | EVF, LDF, MSDF, SV | NA | CA, QR, YU | + |  |  | + | + |  | + | + | + | + | + | + |
| Cyperaceae | *Cladium jamaicense* Crantz | Cortadera | Jol che' | HE | CD, MG, MSTF, SAV, SV | NA | CA, QR, YU |  | + | + | + | + | + | + | + |  |  |  |  |
| Cyperaceae | *Cyperus articulatus* L. | Tule | Ta'uuk | HE | CD, LDF, LIFT, MG, MSTF, SV | NA | CA, QR, YU | + | + | + | + | + | + | + | + | + | + | + | + |
| Cyperaceae | *Eleocharis cellulosa* Torr. | - | - | HE | AV, LIFT, MG | NA | CA, QR, YU |  | + | + | + | + | + | + | + | + | + |  |  |
| Cyperaceae | *Eleocharis elegans* (Kunth) Roem. & Schult. | - | Polol | HE | AV, LDF, LIFT, MG | NA | CA, QR |  |  | + | + |  |  |  |  | + |  |  |  |
| Cyperaceae | *Eleocharis geniculata* (L.) Roem. & Schult. | Popoque | - | HE | AV, LDFCC, MSTF | NA | CA, QR, YU | + | + | + | + | + | + | + | + | + | + | + |  |
| Cyperaceae | *Eleocharis montana* (Kunth) Roem. & Schult. | - | - | HE | LIFT, SV | NA | CA |  |  |  | + | + | + | + | + | + | + |  |  |
| Cyperaceae | *Eleocharis mutata* (L.) Roem. & Schult. | - | - | HE | LDF, MG, SAV | NA | CA, QR, YU |  |  | + | + | + | + | + | + | + | + | + |  |
| Cyperaceae | *Rhynchospora cephalotes* (L.) Vahl | Zacatón | - | HE | EVF, LIFT, MSTF | NA | CA, QR, YU |  | + | + | + | + | + | + | + | + | + | + | + |
| Cyperaceae | *Torulinium odoratum* (L.) SS Hooper | - | - | HE | CD, LDF, LIFT, MG, MSTF, SV | NA | CA, QR, YU | + | + | + | + | + | + | + | + | + | + | + | + |
| Dilleniaceae | *Davilla kunthii* A. St.-Hil. | Bejuco de tachicón | - | HE | LDF, LIFT, MSTF | NA | CA, QR | + | + | + | + | + |  | + | + | + | + | + | + |
| Dilleniaceae | *Doliocarpus dentatus* (Aubl.) Standl. | Bejuco de agua | - | WC | CD, MSTF | NA | CA |  |  | + | + |  |  |  |  |  |  | + |  |
| Dioscoreaceae | *Dioscorea alata* L. | Ñame de agua | - | HC | EVF | CU | CA, QR, YU |  |  |  |  |  |  |  |  | + | + | + | + |
| Dioscoreaceae | *Dioscorea convolvulacea* Schltdl. & Cham. | - | - | HC | LDF, MSTF | NA | CA, YU |  |  |  |  |  |  |  |  |  | + | + | + |
| Dioscoreaceae | *Dioscorea densiflora* Hemsl. | Nacú liso | Aak’il | HC | LIFT | NA | CA | + | + |  |  |  |  |  |  |  | + |  | + |
| Dioscoreaceae | *Dioscorea floribunda* M. Martens & Galeotti | Barbasco | - | HC | LDF, LSDF, MSDF | NA | CA, YU | + | + |  |  |  |  | + |  | + | + | + | + |
| Dioscoreaceae | *Dioscorea gaumeri* Knuth | - | Makal kuch | HC | LTF, MSDF, MSTF, SV | NA, EN | CA, QR, YU | + |  |  |  |  |  | + | + |  | + | + |  |
| Dioscoreaceae | *Dioscorea matagalpensis* Uline | - | Makal kuch | HC | LDF, SV | NA | CA, QR, YU |  |  |  |  |  |  |  | + | + | + | + |  |
| Dioscoreaceae | *Dioscorea pilosiuscula* Bertero ex Spreng. | - | - | HC | LDF, MSDF, MSTF | NA | CA, QR, YU | + | + | + | + |  | + |  | + | + |  |  | + |
| Dioscoreaceae | *Dioscorea polygonoides* Humb. & Bonpl. ex Willd. | - | - | HC | LDF, MSDF | NA | CA, QR |  |  |  |  |  |  |  |  | + | + | + |  |
| Dioscoreaceae | *Dioscorea spiculiflora* Hemsl. | Nacú liso | Ak’il | HC | EVF, LDF | NA | CA, QR, YU |  | + |  |  | + | + |  |  | + | + | + | + |
| Ebenaceae | *Diospyros anisandra* S. F. Blake | - | K’aakal ché | SH | LDF, MG, MSDF, MSTF, SV | NA | CA, QR, YU |  | + |  |  | + | + |  |  | + |  |  |  |
| Ebenaceae | *Diospyros bumelioides* Standl. | Zapotillo | - | SH | LDF, LIFT | NA, EN | CA, QR | + | + |  |  |  | + | + | + |  | + |  |  |
| Ebenaceae | *Diospyros campechiana* Lundell | - | - | TR | LIFT | NA | CA |  |  |  |  |  |  | + |  |  |  |  |  |
| Ebenaceae | *Diospyros digyna* Jacq. | Zapote negro | Ta’uch | TR | LDF, LIFT, MG | NA | CA, QR, YU |  |  | + | + | + | + |  |  |  |  |  |  |
| Ebenaceae | *Diospyros salicifolia* Humb. & Bonpl. ex Willd. | Chocoyito | U chul che | TR | MSTF, MSDF | NA | QR, YU |  | + |  |  |  |  | + | + | + | + | + | + |
| Ebenaceae | *Diospyros tetrasperma* Sw. | - | Sip che’ | TR | CD, CDS, EVF, LDF, LIFT, MSTF | NA | CA, QR, YU |  |  |  | + | + | + | + | + | + |  |  |  |
| Ebenaceae | *Diospyros yatesiana* Standl | - | U chul che’ | TR | LDF, MSTF | NA | CA, QR, YU |  |  |  | + | + | + |  | + | + | + |  |  |
| Erythroxylaceae | *Erythroxylum confusum* Britton | Cascarillo | Tooso | HE | LDF, LIFT, MSTF | NA | CA |  |  | + |  |  |  | + | + |  |  |  |  |
| Euphorbiaceae | *Croton arboreus* Millsp. | - | Pak che’ | HE | EVF, LDF, MSDF | NA | CA | + | + | + | + | + |  | + |  |  | + | + | + |
| Euphorbiaceae | *Acalypha alopecuroidea* Jacq. | Cola de gato | - | HE | EVF, MSDF, SV | NA | CA, QR, YU |  |  |  |  |  | + | + | + | + | + |  |  |
| Euphorbiaceae | *Acalypha arvensis* Poepp. | - | - | HE | EVF, LTF, SV | NA | CA, QR | + | + | + | + | + | + | + | + | + | + | + | + |
| Euphorbiaceae | *Acalypha diversifolia* Jacq. | Papacamino | - | SH | MSDF | NA | CA, QR, YU | + | + | + | + | + |  |  |  |  |  |  |  |
| Euphorbiaceae | *Acalypha gaumeri* Pax & K. Hoffm. | - | - | SH | LDF, MSDF | NA, EN | CA, QR, YU | + |  | + | + | + | + | + | + |  |  |  |  |
| Euphorbiaceae | *Acalypha macrostachya* Jacq. | - | - | SH | EVF, LDF, MSTF | NA | YU |  | + |  | + |  |  | + | + |  |  |  |  |
| Euphorbiaceae | *Acalypha poiretii* Spreng. | - | - | SH | LDF | NA | YU |  |  |  |  |  |  |  | + |  | + |  |  |
| Euphorbiaceae | *Acalypha polystachya* Jacq. | - | - | HE | LDF | NA | YU |  |  |  |  |  |  |  | + | + |  |  | + |
| Euphorbiaceae | *Acalypha wilkesiana* Müll.Arg. | Acalifa | - | SH | SV | CU | CA, QR, YU | + | + | + | + | + | + | + | + | + | + | + | + |
|  |  | Espino blanco | - |  |  |  |  |  |  |  |  |  |  |  |  |  |  |  |  |
| Euphorbiaceae | *Adelia barbinervis* Schltdl. & Cham. | Caca de gallina | - | TR | LDF, MSDF, MSTF, SV | NA | CA, QR, YU |  | + | + | + |  | + | + |  |  |  | + |  |
| Euphorbiaceae | *Adelia oaxacana* (Müll. Arg.) Hemsl. | - | - | SH | LDF, MSDF, SV | NA | QR, YU | + | + | + | + | + | + | + |  |  |  |  |  |
| Euphorbiaceae | *Argythamnia lundellii* J. W. Ingram | - | - | SH | LDF, MSDF | NA, EN | CA, QR, YU |  |  |  |  |  | + |  |  |  |  | + | + |
| Euphorbiaceae | *Bernardia oblanceolata* Lundell | - | - | SH | CD, MSTF | NA | QR | + | + |  |  |  |  |  |  |  |  |  |  |
| Euphorbiaceae | *Caperonia palustris* (L.) A. St. Hil | - | - | HE | EVF, LDF, MSTF, SAV | NA | CA, QR, YU |  | + | + |  | + |  | + | + | + | + |  |  |
| Euphorbiaceae | *Cnidoscolus aconitifolius* (Mill.) I. M. Johnst | Chaya | - | SH | LDF, MSDF | NA | CA, QR, YU |  |  | + |  | + | + | + | + | + |  |  |  |
| Euphorbiaceae | *Cnidoscolus multilobus* (Pax) I. M. Johnst. | Cochaya silvestre | - | SH | MSTF | CU | CA, QR, YU |  | + | + |  |  |  |  |  |  |  |  |  |
| Euphorbiaceae | *Cnidoscolus souzae* McVaugh | - | - | SH | LSDF | NA, EN | CA, QR, YU | + | + | + | + | + | + | + |  |  | + | + | + |
| Euphorbiaceae | *Codiaeum variegatum* (L.) Rumph. ex A. Juss. | - | - | SH | SV | CU | CA, QR, YU |  |  | + | + |  |  | + |  | + |  |  |  |
| Euphorbiaceae | *Croton ameliae* Lundell | - | - | SH | LDF, MSDF | NA, EN | QR, YU |  |  | + | + | + | + |  |  |  |  |  |  |
| Euphorbiaceae | *Croton argenteus* L. | - | - | HE | EVF, MSDF, SV | NA | CA, QR, YU |  |  |  | + | + | + | + | + |  |  |  |  |
| Euphorbiaceae | *Croton chichenensis* Lundell | Xikin burro | Éck baalam | SH | LDF, MSTF | NA, EN | CA, QR, YU | + | + | + | + | + | + | + | + | + | + | + | + |
| Euphorbiaceae | *Croton cortesianus* Kunth | - | - | SH | LDF | NA | CA, QR, YU | + | + | + |  |  | + |  |  | + | + |  | + |
| Euphorbiaceae | *Croton glabellus* L. | - | - | SH | LDF, SV | NA | CA, QR, YU |  |  | + | + | + | + | + |  | + |  |  |  |
| Euphorbiaceae | *Croton humilis* L. | - | Iik aban | SH | LDF, SV | NA | CA, QR, YU | + | + | + |  | + | + | + | + | + | + | + | + |
| Euphorbiaceae | *Croton icche* Lundell | - | Iiik che’ | SH | LDF, MSDF | NA, EN | CA, QR, YU |  |  | + |  |  |  | + | + |  |  | + |  |
| Euphorbiaceae | *Croton itzaeus* Lundell | - | - | SH | EVF, LDF, SAV | NA, EN | CA, QR, YU |  |  | + | + | + | + |  |  |  |  |  |  |
| Euphorbiaceae | *Croton lobatus* L. | - | - | SH | CD, LDF, MSDF, MSTF | NA | CA, QR, YU |  |  |  |  |  | + | + | + |  |  |  | + |
| Euphorbiaceae | *Croton malvaviscifolius* Millsp. | - | - | SH | MSDF, LDF | NA | CA, QR, YU |  | + |  |  |  |  | + | + | + | + |  |  |
| Euphorbiaceae | *Croton millspaughii* Standl. | Cascarilla | Éek baalam | SH | LDF | NA, EN | QR, YU |  |  | + |  | + | + |  |  |  |  |  |  |
| Euphorbiaceae | *Croton nitens* Sw. | - | - | SH | MSDF, LDF | NA | CA, QR, YU |  |  |  |  |  | + |  |  | + |  |  | + |
| Euphorbiaceae | *Croton niveus* Jacq. | - | - | SH | LDF, MSDF | NA | CA, QR |  | + | + | + | + |  |  |  |  |  |  |  |
| Euphorbiaceae | *Croton oerstedianus* Müll. Arg. |  | Sen k’ook | SH | EVF, LDF, MSDF | NA | CA, QR, YU |  |  |  |  |  | + | + | + | + | + |  |  |
| Euphorbiaceae | *Croton peraeruginosus* Croizat |  | Xikin | SH | LDF, MG, MSDF | NA, EN | CA, QR, YU | + | + | + | + | + | + | + | + | + | + |  | + |
| Euphorbiaceae | *Croton punctatus* Jacq. | Hierba de jabalí | Sak chuum | SH | CD, LDF, MG, MSDF | NA | CA, QR, YU |  |  |  | + | + | + | + | + | + | + | + | + |
| Euphorbiaceae | *Croton reflexifolius* Kunth | - | Ko’ok | SH | MSDF, MSTF, SV | NA | CA, QR, YU | + | + | + | + | + | + | + | + |  |  | + |  |
| Euphorbiaceae | *Croton schiedeanus* Schltdl. | - | - | TR | EVF, LDF, MSDF, SV | NA | CA, QR, YU |  |  |  |  | + | + | + | + | + | + |  | + |
| Euphorbiaceae | *Croton sutup* Lundell | - | Sutup | SH | LDF, MSDF | NA, EN | CA, QR, YU |  | + | + | + | + | + |  |  |  |  |  |  |
| Euphorbiaceae | *Croton yucatanensis* Lundell | - | Chiim kuuts | SH | LDF, SV | NA | YU |  |  | + |  | + | + |  |  |  |  |  |  |
| Euphorbiaceae | *Croton flavens* L. | - | - | SH | LDF, SV | CU | CA, QR, YU |  |  |  |  | + | + |  |  | + | + | + |  |
| Euphorbiaceae | *Enriquebeltrania crenatifolia* (Miranda) Rzed. | - | - | SH | CD, LDF | NA, EN | CA, QR, YU |  |  | + | + | + |  | + | + | + | + |  | 1 |
| Euphorbiaceae | *Euphorbia anychioides* Boiss. | - | Kabal chakaj | HE | LSDF, LIFT | NA | CA, YU |  |  |  |  |  |  | + |  |  | + |  |  |
| Euphorbiaceae | *Euphorbia armourii* Millsp. | - | - | HE | LTF, MSDF | NA | CA, QR, YU |  | + |  | + |  |  |  |  |  | + | + |  |
| Euphorbiaceae | *Euphorbia blodgettii* Engelm. ex Hitchc. | - | - | HE | CD, LDF, MG | NA | QR, YU |  |  |  | + |  | + | + |  |  | + | + | + |
| Euphorbiaceae | *Euphorbia dentata* Michx. | - | We' ech che‘ | HE | EVF | CU | QR, YU |  |  |  |  |  |  |  | + | + | + |  |  |
| Euphorbiaceae | *Euphorbia gaumeri* Millsp. | - | We’ ech che‘ | HE | LDF, MSDF | NA, EN | QR, YU | + | + | + |  |  |  |  |  |  | + |  |  |
| Euphorbiaceae | *Euphorbia humayensis* Brandegee |  |  | HE | LDF | NA | YU |  |  |  |  |  |  |  |  |  |  |  |  |
| Euphorbiaceae | *Euphorbia hyssopifolia* L. | Golondrina | Xana mukuy | HE | CD, EVF, LDF, RV, SAV | NA | CA, QR, YU | + |  | + | + | + | + | + |  | + | + |  | + |
| Euphorbiaceae | *Euphorbia lasiocarpa* Klotzsch | - | - | HE | LSDF, SV | NA | CA, YU |  |  | + |  |  |  |  |  |  |  | + | + |
| Euphorbiaceae | *Euphorbia marginata* Pursh | - | - | HE | RV | NA | CA |  |  |  |  | + |  |  | + |  | + |  |  |
| Euphorbiaceae | *Euphorbia mesembryanthemifolia* Jacq. |  | - | HE | CD | NA | CA, QR, YU |  | + | + | + | + | + | + | + |  | + | + |  |
| Euphorbiaceae | *Euphorbia milii* Des Moul. | Corona de cristo | - | HE | - | CU | CA, QR, YU | + | + | + | + | + | + | + | + | + | + | + | + |
| Euphorbiaceae | *Euphorbia ocymoidea* L. | - |  | HE | LDF, MSDF | NA | CA, QR, YU | + | + |  | + |  |  |  |  |  |  | + |  |
| Euphorbiaceae | *Euphorbia schlechtendalii* Boiss. var. *schlechtendalii* | - | Sak chakaj | HE | CD, LDF | NA | CA, QR, YU | + | + | + | + | + |  |  |  |  |  | + | + |
| Euphorbiaceae | *Euphorbia trichotoma* Kunth | - | - | HE | CD | NA | QR |  |  |  |  |  |  |  |  | + |  | + |  |
| Euphorbiaceae | *Euphorbia villifera* Scheele | - | - | HE | LDF, SV | NA | YU | + |  |  |  | + |  |  |  | + |  |  |  |
| Euphorbiaceae | *Euphorbia xbacensis* Millsp. | - | Xana mukuy | HE | LDF, SAV | NA, EN | CA, YU | + |  |  |  |  |  | + |  |  | + | + |  |
| Euphorbiaceae | *Euphorbia yucatanensis* (Millsp.) Standl. | - | - | HE | LDF, LDFCC, SV | NA, EN | QR, YU |  |  |  |  |  |  |  |  | + | + |  |  |
| Euphorbiaceae | *Euphorbia mendezii* Boiss. | - | - | HE | CD, LDF | NA | CA, QR, YU |  |  | + |  |  |  | + | + | + | + | + | + |
| Euphorbiaceae | *Gymnanthes lucida* Sw. | - | Ya'ay tiik | HE | CD, EVF, LDF, MSDF | NA | CA, QR, YU |  | + |  |  | + | + | + |  |  | + |  |  |
| Euphorbiaceae | *Jatropha curcas* L. | - | Xu'ul | SH | SV | CU | CA, QR, YU |  |  |  |  |  | + | + | + |  |  |  |  |
| Euphorbiaceae | *Jatropha gaumeri* Greenm. | - | Pomol che’ | TR | LDF, LDFCC, MSDF, SV | NA | CA, QR, YU | + | + | + | + |  | + |  |  |  | + |  |  |
| Euphorbiaceae | *Manihot esculenta* Crantz | Yuca | - | SH | SV | CU | CA, QR, YU |  |  |  |  |  |  |  | + | + | + | + | + |
| Euphorbiaceae | *Ricinus communis* L. | Higuerilla | Éek lu'um | SH | LDF, MG, MSTF, SV | NT | CA, QR, YU | + | + | + | + | + | + | + | + | + | + | + | + |
| Euphorbiaceae | *Sebastiania adenophora* Pax & K. Hoffm. | Chechem blanco | Sak chée chem | TR | LDF, MSTF, SAV, SV | NA | CA, QR, YU |  |  | + | + | + | + |  |  |  |  |  |  |
| Fabaceae | *Acacia melanoxylon* R. Br. | - | - | SH | SV | CU | CA, YU |  |  |  |  |  |  |  |  | + |  |  |  |
| Fabaceae | *Acaciella angustissima* var. *angustissima* (Mill.) Britton & Rose |  | Waaxim, xáax | TR | LDF, LIFT, MG, MSTF, MSDF, SV | NA | CA, QR, YU | + |  |  | + | + | + | + | + | + | + | + |  |
| Fabaceae | *Aeschynomene americana* L. | Tamarindo | Xiiw | SH | LIFT, LDF, MSTF, SV | NA | CA, QR, YU | + | + |  |  |  |  |  |  |  | + | + | + |
| Fabaceae | *Aeschynomene fascicularis* Schltdl. & Cham. | - | Kabal | SH | MSDF | NA | CA, QR, YU |  |  |  | + | + | + |  |  |  |  | + |  |
| Fabaceae | *Albizia niopoides* (Spruce ex Benth.) Burkart var. *niopoides* | Cantemo | Waaxim | TR | LDF, MSDF, SV | NA | CA, QR |  |  |  |  |  |  |  |  |  |  |  |  |
| Fabaceae | *Albizia tomentosa* (Micheli) Standl. | Palo de sangre | Juub che' | TR | EVF, MSDF | NA | CA, QR, YU |  |  | + | + | + | + | + | + |  |  |  |  |
| Fabaceae | *Andira inermis* (W. Wright) Kunth ex DC. subsp. *inermis* | - | Ya'abo'ob | TR | MSTF | NA | CA |  |  |  | + |  |  | + | + |  |  |  |  |
| Fabaceae | *Apoplanesia paniculata* C. Presl | - | Chulúul, k'i'ik' che' | TR | LDF, MSDF, SV | NA | CA, QR, YU |  |  |  |  |  | + | + | + | + | + |  |  |
| Fabaceae | *Ateleia cubensis* Griseb. | - | - | TR | LIFT | NA | CA, QR, YU | + |  |  |  |  |  |  |  |  |  | + | + |
| Fabaceae | *Bauhinia divaricata* L. | Pata de vaca | Ts' ulub took' | TR | LDF, MSDF, MSTF, SV | NA | CA, QR, YU | + | + | + | + | + | + | + | + | + | + | + | + |
| Fabaceae | *Bauhinia herrerae* (Britton et Rose) Standl. et Steyerm. | Pata de cochino blanco | Boox ts’ ulub took | WC | EVF, LDF, LIFT, MSTF | NA | CA, QR, YU | + | + | + |  | + |  |  |  | + | + | + | + |
| Fabaceae | *Bauhinia jenningsii* Paul G. Wilson | Pata de venado | Ts’ ulub took’ | SH | EVF, LDF, MSTF | NA | CA, QR | + | + | + | + | + | + |  | + | + | + | + | + |
| Fabaceae | *Bauhinia ungulata* L. | Cola de gallo | Chak  ts’ ulub took’ | SH | LDF, MSDF, MSTF, SV | NA | CA, QR, YU | + | + | + | + |  |  |  |  |  | + | + | + |
| Fabaceae | *Caesalpinia gaumeri* Greenm. | - | Kitanché | TR | LDF, MSTF | NA | CA, QR, YU | + | + | + | + | + |  | + | + | + | + | + | + |
| Fabaceae | *Caesalpinia pulcherrima* (L.) Sw. | Tronadora | Chak sin in | TR | MSTF, SV | NA | CA, QR, YU |  |  | + | + | + |  |  |  | + | + |  | + |
| Fabaceae | *Caesalpinia vesicaria* L. | Mareña | Ya’ax k’iin che | TR | CDS, LDF, LIFT, LDFCC, MG, MSTF | NA | CA, QR, YU | + | + | + |  |  |  |  | + | + | + | + | + |
| Fabaceae | *Caesalpinia yucatanensis* Greenm. |  | Taa k'in che' | TR | LDF, MSTF, SV | NA | CA, QR, YU | + | + | + |  |  |  |  |  |  |  | + | + |
| Fabaceae | *Caesalpinia mollis* (Kunth) Spreng. | Viga | Chak te’ | TR | LSDF, LDF | NA | CA, QR, YU |  |  | + | + | + | + | + | + |  |  |  |  |
| Fabaceae | *Cassia fistula* L. | Lluvia de oro | - | TR | LDF, SV | CU | CA, QR, YU |  |  |  | + |  |  |  |  |  |  |  |  |
| Fabaceae | *Centrosema schottii* (Millsp.) K. Schum. | Chochito | Bu’ul ch’o’ | HC | LDF, LSDF, MG, MSDF | NA | CA, QR, YU | + | + | + |  | + |  |  |  |  | + | + | + |
| Fabaceae | *Centrosema virginianum* (L.) Benth. | - | Bu’ul che | HC | LDF, MSTF, SV | NA | CA, QR, YU | + |  |  |  |  | + | + | + | + | + | + | + |
| Fabaceae | *Chaetocalyx scandens* (L.) Urb var. *pubescens* (DC.) Rudd | - | Aak’ k’an | SH | LDF, MSDF, SV | NA | CA, YU |  | + | + | + |  | + |  |  | + | + | + | + |
| Fabaceae | *Chamaecrista flexuosa* (L.) Greene var. *texana* (Buckley) H. S. Irwin & Barneby | - | - | SH | MG, SV | NA | YU |  |  |  |  |  | + | + | + |  |  |  | + |
| Fabaceae | *Chamaecrista glandulosa* (L.) Greene *var. flavicoma* (Kunth) H. S. Irwin & Barneby | Tamarindillo | Xiiw | SH | EVF, LDF, LIFT, MDF, MSDF, MSTF, SV | NA | CA, QR, YU | + | + | + |  | + | + | + | + | + | + | + | + |
| Fabaceae | *Chamaecrista nictitans* (L.) Moench *var. jaliscencis* (Greenm.) H. S. Irwin & Barneby | - | Ya' ax eek' | SH | CD, EVF, LDF, LIFT, LSDF, MSDF, MSTF, SV | NA | CA, QR, YU | + | + |  |  | + |  | + |  |  | + | + | + |
| Fabaceae | *Chloroleucon mangense* (Jacq.) Britton & Rose var. *leucospermum* | - | Ya’ ax eek’ | SH | LDF, LIFT, MSDF, MSTF | NA | CA, QR, YU |  | + | + | + | + | + | + | + | + | + | + | + |
| Fabaceae | *Clianthus puniceus* (G. Don) Banks & Sol. ex Lindl. | - | Chak muk | SH |  | CU | QR |  |  |  |  |  |  |  |  |  |  |  |  |
| Fabaceae | *Cojoba graciliflora* (S. F. Blake) Britton & Rose | - | Xuul | SH | EVF, MSDF, MSTF | NA | CA, QR | + | + | + | + |  |  | + |  |  | + | + |  |
| Fabaceae | *Dalbergia brownei* (Jacq.) Schinz | - | Aj muk | SH | LIFT, MG, MSDF | NA | CA, QR |  |  | + | + | + |  |  |  |  |  | + | + |
| Fabaceae | *Dalbergia glabra* (Mill.) Standl. | Escobilla | - | SH | EVF, LDF, LIFT, MSTF, SV | NA | CA, QR, YU |  |  |  | + | + | + | + | + | + | + |  |  |
| Fabaceae | *Dalea carthagenensis* (Jacq.) J. F. Macbr. var. *carthagenensis* | Flamboyán | Ch’o’ | HE | LDFCC | NA | QR, YU | + | + |  |  |  |  | + |  |  |  |  | + |
| Fabaceae | *Delonix regia* (Bojer ex Hook.) Raf. | - | - | TR | SV | CU | CA, QR, YU |  |  |  |  | + | + | + | + |  |  |  |  |
| Fabaceae | *Desmodium distortum* (Aubl.) J. F. Macbr. | Cadillo | - | HE | LDF, MSTF, SV | NA | CA, QR, YU | + | + | + |  |  | + | + | + | + | + | + | + |
| Fabaceae | *Desmodium glabrum* (Mill.) DC. | Cadillo | K’iin taj | HE | CD, LDF, MSDF, MSTF, SV | NA | CA, QR, YU | + | + | + |  |  |  |  |  |  | + | + | + |
| Fabaceae | *Desmodium incanum* DC. | Pega pega | K’axab yuuk | HE | CD, EVF, LDF, LIFT, MG, MSDF, MSTF, SAV, SV | NA | CA, QR, YU | + | + | + | + | + | + | + | + | + | + | + | + |
| Fabaceae | *Desmodium procumbens* (Mill.) Hitchc. var. *procumbens* | - | K’iin taj | HE | LDF, MSDF, SV | NA | CA |  |  |  |  |  |  |  | + | + | + | + | + |
| Fabaceae | *Desmodium procumbens* A.S Hichc. var. *typicum* B. G. Schubert | - | - | HE | LDF, MSDF | CU | CA, YU |  | + |  |  |  |  |  |  | + | + | + |  |
| Fabaceae | *Desmodium tortuosum* (Sw.) DC. | Brasilillo | K’iin taj xiiw | HE | CD, LDF, LSDF, MSDF, MSTF, SV | NA | CA, QR, YU | + | + | + | + |  | + | + | + | + | + | + | + |
| Fabaceae | *Diphysa carthagenensis* Jacq. | - | - | SH | LDFCC | NA | YU | + | + | + | + |  | + | + | + |  | + | + |  |
| Fabaceae | *Diphysa yucatanensis* Hanan-Alipi & M. Sousa | Quiebra hacha | Ts’ uts’ uk | TR | LDF, MSDF, LDFCC, MSTF, SV | NA | CA |  | + | + | + | + | + |  |  |  | + | + |  |
| Fabaceae | *Diphysa paucifoliolata* R. Antonio & M. Sousa | - | - | SH | LIFT, SV | NA, EN | CA, QR |  |  |  |  |  |  | + |  |  | + | + | + |
| Fabaceae | *Ebenopsis ebano* (Berland.) Barneby & J.W. Grimes | Ébano | Kaante | TR | CD, LDF, LIFT, MSDF | NA | YU |  |  |  |  |  |  |  | + |  |  |  |  |
| Fabaceae | *Enterolobium cyclocarpum* (Jacq.) Griseb | Conacaste | Piich | TR | LDF, MSDF, SV | NA | CA, QR, YU |  |  | + | + | + |  |  |  |  |  |  |  |
| Fabaceae | *Gliricidia maculata* (Kunth) Walp. | Cacahuananche | Sak ya’ab | TR | LDF, LIFT, MSTF, SV | NA, EN | CA, QR, YU | + | + | + | + | + |  |  |  |  |  |  |  |
| Fabaceae | *Gliricidia sepium* (Jacq.) Kunth ex Walp. | - | - | TR | MSDF | CU | CA, QR, YU |  | + | + | + |  |  |  |  |  |  |  |  |
| Fabaceae | *Haematoxylum campechianum* L. | Palo de campeche | Tooso boon | TR | CDS, LDFCC, LSDF, LIFT, MG, MDF, MSTF | NA | CA, QR, YU | + | + | + |  |  |  |  |  |  |  | + | + |
| Fabaceae | *Harpalyce arborescens* A. Gray | - | - | SH | LDF, SAV | NA | YU | + | + |  |  |  |  |  |  |  |  |  | + |
| Fabaceae | *Havardia albicans* (Kunth) Britton & Rose | - | -  Chukum | TR | CD, LDF, LIFT, MG, MSTF, SAV | NA | CA, QR, YU |  |  | + | + | + | + | + | + | + | + | + |  |
| Fabaceae | *Havardia pallens* (Benth.) Britton & Rose | - | - | TR | LDF, LDFCC | NA | YU |  |  | + | + | + | + | + | + | + | + | + |  |
| Fabaceae | *Indigofera lespedezioides* Kunth | - | - | HE | MSDF, SAV, SV | NA | CA | + | + |  | + | + | + | + | + | + | + | + | + |
| Fabaceae | *Indigofera suffruticosa* Mill. | Añil | Ch’oj xiiw | SH | CD, SV | NA | CA, QR, YU | + | + | + | + | + | + | + | + | + | + | + | + |
| Fabaceae | *Indigofera trita* L. f. subsp. *scabra* (Roth) de Kort & G. Thijsse | - | - | SH | CD, SV | NA | CA, YU | + | + | + |  |  | + | + | + | + | + | + | + |
| Fabaceae | *Indigofera hirsuta* L. | - | - | SH | SV | NT | QR, YU |  |  |  | + |  |  |  |  | + | + | + |  |
| Fabaceae | *Leucaena leucocephala* (Lam.) de Wit subsp. *leucocephala* | - | - | TR | LDF, LDFCC, LIFT, MSTF, SV | NA | CA, QR, YU | + | + | + | + | + | + | + | + | + | + | + | + |
| Fabaceae | *Lonchocarpus castilloi* Standl. | Corazón azul | Baal che’ | TR | MSTF, MSDF, SV | NA, EN | CA, QR |  |  |  |  |  |  | + |  |  | + |  | + |
| Fabaceae | *Lonchocarpus guatemalensis* Benth. | Palo gusano | K’an xu’ul | TR | CD, EVF, LDF, MSTF | NA | CA, QR, YU |  |  |  | + | + | + | + | + | + |  |  |  |
| Fabaceae | *Lonchocarpus hondurensis* Benth. | - | - | TR | EVF, MSTF | NA | CA, QR |  | + | + | + | + | + |  |  |  |  |  |  |
| Fabaceae | *Lonchocarpus luteomaculatus* Pittier | - | - | TR | EVF, LDF, MSDF, MSTF | NA | CA |  |  |  |  | + | + | + | + |  |  |  |  |
| Fabaceae | *Lonchocarpus punctatus* Kunth | Mata buey | Baal che’ | TR | LDF, MSDF, MSTF | NA | CA, QR, YU |  |  |  |  |  |  |  |  |  | + | + |  |
| Fabaceae | *Lonchocarpus rugosus* Benth. | - | K’anasín | TR | LDF, LIFT, MSDF, MSTF | NA | CA, QR, YU | + | + |  |  | + |  |  | + | + | + | + | + |
| Fabaceae | *Lonchocarpus xuul* Lundell | Palo gusano | - | TR | LDF, LTF, MSDF, MSTF, SV | NA, EN | CA, QR, YU |  |  | + | + | + | + |  |  |  |  |  | + |
| Fabaceae | *Lonchocarpus yucatanensis* Pittier | Cicahuite | K’an xu’ul | TR | LSDF, MSDF, MSTF | NA, EN | CA, QR, YU |  |  |  |  |  | + | + | + | + | + |  |  |
| Fabaceae | *Lysiloma acapulcense* (Kunth) Benth. | - | - | TR | LDF, MSTF | NA | CA, YU | + |  |  | + | + |  |  | + | + | + |  | + |
| Fabaceae | *Lysiloma latisiliquum* (L.) Benth. | - | - | TR | LDF, MSDF, MSTF, SAV, SV | NA | CA, QR, YU |  |  | + | + | + | + | + | + | + | + | + |  |
| Fabaceae | *Machaerium cirrhiferum* Pittier | - | - | TR | EVF | NA | CA, QR |  |  | + | + | + |  | + |  |  |  |  |  |
| Fabaceae | *Machaerium falciforme* Rudd | - | - | WC | AV, MG | NA | CA |  |  |  | + | + |  |  |  |  |  |  |  |
| Fabaceae | *Machaerium riparium* Brandegee | - | - | TR | MSTF | NA | CA, QR | + | + | + |  | + |  |  |  |  |  |  | + |
| Fabaceae | *Machaerium seemannii* Benth. ex Seem. | - | - | SH | MSTF | NA | CA, QR |  |  |  |  |  | + | + | + |  |  |  |  |
| Fabaceae | *Mariosousa dolichostachya* (S. F. Blake) Seigler & Ebinger | - | - | TR | CD, LDF, SV | NA, EN | CA, QR, YU |  |  |  |  | + | + |  |  |  |  | + | + |
| Fabaceae | *Mimosa albida* Humb. & Bonpl. ex Willd. var. *albida* | Dormilona macho | - | SH | LDF, MSTF, SV | NA | CA, YU | + | + |  |  | + |  |  |  |  | + |  | + |
| Fabaceae | *Mimosa albida* Humb. & Willd. var. *strigosa* (Willd.) B. L. Rob. | - | - | SH | LDF, MSDF, SV | NA | CA, YU |  |  | + |  |  |  |  |  | + | + | + |  |
| Fabaceae | *Mimosa bahamensis* Benth. | - | Sak káatsim | SH | LDF, LDFCC, SV | NA | CA, QR, YU | + |  |  | + | + | + | + | + | + | + | + |  |
| Fabaceae | *Mimosa pigra* L. var. *berlandieri* (A. Gray ex Torr.) B. L. Turner | - | Je’ beech | SH | LDF, LIFT, MSTF | NA | CA, QR, YU | + |  |  |  | + | + | + |  | + | + |  |  |
| Fabaceae | *Mimosa pigra* L. var. *pigra* | Zarza | Je’ beech | SH | MG, MSDF, SV | NA | CA, QR, YU | + | + |  |  | + |  |  | + |  | + |  |  |
| Fabaceae | *Mimosa pudica* L. | Dormilona | Múuts’il xiiw | HE | LDF, SV | NA | QR | + | + |  |  | + |  |  |  |  | + |  |  |
| Fabaceae | *Mimosa somnians* Humb. & Bonpl. ex Willd. | Pica pica | - | SH | MSDF, SV | NA | CA |  | + |  |  |  |  |  |  |  | + |  |  |
| Fabaceae | *Mucuna pruriens* (L.) DC. | - | Bu’ul | HC | LDF, SV | NA | CA, QR, YU |  |  |  |  |  |  |  |  |  | + | + | + |
| Fabaceae | *Nissolia fruticosa* Jacq. var. *fruticosa.* | - | K’ant’u’ul | HC | LDF, MG, MSDF, SV | NA | CA, QR, YU | + | + | + |  |  |  | + | + | + | + | + | + |
| Fabaceae | *Oxyrhynchus volubilis* Brandegee | Ojo de venado | - | HC | LSDF | NA | CA, QR, YU |  |  | + | + | + |  |  |  |  | + |  |  |
| Fabaceae | *Phaseolus lunatus* L. | Frijol ib | Ib | HC | SV | NT | CA, QR, YU |  |  |  |  |  | + |  |  |  |  |  |  |
| Fabaceae | *Phaseolus vulgaris* L. | Huamuchil | - | HC | SV | CU | CA, QR, YU | + | + |  |  | + | + | + | + |  |  |  | + |
| Fabaceae | *Piscidia piscipula* (L.) Sarg. | Guamúchil | Ja'Abin | TR | LDF, LDFCC, LIFT, MSTF | NA | CA, QR, YU | + |  | + | + |  |  |  |  |  |  |  |  |
| Fabaceae | *Pithecellobium dulce* (Roxb.) Benth. | - | - | SH | CD, LDF, MSDF, SV | NA | CA, QR, YU |  | + | + | + | + | + |  |  |  |  |  |  |
| Fabaceae | *Pithecellobium lanceolatum* (Humb. & Bonpl. ex Willd.) Benth. | - | - | TR | LDF, LIFT, MG, MSDF, MSTF | NA | CA, QR, YU | + | + | + | + | + |  | + |  | + | + | + |  |
| Fabaceae | *Pithecellobium winzerlingii* Britton & Rose | - | - | SH | MG, MSTF | NA | CA, QR | + | + | + |  |  |  | + |  |  | + | + | + |
| Fabaceae | *Platymiscium yucatanum* Standl. | Granadillo | Subin che’ | TR | LDF, MSDF, MSTF, SV | NA | CA |  | + | + | + | + | + |  |  |  |  |  |  |
| Fabaceae | *Prosopis juliflora* (Sw.) DC. var. *juliflora* | - | - | TR | AV, LDF, LDFCC | NA | YU |  |  | + | + |  | + |  |  | + |  |  |  |
| Fabaceae | *Samanea saman* (Jacq.) Merr. | - | - | TR | RV, SV | CU | CA, QR, YU |  |  | + | + | + |  |  |  | + |  |  |  |
| Fabaceae | *Senegalia gaumeri* (S. F. Blake) Britton & Rose | - | Boox káatsim | TR | CDS, LDF, MSTF, SV | NA, EN | CA, QR, YU |  |  |  | + | + | + | + | + |  |  | + | + |
| Fabaceae | *Senegalia hayesii* (Benth.) Britton & Rose | Angico branco | - | TR | LSDF | NA | CA |  |  |  |  |  |  |  |  |  | + |  |  |
| Fabaceae | *Senegalia polyphylla* (DC.) Britton | - | - | TR | LIFT, MSDF, MSTF | NA | CA, QR |  |  |  |  |  |  |  |  | + | + | + |  |
| Fabaceae | *Senegalia riparia* (Kunth) Britton | - | Káatsim | SH | LDF, MSDF, SV | NA | CA, QR, YU |  |  |  |  |  | + | + | + | + |  |  |  |
| Fabaceae | *Senna alata* (L.) Roxb. | Habilla prieta | - | TR | SV | NA | CA, QR, YU | + | + | + | + |  |  |  |  |  |  | + | + |
| Fabaceae | *Senna atomaria* (L.) H. S. Irwin & Barneby | Bicho | - | TR | SV | NA | QR, YU |  |  |  | + | + | + | + | + | + | + | + | + |
| Fabaceae | *Senna occidentalis* (L.) Link | Frijolillo | Bu’ulk’aax | SH | EVF, SV | NA | CA, QR, YU |  | + | + | + | + | + | + | + |  | + | + | + |
| Fabaceae | *Senna pallida* (Vahl) H. S. Irwin & Barneby var. *gaumeri* (Britton & Rose) H. S. Irwin & Barneby | Zorrillo | Ch’iilibmich | SH | LDF, LDFCC, SV | NA | CA, QR, YU | + |  | + | + | + | + | + |  | + | + | + | + |
| Fabaceae | *Senna peralteana* (Kunth) H. S. Irwin & Barneby | - | Béeb | SH | EVF, LIFT, LDF, MSDF, MSTF, SV | NA | CA, QR, YU |  |  | + | + | + |  | + |  | + |  |  |  |
| Fabaceae | *Senna racemosa* (Mill.) H. S. Irwin & Barneby var. *racemose* | - | - | TR | EVF, LDF, MSDF, MSTF, SV | NA | CA, QR, YU | + | + | + | + |  | + |  |  |  | + | + | + |
| Fabaceae | *Senna reticulata* (Willd.) H. S. Irwin & Barneby | - | - | TR | LDF, SV | NA | CA | + | + | + |  |  |  |  |  | + | + | + | + |
| Fabaceae | *Senna undulata* (Benth.) H. S. Irwin & Barneby | Quelitillo | K’an chik’in aak | TR | EVF, LIFT, SAV | NA | CA, QR, YU | + |  |  |  |  |  |  |  | + | + | + | + |
| Fabaceae | *Senna uniflora* (Mill.) H. S. Irwin & Barneby | Frijolillo | Bu’ulch’o’ k’aax | SH | EVF, LDF, LSDF, LIFT, MDF, MSDF, MSTF, SAV, SV | NA | CA, QR, YU | + |  |  |  |  | + | + | + | + | + | + | + |
| Fabaceae | *Senna villosa* (Mill.) H. S. Irwin & Barneby | - | Saal che | SH | EVF, LDF, LIFT, MDF, MSDF, MSTF, SV | NA | CA, QR, YU |  | + | + |  |  | + |  |  | + | + | + | + |
| Fabaceae | *Sesbania herbacea* (Mill.) McVaugh | - | Muk | HE | CD, LDF, LIFT, MSTF, SV | NA | CA, QR, YU | + |  |  |  |  |  |  |  | + | + |  |  |
| Fabaceae | *Sphinga platyloba* (Bertero ex CD.) Barneby & J.W. Grimes | - | - | TR | CD, EVF, LDF, LIFT, MG, MSDF, MSTF | NA | CA, QR, YU |  |  | + | + | + | + | + | + | + | + | + |  |
| Fabaceae | *Swartzia cubensis* (Britton & P.Wilson) Standl. var. *cubensis* | Corazón azul | K’aatal oox | TR | MSTF, SV | NA | CA, QR, YU |  | + | + | + | + | + |  |  |  |  |  |  |
| Fabaceae | *Tamarindus indica* L. | Tamarindo | - | TR | LDF, SV | NA | CA, QR, YU |  |  |  |  |  | + |  |  | + | + | + |  |
| Fabaceae | *Tephrosia cinerea* (L.) Pers. | Mañanitas | Bu’ul beech | HE | SV | NA | CA, QR, YU |  | + | + |  | + | + | + |  | + | + | + |  |
| Fabaceae | *Vachellia × cedilloi* (L. Rico) Seigler & Ebinger | - | - | SH | MSTF | NA | QR |  |  |  |  |  | + |  |  |  |  |  |  |
| Fabaceae | *Vachellia collinsii* (Saff.) Seigler & Ebinger | - | Subin | SH | EVF, LDF, LIFT, MG, MSTF, SV | NA | CA, QR, YU | + | + | + | + | + | + | + |  |  | + | + | + |
| Fabaceae | *Vachellia farnesiana* (L.) Wight & Arn. var. *farnesiana* | - | - | TR | SV | SR | CA, QR, YU | + | + | + | + | + | + | + | + | + | + | + | + |
| Fabaceae | *Vachellia gentlei* (Standl.) Seigler & Ebinger | - | Subin | TR | EVF, MSTF | NA, EN | CA, QR |  |  | + | + | + |  |  |  |  |  |  |  |
| Fabaceae | *Vachellia globulifera* (Saff.) Seigler & Ebinger | Carnezuelo blanco | Subin | SH | LDF, MSTF, SV | NA | CA, QR, YU |  |  | + |  | + |  |  |  |  |  | + |  |
| Fabaceae | *Vachellia pennatula* var. *parvicephala* (Seigler & Ebinger) Seigler & Ebinger | - | - | TR | LDF, LSDF, SV | NA | CA, YU |  |  |  |  |  |  |  |  |  |  |  |  |
| Fabaceae | *Vachellia pringlei* (Rose) Seigler & Ebinger | - | - | TR | LDF, LDFCC, LIFT, MG, MSTF | NA | YU |  | + | + |  |  |  |  |  |  |  |  |  |
| Fabaceae | *Vachellia cornigera* (L.) Seigler & Ebinger | - | Subin | SH | EVF, LDF, MSDF, MSTF | NA | CA, QR, YU |  |  |  | + | + | + | + | + |  | + |  |  |
| Fabaceae | *Vigna unguiculata* (L.) Walp. | - | - | HC | LDF, SV | CU | CA, QR, YU |  |  | + | + |  |  |  | + | + |  | + |  |
| Fabaceae | *Zygia recordii* Britton & Rose | - | - | TR | LIFT, MSTF | NA | CA, QR |  |  | + | + | + | + | + | + |  |  |  |  |
| Fabaceae | *Zygia stevensonii* (Standl.) Killip ex Record | - | - | TR | EVF, LDF, MSTF, SV | NA | CA, QR |  |  | + |  |  | + | + | + |  |  | + | + |
| Goodeniaceae | *Scaevola plumieri* (L.) Vahl | - | Chunup | SH | CD, LIFT, MG | NA | CA, QR, YU |  | + | + | + | + |  | + |  |  | + |  | + |
| Hernandiaceae | *Gyrocarpus jatrophifolius* Domin. | - | Ki´is,kiste’ | TR | MG | NA | CA, QR, YU |  |  |  |  | + |  | + | + |  |  |  | + |
| Hydroleaceae | *Hydrolea spinosa* L. | Huevos de toro | Púuts’ mukuy | SH | LTF, LIFT, MSTF, AV, SV | NA | CA, QR, YU |  |  |  |  | + |  | + | + | + | + | + | + |
| Lamiaceae | *Callicarpa acuminata* Kunth | - | Kú uk k’iin | SH | EVF, LDF, MSDF, MSTF, SV | NA | CA, QR, YU |  | + | + | + | + | + |  |  |  |  |  |  |
| Lamiaceae | *Cantinoa mutabilis* (Rich.) Harley & J. F. B. Pastore | - | - | HC | SV | NA | CA, QR, YU | + | + | + |  |  |  |  |  | + | + | + | + |
| Lamiaceae | *Clinopodium ludens* (Shinners) A. Pool | Poleo | - | HE | EVF, SV | CU | CA, YU |  |  |  |  | + |  | + |  |  |  | + |  |
| Lamiaceae | *Cornutia pyramidata* L. | Bastón de vieja | Loob che’ | TR | LDF, LDFCC, LIFT, MSDF, MSTF, SV | NA | CA, QR, YU | + | + | + | + | + | + | + | + |  |  |  |  |
| Lamiaceae | *Hyptis capitata* Jacq. | Cabezona | - | HE | MSDF, MSTF, SV | NA | CA, QR | + | + | + |  |  |  |  |  |  | + | + | + |
| Lamiaceae | *Hyptis pectinata* (L.) Poit. | Barretero | Xóolte’ | SH | LDF, MSDF, MSTF, SV | NA | CA, QR, YU | + | + | + | + | + | + |  |  |  | + | + | + |
| Lamiaceae | *Hyptis suaveolens* (L.) Poit. | Hierba martín | - | HE | LDF, LDFCC, LIFT, MSDF, MSTF, SV | NA | CA, QR, YU | + | + | + |  |  |  |  |  | + | + | + | + |
| Lamiaceae | *Hyptis verticillata* Jacq. | Epazotillo | Xnuuk | HE | LDF, MSDF, MSTF, SV | NA | CA, QR, YU | + | + |  |  | + | + | + | + | + | + | + | + |
| Lamiaceae | *Leonotis nepetifolia* (L.) R. Br. | Castillo | Jaras xiiw | HE | CDS, LDF, LDFCC, LIFT, MSDF, MSTF, SV | NA | CA, QR, YU | + | + |  |  |  |  | + | + | + | + | + | + |
| Lamiaceae | *Leonurus japonicus* Houtt | Cola de león | - | HE | SV | NA | QR, YU | + | + |  |  |  |  | + | + | + | + | + | + |
| Lamiaceae | *Ocimum basilicum* L. | Albahaca | - | HE | SV | CU | CA, QR, YU |  |  |  |  |  |  |  | + | + | + | + |  |
| Lamiaceae | *Ocimum campechianum* Mill. | Albahaca de monte | X kakaltuum | HE | CDS, LDF, LDFCC, LIFT, MSDF, MSTF, SV | NA | CA, QR, YU | + | + |  |  |  |  | + | + | + | + | + | + |
| Lamiaceae | *Salvia coccinea* Buc’hoz ex Etl. | Mirto | - | HE | CDS, LDF, LIFT, MSDF, MSTF, SV | NA | CA, QR, YU | + | + | + | + | + | + | + | + | + | + | + | + |
| Lamiaceae | *Salvia serotina* L. | Hierba santa | K’aaj xiiw | HE | MSDF, MSTF, SV | NA | CA, QR, YU |  |  |  |  | + | + | + | + | + |  |  |  |
| Lamiaceae | *Tectona grandis* L. f. | Teca | - | TR | SV | CU | CA, QR | + | + |  |  |  |  |  |  | + | + |  |  |
| Lamiaceae | *Teucrium vesicarium* Mill. | - | - | HE | LDF, MSDF, SV | NA | CA, QR, YU | + | + | + | + | + | + | + | + | + | + | + | + |
| Lamiaceae | *Vitex gaumeri* Greenm. | - | Ya’Axnik | SH | LDF, LIFT, MSDF, MSTF | NA | CA, QR, YU |  |  |  |  |  | + | + |  |  |  |  |  |
| Lamiaceae | *Vitex trifolia* L. | Norchila de la india | - | SH | LTF, SV | CU | CA, QR, YU |  |  |  |  |  |  |  |  | + | + | + |  |
| Lauraceae | *Nectandra coriacea* (Sw.) Griseb. | Laurel | Sip che’ | TR | EVF, MSTF, SV | NA | CA, QR, YU |  | + | + | + | + | + | + |  |  |  |  |  |
| Lauraceae | *Nectandra salicifolia* (Kunth) Nees | Laurelillo | Jobon ka’aax | TR | EVF, LDF, MSTF, SV | NA | CA, QR, YU | + | + | + | + | + |  |  |  |  |  |  |  |
| Lauraceae | *Persea americana* Mill. | - | - | TR | SV | CU | CA, QR, YU |  |  |  |  | + |  |  |  |  | + |  |  |
| Liliaceae | *Nothoscordum inodorum* (Aiton) Asch. & Graebn. | - | - | HE | SV | CU | CA, YU |  |  |  | + |  | + |  |  |  |  |  |  |
| Loasaceae | *Gronovia scandens* L. | Ortiga | Láal muuch | HE | LDF, MSDF, MSTF | NA | CA, QR, YU | + |  | + |  | + | + | + | + | + | + | + |  |
| Loganiaceae | *Strychnos brachistantha* Standl. | - | - | WC | EVF, SV | NA | QR |  |  |  |  |  |  | + | + | + |  |  |  |
| Loganiaceae | *Strychnos panamensis* Seem. | - | - | WC | LTF, MSTF | NA | QR | + |  |  | + | + | + |  |  |  |  |  |  |
| Lythraceae | *Ammannia coccinea* Rottb. | - | - | HE | AV, LDF, LDFCC, MG, SAV | NA | CA, QR, YU | + |  | + |  | + |  | + |  |  |  | + |  |
| Lythraceae | *Cuphea carthagenensis* (Jacq.) J. F. Macbr. | - | - | HE | LDF, LIFT | NA | YU | + | + |  |  | + |  | + |  | + |  |  |  |
| Lythraceae | *Cuphea gaumeri* Koehne | - | - | HE | AV, LDF, MG, MSDF, SV | NA, EN | CA, QR, YU | + | + |  |  | + | + | + | + | + | + | + | + |
| Lythraceae | *Cuphea utriculosa* Koehne | - | - | SH | LIFT, SV | NA | CA, QR | + | + |  | + | + |  | + | + |  |  | + | + |
| Lythraceae | *Lagerstroemia indica* L. | - | - | SH | SV | CU | CA, QR | + |  |  |  |  | + | + | + |  |  |  |  |
| Lythraceae | *Lawsonia inermis* L. | - | - | SH | LDF, SV | CU | CA, QR, YU |  |  |  |  |  | + |  | + |  |  |  |  |
| Lythraceae | *Rotala ramosior* (L.) Koehne. | - | - | SH | AV, LIFT, SV | NA | CA, QR | + | + | + | + | + | + | + | + | + | + | + | + |
| Malpighiaceae | *Adelphia hiraea* (Gaertn.) W. R. Anderson | Hiraea adelphia | - | WC | MSDF, MSTF | NA | QR |  |  |  | + | + | + | + |  |  |  |  |  |
| Malpighiaceae | *Bunchosia swartziana* Griseb. | Cojón de fraile | Sip che’ | SH | LDF, MSDF, SV | NA | CA, QR, YU |  |  | + | + | + | + | + | + | + | + | + |  |
| Malpighiaceae | *Byrsonima bucidifolia* Standl. | - | - | TR | LIFT, MSDF, MSTF | NA | CA, QR, YU |  | + |  |  |  |  | + | + | + |  |  |  |
| Malpighiaceae | *Byrsonima crassifolia* (L.) Kunth | Nance | - | TR | MSTF, SAV | NA | CA, QR, YU | + |  |  |  | + | + | + |  |  |  | + | + |
| Malpighiaceae | *Carolus sinemariensis* (Aubl.) W. | - | - | WC | LIFT, MSTF | NA | CA, QR, YU |  |  |  | + | + | + | + | + | + |  |  |  |
| Malpighiaceae | *Gaudichaudia albida* Schltdl. & Cham. | - | Aak’ | WC | LDF, LIFT, MG, MSDF | NA | CA, YU |  |  |  |  |  |  |  |  |  | + | + | + |
| Malpighiaceae | *Heteropterys brachiata* (L.) DC. | Cafecillo | Soj aak’ | SR | MSDF, MSTF, LDF, SV | NA | CA, QR, YU |  |  |  |  |  |  | + | + | + | + | + |  |
| Malpighiaceae | *Heteropterys laurifolia* (L.) A. Juss. | Chilillo | Péem aak’ | WC | EVF, MSDF | NA | CA, QR | + | + | + | + | + | + |  |  |  |  |  |  |
| Malpighiaceae | *Heteropterys lindeniana* A. Juss. | - | - | WC | LDF, LIFT, MSDF, MSTF | NA | CA, QR, YU |  |  |  |  |  |  | + | + | + | + | + |  |
| Malpighiaceae | *Malpighia glabra* L. | Nance | Wayate’ | SH | EVF, LDF, MSDF, MSTF | NA | CA, QR, YU | + | + | + | + | + | + | + | + | + | + | + | + |
| Malpighiaceae | *Malpighia lundellii* C. V. Morton | - | Wayate’ | TR | LDF, MG, MSDF, MSTF | NA | CA, QR, YU |  |  |  |  | + |  | + |  | + | + | + | + |
| Malpighiaceae | *Psychopterys rivularis* (C. V. Morton & Standl.) W. R. Anderson & S. Corso | - | - | WC | EVF, MSTF | NA | QR | + | + | + | + |  |  |  |  |  |  |  |  |
| Malpighiaceae | *Stigmaphyllon ellipticum* (Kunth) A. Juss. | Contrahierba macho | Tíip’te’ aak’ | WC | LDF, MSDF, MSTF, SV | NA | CA, QR, YU | + | + | + | + | + | + | + | + | + | + | + | + |
| Malpighiaceae | *Stigmaphyllon lindenianum* A. Juss. | - | - | WC | LDF, LIFT, MSDF, MSTF, SV | NA | CA, QR, YU | + | + | + | + | + | + | + | + | + | + | + | + |
| Malpighiaceae | *Tetrapterys schiedeana* Schltdl. & Cham | - | Sak aak’ | WC | EVF, MSTF, MSDF | NA | CA, QR, YU |  |  | + | + | + |  |  |  |  |  |  | + |
| Malpighiaceae | *Tetrapterys seleriana* Nied. | - | Péepem aak’ | WC | LDF, LIFT, MSDF, SV | NA | CA, QR, YU |  |  | + | + | + | + | + |  | + |  |  |  |
| Malvaceae | *Abelmoschus esculentus* (L.) Moench | Quimbombó | - | SH | EVF, SV | CU | YU | + |  |  |  | + |  |  |  |  | + | + | + |
| Malvaceae | *Abutilon permolle* (Willd.) Sweet | - | Sak xiiw | HE | CDS, LDF, LDFCC, MSDF, MSTF, SV | NA | CA, QR, YU | + | + | + | + | + | + |  |  |  |  |  | + |
| Malvaceae | *Abutilon trisulcatum* (Jacq.) Urb. | - | Sak le’xiiw | HE | LDF, LIFT, MSDF, MSTF | NA | CA, QR, YU | + | + | + | + | + | + | + | + | + | + | + | + |
| Malvaceae | *Anoda acerifolia* Cav. | Violeta del campo | - | HE | LIFT, SV | NA | CA, YU |  |  |  |  |  |  |  |  | + | + | + | + |
| Malvaceae | *Anoda cristata* (L.) Schlecht. | Amapolita | Tsáayal tasai | HE | LDF, MSDF, MSTF, SV | NA | CA, QR, YU | + | + | + | + | + | + | + | + | + | + | + | + |
| Malvaceae | *Ayenia abutilifolia* (Turcz.) Turcz | - | Piix t’oon | HE | EVF, LDF, MSTF | NA | QR, YU |  |  |  |  |  |  | + | + | + | + | + | + |
| Malvaceae | *Ayenia ardua* Cristóbal | - | Piix t’oon ch’iich | SH | LDF, MSDF, SV | NA | QR, YU |  |  | + | + | + | + | + | + | + | + | + |  |
| Malvaceae | *Bakeridesia gaumeri* (Standl.) D. M. Bates | - | Jóol | SH | LDF, MSDF, MSTF, LDFCC, SV | NA | QR | + | + | + | + | + | + | + | + | + | + | + | + |
| Malvaceae | *Bakeridesia yucatana* (Standl.) D. M. Bates | - | - | SH | MSDF, MSTF | NA, EN | CA, QR, YU |  |  | + | + | + | + |  |  |  |  |  |  |
| Malvaceae | *Bastardia viscosa* (L.) Kunth | - | Sak le’a sak miis | SH | CD, LDF, MSDF | NA | CA, QR, YU | + | + | + | + | + | + | + | + | + | + | + | + |
| Malvaceae | *Byttneria aculeata* (Jacq.) Jacq. | Uña de gato | K’iil ix | HE | EVF, LDF, LIFT, MSDF | NA | CA, QR, YU | + | + | + |  |  |  |  | + | + | + | + | + |
| Malvaceae | *Ceiba aesculifolia* (Kunth) Britt. & Baker f. | - | - | TR | LDF | NA | CA, QR, YU |  |  | + | + | + |  | + |  |  |  | + |  |
| Malvaceae | *Ceiba pentandra* (L.) Gaertn. | Pochote | Yax che | TR | MSTF | NA | YU | + | + |  | + | + | + |  |  |  |  |  | + |
| Malvaceae | *Cienfuegosia yucatanensis* Millsp. | - | - | HE | LDF, LDFCC | NA | YU |  | + | + | + | + | + | + | + | + | + | + |  |
| Malvaceae | *Corchorus hirtus* L. | - | Chi’chi’ bej | HE | EVF, LDF | NA | CA, QR, YU |  |  | + | + | + | + | + | + | + |  |  |  |
| Malvaceae | *Corchorus siliquosus* L. | Malvavisco | Niich’ yuuk | SH | EVF, LDF, MSDF, MSTF, SV | NA | QR, YU | + | + |  |  |  | + |  | + | + | + | + | + |
| Malvaceae | *Gaya calyptrata* (Cav.) Kunth ex K. Schum. | - | Sak xiiw | HE | LDF, MSDF, SV | NA | YU | + | + | + | + | + | + | + | + | + | + | + | + |
| Malvaceae | *Gossypium barbadense* L. | Algodón egipcio | - | SH | CD, LDF | CU | CA, QR, YU |  |  | + |  | + | + |  |  |  |  | + |  |
| Malvaceae | *Gossypium hirsutum* L. | Algodón mexicano | Taman | SH | CDS, LDF, LDFCC, LIFT, SV | NT | CA, QR, YU | + | + | + | + | + | + | + | + | + | + | + | + |
| Malvaceae | *Guazuma ulmifolia* Lam. | Guasmo | Kabal Pixoy o poxoy | TR | LDF, SV | NA | CA, QR, YU |  | + |  | + | + | + | + |  | + | + | + | + |
| Malvaceae | *Hampea trilobata* Standl. | Majagua | Jóol, k’an jóol | SH | LSDF, LDF, MSTF | NA, EN | CA, QR, YU | + | + | + | + | + | + | + | + | + | + | + | + |
| Malvaceae | *Helicteres baruensis* Jacq. | - | Sutup | SH | EVF, LDF, LIFT, MSTF | NA | CA | + | + | + | + | + | + | + | + | + | + | + | + |
| Malvaceae | *Helicteres guazumifolia* Kunth | - | Coralillo | SH | LDF, MSTF, SV | NA | CA, QR, YU |  |  |  | + | + | + | + | + |  |  |  |  |
| Malvaceae | *Heliocarpus donnellsmithii* Rose | Adán | Joolol | TR | LDF, LIFT, MSDF, MSTF | NA | CA, QR, YU | + | + |  |  |  |  |  |  |  | + | + | + |
| Malvaceae | *Heliocarpus mexicanus* (Turcz.) Sprague | - | - | TR | LDF, MSDF, MSTF, SV | NA | CA, QR, YU | + | + | + | + | + | + | + |  |  | + | + | + |
| Malvaceae | *Herissantia crispa* (L.) Brizicky | Monancillo blanco | P’up’ul iik’ | HE | LDF, LIFT, MSDF, MSTF, SV | NA | CA, QR, YU | + | + | + | + | + | + | + | + | + | + | + | + |
| Malvaceae | *Hibiscus clypeatus* L. | Angú | U kúuch xiiw | SH | LDF, MSDF, MSTF, SV | NA | CA, QR, YU | + |  |  |  |  |  |  |  |  | + | + | + |
| Malvaceae | *Hibiscus rosa-sinensis* L. | Amapola | - | SH | SV | CU | CA, QR, YU |  |  |  |  |  |  | + | + | + | + |  |  |
| Malvaceae | *Luehea candida* (Moc. & Sessé ex CD.) Mart. | Patashté | K’askáat | TR | LDF, MSDF, MSTF | NA | CA, QR, YU |  |  | + | + | + | + | + | + | + |  |  |  |
| Malvaceae | *Luehea speciosa* Willd. | - | K’an kaat | TR | EVF, LDF, LIFT, MSTF, MSDF, MDF | NA | CA, QR, YU | + | + | + | + | + | + | + |  |  | + | + | + |
| Malvaceae | *Malachra capitata* (L.) L. | Malva | Malva xiw | HE | AV, LDF, LIFT, SV | NA | CA, QR, YU | + | + | + | + | + | + | + | + | + | + | + | + |
| Malvaceae | *Malachra fasciata* Jacq. | - | - | HE | AV, LDF, LIFT, SV | NA | YU | + | + | + | + | + | + | + | + | + | + | + | + |
| Malvaceae | *Malvastrum americanum* (L.) Torr. | - | - | SH | LDF, SV | NA | CA, QR, YU | + | + | + | + | + | + | + | + | + | + | + | + |
| Malvaceae | *Malvastrum coromandelianum* (L.) Garcke | Malva colorada | Malva xiw | HE | CD, CDS, LDF, MSDF, MSTF, SV | NA | CA, QR, YU | + | + | + | + | + | + | + | + | + | + | + | + |
| Malvaceae | *Malvaviscus arboreus* Cav. | Manzanilla | Taman che’ | SH | CDS, LDF, LDFCC, LIFT, MSDF, MSTF, SV | NA | CA, QR, YU | + | + | + | + | + | + | + | + | + | + | + | + |
| Malvaceae | *Melochia nodiflora* Sw. | - | Kabal pixoy | HE | LDF, MSTF | NA | CA, QR, YU | + | + | + | + | + | + | + | + |  |  | + | + |
| Malvaceae | *Melochia pyramidata* L. | - | Chi’chi’ bej | HE | LDF, MSTF, SV | NA | CA, QR, YU | + | + | + | + | + | + | + | + |  |  |  |  |
| Malvaceae | *Melochia tomentosa* L. | - | Zak chi’chi’bej | SH | CD, MG, LDF, LIFT, MSTF, SV | NA | CA, QR, YU | + | + | + | + | + | + | + | + | + | + | + | + |
| Malvaceae | *Pachira aquatica* Aubl. | Amapola | K’uuy che | TR | MG, RV | NA | CA, QR, YU | + | + | + | + | + | + | + | + | + | + | + | + |
| Malvaceae | *Pseudabutilon umbellatum* (L.) Fryxell | - | Ak le’ | SH | CDS, LDF, LDFCC, MSDF, MSTF, SV | NA | CA, QR, YU |  |  |  |  |  |  |  |  | + | + | + | + |
| Malvaceae | *Pseudobombax ellipticum* (Kunth) Dugand | Amapola | Chak k’uuyche | TR | MSTF, SA | NA | CA, YU | + | + | + | + |  |  |  |  |  |  |  |  |
| Malvaceae | *Sida abutifolia* Mill. | - | W’aay xiiw | HE | LDF, LDFCC, MSTF | NA | CA, QR, YU | + | + | + | + | + | + | + | + | + | + | + | + |
| Malvaceae | *Sida acuta* Burm. f. | - | Chi’chi’ bej | HE | LDF, EVF, MSDF, MSTF, SV | NA | CA, QR, YU | + | + | + | + | + | + | + | + | + | + | + | + |
| Malvaceae | *Sida cordifolia* L. | Malva | Sak le’ | SH | MSTF, LDF, SV | NA | CA, YU | + | + | + | + | + | + | + | + | + | + | + | + |
| Malvaceae | *Sida linifolia* Cav. | - | - | HE | LDF, LIFT, SAV | NA | CA | + | + | + | + | + | + | + | + | + | + | + | + |
| Malvaceae | *Sida spinosa* L. | - | Chi’chi’bej | SH | LDF, MSDF, SAV, SV | NA | CA, QR, YU | + | + | + | + | + | + | + | + | + | + | + | + |
| Malvaceae | *Trichospermum grewiifolium* (A. Rich.) Kosterm. | - | - | TR | LDF, MSDF, MSTF, SV | NA | CA, QR | + | + | + | + |  |  |  |  |  |  |  |  |
| Malvaceae | *Triumfetta bogotensis* DC. | - | Ooch mul | SH | LDF, MSDF, MSTF, SV | NA | QR, YU | + | + | + | + |  |  |  |  |  |  | + | + |
| Malvaceae | *Triumfetta semitriloba* Jacq. | Cadillo | Ooch mul | SH | EVF, LDF, MSDF, SV | NA | CA, QR, YU | + | + | + | + |  |  |  |  |  |  | + |  |
| Malvaceae | *Turnera odorata* Rich. | - | - | SH | EVF, MSTF, SV | NA | CA, QR, YU | + |  |  |  | + |  |  |  |  |  |  |  |
| Malvaceae | *Waltheria indica* L. | Malva del monte | Sak mis bil | HE | CD, LDF, LIFT, MG, MSTF, SV | NA | CA, QR, YU | + | + | + | + | + | + | + | + | + | + | + | + |
| Malvaceae | *Waltheria rotundifolia* Schrank | - | - | HE | CD, CDS, SV | NA | CA, YU |  | + | + |  |  |  |  |  |  |  |  |  |
| Malvaceae | *Wissadula amplissima* (L.) R. E. Fr. | - | - | SH | LDF, MSDF, SV | NA | CA, QR, YU | + | + | + |  |  | + | + | + | + | + | + | + |
| Marantaceae | *Maranta arundinacea* L. | Platanillo | Chan k’ala | HE | LDF, MSDF, MSTF, RV | NA | CA, YU |  |  |  |  |  | + | + | + | + | + | + |  |
| Melanthiaceae | *Schoenocaulon yucatanense* Brinke | - | - | HE | LDF, MSDF | NA, EN | CA, QR, YU |  |  |  |  | + |  | + |  |  |  |  |  |
| Melastomataceae | *Clidemia octona* (Bonpl.) L. O. Williams | Tesuate manzana | - | SH | EVF, MSTF | NA | CA, QR | + | + | + | + | + | + | + | + | + | + | + | + |
| Melastomataceae | *Henriettea succosa* (Aubl.) DC. | - | - | SH | MSDF, SAV | CU | QR |  |  |  |  |  | + |  |  | + |  | + |  |
| Melastomataceae | *Miconia ciliata* (Rich.) DC. | - | - | SH | LDF, LIFT, SAV | NA | CA, QR | + | + | + | + | + | + | + | + | + | + | + | + |
| Melastomataceae | *Miconia prasina* (Sw.) DC. | Camasey blanco | - | SH | MSDF | NA | QR |  | + | + | + | + |  |  |  |  |  |  |  |
| Meliaceae | *Azadirachta indica* A. Juss | Neem | - | TR | SV | CU | CA, QR, YU | + | + | + | + | + | + | + | + | + | + | + | + |
| Meliaceae | *Cedrela odorata* L. | Cedro español | - | TR | EVF, LDF, MSTF, MSDF, SV | NA | CA, QR, YU |  |  |  |  | + | + | + | + | + | + |  |  |
| Meliaceae | *Swietenia macrophylla* King | Caoba | - | TR | MSTF | NA | CA, QR, YU | + |  |  | + | + | + |  |  |  |  | + | + |
| Meliaceae | *Trichilia americana* (Sessé & Moc.) T. D. | Coyolillo | - | TR | LDF, MSDF, MSTF, SV | NA | CA, QR, YU |  |  |  |  | + | + |  |  |  | + |  |  |
| Meliaceae | *Trichilia glabra* L. | - | Ch’oben che’ | TR | LDF, SV | NA | CA, QR, YU |  |  |  | + | + | + |  | + | + |  |  | + |
| Meliaceae | *Trichilia hirta* L. | Cabo de hacha | - | TR | MSDF, SV | NA | CA, QR, YU |  |  | + |  | + |  | + |  |  |  | + |  |
| Moraceae | *Brosimum alicastrum* Sw. subsp. *alicastrum* | Ramón | - | TR | MSTF | NA | CA, QR, YU |  |  |  |  |  |  | + | + |  |  |  | + |
| Moraceae | *Ficus cotinifolia* Kunth | Alamo | Kopo’ | TR | CD, LDF, MSDF | NA | CA, QR, YU | + |  | + | + | + | + | + | + | + | + | + |  |
| Moraceae | *Maclura tinctoria* (L.) D. Don ex Steud. | Mora | Chak oox | TR | LDF, MSTF | NA | CA, QR, YU |  |  |  |  | + | + | + | + | + |  |  |  |
| Moraceae | *Trophis racemosa* (L.) Urb. | Ramón colorado | Sak oox | TR | LTF, MSDF | NA | CA, QR, YU | + | + | + |  |  |  |  | + | + | + | + | + |
| Moringaceae | *Moringa oleifera* Lam. | Moringa | - | TR | SV | CU | CA, QR, YU |  |  |  | + |  |  | + | + | + | + |  |  |
| Muntingiaceae | *Muntingia calabura* L. | Capulín | - | TR | EVF, CD, LIFT, LTF, MSDF, MSTF, SV | NA | CA, QR, YU | + | + | + | + | + | + | + | + | + | + | + | + |
| Musaceae | *Musa × paradisiaca* L. | Banano | - | HE | SV | CU | CA, QR, YU |  |  |  |  |  |  |  |  | + | + | + |  |
| Myricaceae | *Morella cerifera* (L.) Small | Arrayán | - | TR | LIFT, SAV | NA | QR | + | + | + | + | + | + | + | + | + | + | + | + |
| Myrtaceae | *Calyptranthes millspaughii* Urb. | - | - | TR | LDF, MSDF, MSTF | NA | QR |  |  | + | + | + |  |  |  |  |  |  |  |
| Myrtaceae | *Calyptranthes pallens* Griseb. | - | - | TR | LDF, MSTF, RV | NA | QR, YU |  |  | + |  | + | + |  |  |  |  |  |  |
| Myrtaceae | *Eugenia acapulcensis* Steud. | - | - | SH | LDF, LIFT, MSDF, MSTF | NA | CA, QR, YU |  |  |  |  |  |  | + | + | + |  |  |  |
| Myrtaceae | *Eugenia aeruginea* DC. | - | - | SH | LSDF, MSDF | NA | CA, QR |  |  |  |  |  |  |  | + |  |  | + |  |
| Myrtaceae | *Eugenia axillaris* (Sw.) Willd. | Guayabillo | - | TR | CDS, EVF, LDF, MG, MSTF | NA | CA, QR, YU |  |  | + | + | + | + | + | + | + | + |  |  |
| Myrtaceae | *Eugenia biflora* (L.) DC. | - | - | TR | MSTF, SV | NA | QR, YU |  |  | + | + |  |  |  |  | + |  |  |  |
| Myrtaceae | *Eugenia capuli* (Schlech. & Cham.) Hook & Arn. *var. capuli* | - | - | TR | LDF, MSDF, MSTF | NA | CA, QR | + | + |  | + |  |  |  | + | + |  |  |  |
| Myrtaceae | *Eugenia foetida* Pers. | - | Sak loob | SH | EVF, LDF, LIFT, MSDF, MSTF | NA | CA, QR, YU |  |  |  | + | + | + |  |  |  |  |  |  |
| Myrtaceae | *Eugenia karwinskyana* O. Berg | - | - | TR | LDF, MSDF, MSTF | NA | CA, QR, YU |  | + | + | + | + | + | + | + |  |  |  |  |
| Myrtaceae | *Eugenia laevis* O. Berg | - | - | SH | MSTF | NA | CA, QR, YU |  |  | + | + |  | + |  |  |  |  |  |  |
| Myrtaceae | *Eugenia rhombea* (O. Berg) Krug & Urb. | - | - | TR | LDF | NA | CA, QR, YU |  |  |  |  |  |  | + | + | + | + |  |  |
| Myrtaceae | *Eugenia tikalana* Lundell | - | - | TR | MSTF | NA | CA, QR, YU | + |  |  |  | + |  |  |  | + | + |  |  |
| Myrtaceae | *Eugenia trikii* Lundell | - | - | TR | EVF, MSTF | NA, EN | CA, QR |  |  |  | + | + | + | + | + | + | + |  |  |
| Myrtaceae | *Eugenia winzerlingii* Standl. | - | - | TR | LDF, LIFT, MSTF | NA, EN | CA, QR, YU | + | + | + | + |  |  |  |  |  | + | + | + |
| Myrtaceae | *Eugenia foetida* Pers. | - | Sak loob | SH | MSDF | NA | CA, QR, YU |  |  |  |  | + | + | + | + |  |  |  |  |
| Myrtaceae | *Mosiera contrerasii* (Lundell) Landrum | - | - | TR | MSTF | NA, EN | QR |  |  |  | + | + | + | + | + |  |  |  |  |
| Myrtaceae | *Myrcianthes fragrans* (Sw.) McVaugh | Guayabillo | Koj kaan | TR | LDF, MSDF, SV | NA | CA, QR, YU | + |  | + |  |  | + | + |  | + |  |  |  |
| Myrtaceae | *Pimenta dioica* (L.) Merr. | Pimienta de tabasco | Boox pool | TR | LSDF, MSTF | NA | CA, QR, YU |  | + | + |  |  |  | + | + | + | + |  |  |
| Myrtaceae | *Psidium guajava* L. | Guayaba | Pichi | TR | MSTF | CU | CA, QR, YU | + |  |  |  | + | + |  | + | + | + | + |  |
| Myrtaceae | *Psidium guineense* Sw. | Güísaro | Puul hembra | SH | SAV, SV | NT | CA, YU |  |  |  |  | + |  |  | + |  | + |  |  |
| Myrtaceae | *Psidium sartorianum* (O. Berg) Nied. | Guayabillo | Pichi che’ | SH | EVF, LDF, MSDF, MSTF | NA | CA, QR, YU | + |  |  | + |  | + | + |  | + |  |  | + |
| Nyctaginaceae | *Boerhavia coccinea* Mill. | Tostón de cuba | Pants’iil | HE | SV | NA | SR |  |  |  | + |  |  |  | + | + |  |  |  |
| Nyctaginaceae | *Boerhavia erecta* L. | Hierba blanca | Sak xiiw | HE | CD, LDF, MG, MSTF | NA | CA, QR, YU |  |  | + | + |  | + | + | + | + |  | + | + |
| Nyctaginaceae | *Commicarpus scandens* (L.) Standl. | Xicomini | - | HE | CD, LDF | NA | CA, YU |  | + |  | + |  |  | + | + |  | + | + |  |
| Nyctaginaceae | *Mirabilis jalapa* L. | Maravilla | Ts’uts’uy xiiw | HE | MSTF, SV | NT | CA, QR, YU |  |  |  |  |  |  | + | + | + | + | + |  |
| Nyctaginaceae | *Neea choriophylla* Standl. | Pinta uña | Ta’tsi’ | TR | LDF, MSDF, MSTF | NA | CA |  |  |  |  |  |  | + | + |  |  | + | + |
| Nyctaginaceae | *Neea fagifolia* Heimerl | - | - | SH | LDF, MSTF, SV | NA | CA, QR, YU | + |  |  |  |  | + |  | + |  |  |  |  |
| Nyctaginaceae | *Neea psychotrioides* Donn. Sm. | Pintadillo | Ta’tsi’ | SH | LDF, MSDF, MSTF | NA | CA, QR, YU |  |  |  | + | + | + | + | + | + | + |  |  |
| Nyctaginaceae | *Nymphaea ampla* (Salisb.) DC. | Sol de agua | Nikte’ha | HE | AV, RV | NA | CA, QR, YU | + | + | + | + | + | + | + | + | + | + | + |  |
| Nyctaginaceae | *Nymphaea jamesoniana* Planch. | - | - | HE | AV, LIFT | NA | CA, YU |  |  |  |  |  |  |  |  | + | + | + |  |
| Nyctaginaceae | *Okenia hypogaea* Schltdl. & Cham. | - | - | HE | CD | NA | CA, QR, YU |  |  |  | + | + | + | + | + | + | + |  |  |
| Nyctaginaceae | *Pisonia aculeata* L. | Uña de gato | Béeb | WC | EVF, LDF, LIFT, MSTF | NA | CA, QR, YU | + | + | + | + | + |  |  |  |  | + | + | + |
| Nymphaeaceae | *Cabomba palaeformis* Fassett | - | - | HE | AV, MG, MSDF | NA | CA, QR |  |  |  |  |  |  | + | + | + | + | + |  |
| Ochnaceae | *Ouratea lucens* (Kunth) Engl. | Laurel de monte | Buy ch’iich | SH | EVF, MSTF, SAV | NA | CA, QR | + | + | + | + | + |  |  |  |  |  |  |  |
| Olacaceae | *Schoepfia schreberi* J. F. Gmel. | - | Sak beek | TR | MSDF, MSTF | NA | CA, QR, YU | + | + |  | + | + |  |  |  | + | + | + | + |
| Oleaceae | *Forestiera rhamnifolia* Griseb var. *rhamnifolia* | - | - | SH | CDS | NA | QR |  |  |  | + |  |  |  |  |  |  |  |  |
| Oleaceae | *Jasminum officinale* L. | - | - | SH | LDF | CU | CA, QR, YU |  |  |  |  |  |  | + |  |  | + |  |  |
| Oleaceae | *Jasminum sambac* (L.) Aiton | Sampaguita | - | SH | SV | CU | CA, QR, YU |  |  |  |  | + | + | + | + |  |  |  |  |
| Onagraceae | *Ludwigia octovalvis* (Jacq.) P. H. Raven | - | - | HE | LDF, MSTF, SAV, SV | NA | CA, QR, YU |  |  | + | + | + | + |  |  | + | + | + | + |
| Onagraceae | *Ludwigia sedoides* (Humb. & Bonpl.) H. Hara | Hoja de sol | - | HE | AV, SV | NA | CA, QR, YU |  | + | + |  |  |  |  |  | + | + | + |  |
| Orobanchaceae | *Buchnera pusilla* Kunth | - | Kabalchichibe | HE | LSDF, SAV | NA | CA | + |  |  |  | + |  | + |  |  |  | + | + |
| Orobanchaceae | *Castilleja arvensis* Schltdl. & Cham | - | Catoxitzi | HE | EVF, LIFT | NA | CA, QR, YU |  | + | + | + |  | + |  |  |  |  |  |  |
| Passifloraceae | *Passiflora bicornis* Houst. ex Mill | Ojo de luna | Poch k’aak’ | HC | SV | NA | QR, YU |  |  |  |  |  | + | + | + |  |  |  |  |
| Passifloraceae | *Passiflora ciliata* Aiton | - | Poch k’aak’ | HC | LDF, MSDF | NA | CA, YU | + | + | + | + | + | + | + | + | + | + | + | + |
| Passifloraceae | *Passiflora foetida* L. | Damiana | Poch | HC | CD, LDF, MSDF, MSTF, SV | NA | CA, QR, YU |  |  | + | + |  | + | + | + | + | + |  | + |
| Passifloraceae | *Passiflora helleri* Peyr. | - | - | HC | EVF, MSTF | NA | CA, QR, YU |  | + | + | + |  |  |  |  |  |  |  |  |
| Passifloraceae | *Passiflora mayarum* J. M. MacDougal | - | Toon ts’iimim | HC | EVF, MSTF | NA | QR |  |  | + | + | + | + |  |  |  |  | + | + |
| Passifloraceae | *Passiflora sublanceolata* (Killip) J. M. MacDougal | - | - | HC | LIFT, MSTF, SAV, SV | NA, EN | CA, QR | + |  | + | + |  | + | + |  | + |  |  |  |
| Passifloraceae | *Passiflora yucatanensis* Killip | - | - | HC | LIFT, MSTF | NA, EN | CA, QR, YU |  |  |  |  | + | + | + | + | + |  |  |  |
| Passifloraceae | *Piriqueta cistoides* (L.) Griseb. | - | - | HE | LIFT, SAV | NA | CA, QR |  |  | + | + |  | + |  | + | + |  |  |  |
| Passifloraceae | *Turnera diffusa* Willd. ex Schult. | Damiana | Miisib kook | SH | CD, LDF, MSDF, SV | NA | CA, YU |  | + | + | + | + | + | + | + | + | + | + | + |
| Passifloraceae | *Turnera odorata* Rich. | - | - | SH | EVF, MSTF, SV | NA | CA, QR, YU | + | + |  |  | + | + | + | + |  |  |  |  |
| Passifloraceae | *Turnera ulmifolia* L. | Clavel de oro | - | SH | CD, LDF, MSTF, SV | NA | CA, QR, YU |  |  | + |  | + | + |  |  |  | + | + |  |
| Pedaliaceae | *Sesamum indicum* L. | Sésamo | - | HE | LDF, LTF | CU | CA, QR, YU |  |  |  |  |  |  |  | + |  | + |  |  |
| Phyllanthaceae | *Astrocasia tremula* (Griseb.) G. L. Webster | Trompillo | Kabal piix t’oom | TR | CD, LDF, MG, MSDF | NA | CA, QR, YU | + | + | + | + | + | + | + | + | + | + | + | + |
| Phyllanthaceae | *Phyllanthus acuminatus* Vahl | Chirrincha | - | SH | EVF, LDF, MSDF, MSTF | NA | CA, QR, YU |  |  |  |  |  | + | + | + | + |  |  |  |
| Phyllanthaceae | *Phyllanthus grandifolius* L. | Cascabel | Piix t’ oom | SH | MSDF, MSTF, SV | NA | CA, QR, YU |  |  |  |  |  |  | + | + | + | + |  |  |
| Phyllanthaceae | *Phyllanthus graveolens* Kunth | Chin chin pool ojo | Piix t’ oom | SH | LDF | NA | CA, QR, YU |  |  |  |  |  | + | + | + | + |  |  |  |
| Phyllanthaceae | *Phyllanthus mocinianus* Baill. | - | - | SH | LDF, MSDF, MSTF, SV | NA | CA, YU |  |  | + |  | + | + | + |  |  |  |  | + |
| Phyllanthaceae | *Phyllanthus liebmannianus* Müll. Arg. | Chin chin pool ojo | Piix t’oom ojo | HE | EVF, LDF, LIFT, SV | NA | CA, QR, YU |  | + | + | + |  |  | + | + | + | + |  |  |
| Phyllanthaceae | *Phytolacca rivinoides* Kunth & C. D. Bouché | Jaboncillo | Tees | HE | EVF, LDF, SV | NA | CA, QR, YU | + | + |  | + |  |  | + |  |  |  |  |  |
| Picramniaceae | *Picramnia antidesma* Sw. | - | K’anchin aak | SH | CDS, EVF, LDF, LIFT, MSTF | NA | QR, YU |  |  | + |  |  |  | + | + | + |  |  |  |
| Piperaceae | *Piper marginatum* Jacq. | Cordoncillo | Ya’ax pe’ejel che | SH | EVF, LDF, MSTF, MSDF | NA | CA, QR, YU | + | + | + | + | + | + | + | + | + | + | + | + |
| Piperaceae | *Piper neesianum* C. DC. | - | - | SH | EVF, MSTF | NA | CA, QR, YU |  | + | + | + | + | + |  |  |  |  |  |  |
| Piperaceae | *Piper yucatanense* C. DC. | - | - | SH | EVF, MSTF | NA | CA, QR, YU |  | + | + | + | + | + | + |  |  |  |  |  |
| Plantaginaceae | *Bacopa monnieri* (L.) Wettst. | - | Xanab mukuy | HE | AV, CD, CDS, MG, LDF, MSTF, SAV | NA | CA, YU |  | + | + | + | + | + |  |  | + | + | + |  |
| Plantaginaceae | *Russelia campechiana* Standl. | Corrimiento | Aak | HE | EVF, LDF, MSTF, SAV | NA | CA, QR, YU | + | + | + | + |  |  |  |  |  |  |  | + |
| Poaceae | *Bouteloua dimorpha* Columbus |  | Jáayal su’uk | HE | LDF, VS | NA | CA, YU |  |  |  |  | + |  |  |  | + | + | + | + |
| Poaceae | *Imperata contracta* (Kunth) Hitchc. | Zacate amarillo | Ek’chim | HE | SAV, SV | NA | CA |  |  |  |  |  | + | + | + | + |  |  |  |
| Poaceae | *Ischaemum latifolium* (Spreng.) Kunth | - | - | HE | SAV | NA | QR |  |  |  |  | + | + | + |  |  |  |  |  |
| Poaceae | *Lasiacis grisebachii* (Nash) Hitchc. var. *grisebachii* | - | - | HE | EVF, MSDF | NA | CA |  |  |  |  |  |  | + | + | + | + |  |  |
| Poaceae | *Monanthochloe littoralis* Engelm. | - | - | HE | CD, MG | NA | YU |  |  |  |  |  |  | + | + | + |  |  |  |
| Poaceae | *Paspalum blodgettii* Chapm. | - | K’Anchin | HE | LDF, MSDF, SV | NA | CA, QR, YU |  |  |  |  |  | + | + | + | + |  |  |  |
| Poaceae | *Zea mays* L. | Maíz | - | HE |  | CU | CA |  |  |  |  |  |  |  |  |  |  |  |  |
| Polygonaceae | *Antigonon leptopus* Hook. & Arn. | Flor de San Diego | Chak lool | HC | LDF, SV | NT | CA, QR, YU |  |  |  | + | + | + | + | + | + | + | + |  |
| Polygonaceae | *Coccoloba acapulcensis* Standl. | - | Tóon yúul | TR | EVF, LTF, LSDF, LIFT, MDF, MSTF | NA | CA, QR, YU |  |  |  |  |  | + | + |  |  |  |  |  |
| Polygonaceae | *Coccoloba barbadensis* Jacq. | Buen amigo | Boob ch’iich’ | TR | EVF, LDF, MG, MDF, MSTF | NA | CA, QR, YU | + |  |  |  | + | + | + |  |  |  |  |  |
| Polygonaceae | *Coccoloba belizensis* Standl. | - | - | TR | EVF, SV | NA | QR |  |  |  |  | + | + | + | + | + | + |  |  |
| Polygonaceae | *Coccoloba cozumelensis* Hemsl. | - | Boob | TR | EVF, LIFT, LDF, MSTF, SV | NA | CA, QR, YU |  |  |  | + | + | + | + | + |  |  |  | + |
| Polygonaceae | *Coccoloba diversifolia* Jacq. | Uvero | Ch’iich’ boob | SH | CD, LIFT, MSTF, SV | NA | QR |  |  |  | + | + | + | + |  |  |  |  |  |
| Polygonaceae | *Coccoloba humboldtii* Meins. | Tocó prieto | - | TR | CD, MSTF, SV | NA | CA, QR |  | + | + | + |  |  |  |  |  |  | + | + |
| Polygonaceae | *Coccoloba ortizii* R. A. Howard | - | - | TR | CD, LTF, MSTF | NA, EN | QR, YU | + |  |  |  | + |  |  |  |  |  |  |  |
| Polygonaceae | *Coccoloba reflexiflora* Standl. | - | Bobchiché | TR | AV, LDF, MSTF | NA | QR, YU | + | + |  |  |  |  | + |  | + |  | + | + |
| Polygonaceae | *Coccoloba spicata* Lundell | - | Boob ch’iich’ | TR | MSTF, SV | NA | CA, QR, YU | + | + | + | + | + | + | + | + | + | + | + | + |
| Polygonaceae | *Coccoloba swartzii* Meins. | - | - | TR | LDF, MSTF | NA | QR |  |  |  | + |  |  |  | + | + |  |  |  |
| Polygonaceae | *Coccoloba uvifera* (L.) L. | Uva de mar | Ni’ che | TR | CD, CDS, | NA | CA, QR, YU | + | + | + | + | + | + | + | + | + | + | + | + |
| Polygonaceae | *Gymnopodium floribundum* Rolfe | - | Ts’iits’ilche’ | TR | CD, LDF, LIFT | NA | CA, QR, YU | + | + | + | + | + | + |  |  |  | + | + | + |
| Polygonaceae | *Neomillspaughia emarginata* (H. Gross) S. F. Blake | - | Sak iitsa’ | SH | LDF, MDF, MSTF, SV | NA, EN | CA, QR, YU |  |  |  |  | + | + | + | + | + | + | + |  |
| Polygonaceae | *Persicaria acuminata* (Kunth) M. Gómez | - | - | HE | AV | NA | QR, YU |  |  | + |  |  |  |  |  |  | + | + |  |
| Polygonaceae | *Persicaria hydropiperoides* (Michx.) Small | Quemaculo | - | HE | AV | NA | CA, QR, YU |  |  |  |  |  | + |  |  |  | + | + |  |
| Polygonaceae | *Persicaria punctata* (Elliott) Small | Chilillo | Chili | HE | AV | NA | CA, QR |  |  |  |  |  | + |  |  | + | + | + |  |
| Polygonaceae | *Podopterus mexicanus* Humb. & Bonpl. | Espuela de gallo | Puuts mukuy | SH | LDF, LDFCC | NA | CA, YU |  |  | + | + | + |  | + |  |  |  |  |  |
| Polygonaceae | *Polygonum segetum* Kunth | Chilillo hierba del camarón | - | HE | AV | NA | CA, QR |  |  |  |  |  |  |  |  | + | + | + |  |
| Polygonaceae | *Ruprechtia chiapensis* Lundell | - | - | SH | EVF, LDF, LIFT, MSDF, MSTF | NA | CA, YU | + | + | + | + |  |  |  |  |  |  |  |  |
| Portulacaceae | *Portulaca oleracea* L. | Verdolaga | Saioch tsay | HE | CD, LDF, MG, MSTF, SAV | NA | CA, QR, YU |  | + | + | + | + | + | + | + | + | + |  |  |
| Portulacaceae | *Portulaca pilosa* L. | Suculenta | Saioch tsay | HE | CD, LDF, LIFT | NA | CA, QR, YU | + |  | + | + |  | + | + | + | + |  | + |  |
| Primulaceae | *Ardisia escallonioides* Schltdl. & Cham. | Pimienta de monte | Sak loob che' | SH | LDF, LIFT, MSDF | NA | CA, QR, YU |  | + |  |  |  |  |  |  |  | + | + | + |
| Primulaceae | *Myrsine cubana* A. DC. | - | - | SH | EVF, LIFT, MSTF | NA | CA, QR | + |  | + | + | + |  | + |  |  |  |  |  |
| Ranunculaceae | *Clematis dioica* L. | Pelo de ángel | Me’ex nojoch máak | WC | LDF, LIFT, LTF, MSTF | NA | CA, QR, YU | + | + |  |  |  |  |  |  |  | + | + | + |
| Ranunculaceae | *Clematis polygama* Jacq. | - | - | WC | LIFT | NA | CA, QR | + | + | + |  |  | + |  |  |  |  | + |  |
| Rhamnaceae | *Colubrina arborescens* (Mill.) Sarg. | - | Pimienta ché | TR | EVF, LDF, MG, MSTF | NA | CA, QR, YU | + | + |  |  |  |  |  |  |  | + | + | + |
| Rhamnaceae | *Colubrina elliptica* (Sw.) Brizicky & W. L. Stern | - | - | SH | LDF, LIFT | NA | CA, QR, YU | + |  |  |  |  |  | + | + | + | + | + |  |
| Rhamnaceae | *Colubrina greggii* S. Watson *var. yucatanensis* M. C. Johnst | Pimienta che’ | Puukin, tsúulub máay | WC | LDF, MG | NA, EN | CA, QR, YU | + | + | + | + | + | + | + | + | + | + | + | + |
| Rhamnaceae | *Gouania lupuloides* (L.) Urb. | - | Chéen máak | SH | LDF, MSTF | NA | CA, QR, YU |  |  |  | + |  |  |  |  | + | + | + |  |
| Rhamnaceae | *Gouania polygama* (Jacq.) Urb. | Ojitos | - | SH | EVF, MSTF | NA | CA, QR |  |  |  |  |  |  |  |  |  | + | + |  |
| Rhamnaceae | *Krugiodendron ferreum* (Vahl) Urb. | Quiebra hacha | Ch'iin took' | HE | LDF, MSTF | NA | CA |  |  | + |  | + |  | + |  |  |  |  |  |
| Rhamnaceae | *Ziziphus yucatanensis* Standl. | - | - | TR | LDF, MSTF | NA, EN | QR, YU |  |  |  |  | + | + |  |  |  |  |  |  |
| Rhizophoraceae | *Rhizophora mangle* L. | Mangle | Ta’ab che’ | TR | CD, CDS, LIFT, MG, RV | NA | CA, QR, YU |  | + | + | + | + | + | + | + | + |  |  |  |
| Rubiaceae | *Alseis yucatanensis* Standl. | Manzanillo | Ja’as che’ | TR | MSDF, MSTF | NA, EN | CA, QR, YU | + |  | + | + | + | + | + | + | + | + | + | + |
| Rubiaceae | *Asemnantha pubescens* Hook. f. | Juan de nochein | Mch’iilib tuux | SH | LDF, LIFT, MSDF, MSTF | NA, EN | CA, QR, YU |  |  | + | + | + | + | + | + | + | + | + | + |
| Rubiaceae | *Blepharidium guatemalense* Standl. | - | - | SH | LDF, MSDF, MSTF | NA | CA, YU | + | + | + | + | + | + | + | + | + | + | + | + |
| Rubiaceae | *Calycophyllum candidissimum* (Vahl) DC | Aldama | - | TR | MSDF, MSTF | NA | CA | + |  |  |  |  |  |  |  | + | + | + | + |
| Rubiaceae | *Chiococca alba* (L.) Hitchc. | Huele de noche | Chakan che’ | SH | CDS, LDF, LDFCC, LIFT, MG, MSDF, MSTF, SV | NA | CA, QR, YU | + | + | + | + | + | + | + | + | + | + | + | + |
| Rubiaceae | *Cosmocalyx spectabilis* Standl. | - | Chakte’ kook | HE | MSDF | NA | CA |  |  |  | + | + | + | + | + | + | + | + |  |
| Rubiaceae | *Coutarea hexandra* (Jacq.) K. Schum. | Palo de quina | Taastaab | SH | LDF, LDFCC, LIFT | NA | CA, QR, YU |  |  |  | + | + | + | + | + | + | + |  |  |
| Rubiaceae | *Erithalis fruticosa* L. | Manglillo | - | SH | CD, CDS, LDF | NA | QR, YU | + | + |  |  | + | + | + | + | + | + | + | + |
| Rubiaceae | *Exostema caribaeum* (Jacq.) Roem. & Schult. | Copalche | Pay luuk | SH | LDF, MSDF, MSTF | NA | CA, QR, YU |  |  |  |  |  |  | + | + | + | + | + |  |
| Rubiaceae | *Guettarda combsii* Urb. | Manzanillo | Payluk taastaab | TR | CD, EVF, MG, SAV | NA | CA, QR, YU |  |  | + | + | + | + |  |  |  | + |  |  |
| Rubiaceae | *Guettarda elliptica* Sw. | - | Lu’um che’ | SH | LDF, LSDF, MSDF, MSTF, SV | NA | CA, QR, YU |  | + |  |  | + | + | + | + | + | + |  | + |
| Rubiaceae | *Hamelia patens* Jacq. | Coloradillo | Chak took’ | SH | LDF, LIFT, MSDF, MSTF, SV | NA | CA, QR, YU | + | + | + | + | + | + | + | + | + | + | + | + |
| Rubiaceae | *Hintonia octomera* (Hemsl.) Bullock | - | Xpay lu’uch | TR | LDF, LIFT, MSDF, MSTF | NA, EN | CA, QR, YU |  |  |  |  |  |  | + | + | + | + | + | + |
| Rubiaceae | *Ixora finlaysoniana* Wall. ex G. Don | Coralillo | - | SH | SV | CU | CA, QR, YU | + | + | + | + | + | + | + | + | + | + | + | + |
| Rubiaceae | *Machaonia lindeniana* Baill. | - | K’uch’eel | TR | LDF, LIFT, MSDF, MSTF | NA, EN | CA, QR, YU |  |  |  | + | + | + | + | + | + | + |  |  |
| Rubiaceae | *Margaritopsis microdon* (DC.) C. M. Taylor | Dama de campo | Baake aak | WC | LDF, LIFT, MSDF, MSTF, SV | NA | CA, QR, YU |  |  |  | + | + | + | + | + |  |  |  |  |
| Rubiaceae | *Morinda royoc* L. | Piña de monte | Piña aak’ | HC | LDF, LDFCC, SV | NA | CA, QR, YU | + | + | + | + | + | + | + |  |  |  |  |  |
| Rubiaceae | *Psychotria costivenia* Griseb. var. *costivenia* | - | - | SH | MSDF, MSTF | NA | CA, QR | + | + | + | + | + | + |  |  |  |  |  |  |
| Rubiaceae | *Psychotria fruticetorum* Standl. | - | - | SH | EVF, MSDF, MSTF | NA | CA, QR | + | + | + | + | + | + | + | + | + | + | + | + |
| Rubiaceae | *Psychotria nervosa* Sw. | Retamo | K’aanan, ya’ax anal | SH | LDF, LIFT, LDFCC, MSDF, MSTF | NA | CA, QR, YU | + | + | + | + | + | + | + | + | + | + | + | + |
| Rubiaceae | *Psychotria pubescens* Sw. | - | K’Aanan | SH | MSDF, MSTF | NA | CA, QR, YU |  |  |  | + | + | + | + | + | + | + | + |  |
| Rubiaceae | *Rachicallis americana* (Jacq.) Hitchc. | - | - | SH | CD, CDS | NA | QR |  |  | + |  |  |  |  |  |  |  |  |  |
| Rubiaceae | *Randia aculeata* L. | Tinta che | Cruz k’iix | SH | CDS, EVF, LDF, LIFT, LDFCC, MSDF, MSTF | NA | CA, QR, YU |  |  | + | + | + | + | + |  |  |  |  |  |
| Rubiaceae | *Randia laetevirens* Standl. | Crucillo | Kabal k’aax | SH | LDF, MSTF, SV | NA | CA, YU |  |  | + |  |  | + |  | + |  |  |  |  |
| Rubiaceae | *Randia monantha* Benth. | Papache peludo | - | SH | LIFT | NA | CA, YU |  |  |  |  | + | + |  |  |  |  |  |  |
| Rubiaceae | *Randia obcordata* S. Watson | Crucetillo | Cruz k’iix | SH | LDF, LDFCC, LIFT, MSDF, MSTF, SV | NA | CA, QR, YU |  | + |  |  |  | + |  |  |  |  |  |  |
| Rubiaceae | *Randia tomatillo* Loes. | - | - | SH | CDS | NA | CA |  |  |  |  | + | + | + |  |  |  |  |  |
| Rubiaceae | *Randia truncata* Greenm. & C. H. Thomps. | Crucetillo | Cruz k’iix | SH | LDF, LDFCC, MSDF, MSTF, SV | NA, EN | CA, QR, YU |  |  | + | + | + | + |  |  |  |  |  |  |
| Rubiaceae | *Richardia scabra* L. | Ipecacuana amilácea | - | HE | LDF, SV | NA | CA |  | + |  |  |  |  | + |  |  |  |  |  |
| Rubiaceae | *Simira salvadorensis* (Standl.) Steyerm. | Nazareno | Chakte kok | TR | EVF, MSTF | NA | CA, QR, YU |  |  |  |  | + | + | + | + |  |  |  |  |
| Rubiaceae | *Spermacoce ovalifolia* (M. Martens & Galeotti) Hemsl. | - | - | HE | LDF, MSDF, MSTF, SV | NA | CA, QR, YU | + |  |  |  |  |  |  | + | + | + | + | + |
| Rubiaceae | *Spermacoce suaveolens* (G. Mey.) Kuntze | - | - | HE | LDF, MSDF, MSTF, SV | NA | CA, QR, YU | + | + | + | + | + | + | + | + | + | + | + | + |
| Rubiaceae | *Spermacoce tenuior* L. | Culantrillo | Kaba mul | HE | LDF, MSDF, MSTF, SV | NA | CA, QR, YU | + | + | + | + | + | + | + | + |  |  |  |  |
| Rubiaceae | *Spermacoce tetraquetra* A. Rich. | - | - | HE | LDF, MSDF, MSTF, SV | NA | CA, QR, YU | + | + | + | + |  |  |  | + | + | + | + | + |
| Rubiaceae | *Spermacoce verticillata* L. | Culantrillo | Kaba mul | HE | LDF, LIFT, LDFCC, MSDF, MSTF, SV | NA | CA, QR, YU | + | + | + | + | + | + | + | + | + | + | + | + |
| Rubiaceae | *Stenostomum lucidum* (Sw.) C. F. Gaertn | Palo de rosa | - | TR | MSDF, MSTF | NA | CA, YU |  |  |  |  | + | + | + | + | + |  |  |  |
| Rutaceae | *Amyris attenuata* Standl. | - | - | SH | MSDF | NA | QR |  |  |  |  |  |  |  |  | + |  |  |  |
| Rutaceae | *Amyris elemifera* L. | Palo de gas | K‘an chan | TR | LDF, MSDF, MSTF | NA | CA, QR, YU | + | + | + |  |  |  | + |  |  |  |  | + |
| Rutaceae | *Amyris sylvatica* Jacq. | Palo de gas | K’an yuuk | SH | EVF, MSDF | NA | CA, QR, YU |  |  |  |  |  | + |  |  |  |  |  |  |
| Rutaceae | *Casimiroa sapota* Oerst. | Matasano | - | TR | MSDF, MSTF | NA | QR | + | + |  |  |  |  |  |  |  |  |  |  |
| Rutaceae | *Casimiroa tetrameria* Millsp. | Zapote amarillo | Yuuy | TR | LDF, MSDF, MSTF | NA | CA, QR, YU | + |  |  |  |  |  |  |  |  |  |  | + |
| Rutaceae | *Citrus aurantiaca* L. | - | - | TR | SV | CU | CA, QR, YU |  |  | + |  |  |  |  |  |  |  |  |  |
| Rutaceae | *Citrus paradisi* Macfad. | Pomelo toronja | - | TR | LDF, SV | CU | CA, QR, YU | + |  |  | + | + |  |  |  |  |  |  |  |
| Rutaceae | *Citrus reticulata* Blanco | Mandarino | - | TR | SV | CU | CA, QR, YU |  |  |  | + |  |  |  |  |  | + |  |  |
| Rutaceae | *Citrus sinensis* (L.) Osbeck. | Naranjo dulce | - | TR | LDF, SV | CU | CA, QR, YU |  |  | + |  |  | + |  |  |  | + |  |  |
| Rutaceae | *Cítrus × aurantium* L. | Naranjo amargo | - | TR | SV | CU | CA, QR, YU |  | + |  |  |  | + |  |  |  | + |  |  |
| Rutaceae | *Citrus × aurantiifolia* (Christm.) Swingle | Limonero | - | TR | LDF, SV | CU | CA, QR, YU |  | + |  | + |  |  |  |  |  |  |  |  |
| Rutaceae | *Esenbeckia berlandieri* Baill. ex Hemsl. | Paguay | Tankasche’ | TR | CDS, LDF | NA | CA, QR, YU |  |  |  |  |  |  | + |  |  |  |  |  |
| Rutaceae | *Esenbeckia pentaphylla* (Macfad.) Griseb. | Naranja che | Jo’k’o | SH | LDF, MSDF, MSTF | NA | CA, QR, YU |  |  |  |  |  |  | + | + | + |  |  |  |
| Rutaceae | *Murraya paniculata* (L.) Jack | Naranjo jazmín | - | SH | SV | CU | CA, QR, YU |  |  |  |  |  |  |  |  | + |  |  |  |
| Rutaceae | *Zanthoxylum caribaeum* Lam. | Rabo de lagarto | Na’an che’ | SH | LDF | NA | CA, QR, YU | + | + |  |  |  |  |  |  |  |  |  |  |
| Rutaceae | *Zanthoxylum fagara* (L.) Sarg. | Limoncillo | Na’an che’ | SH | LDF, MSDF, MSTF | NA | CA, QR, YU |  | + |  | + |  |  |  |  |  |  |  | + |
| Rutaceae | *Zanthoxylum juniperinum* Poepp. | Rabo de lagarto | Na’an che’ | TR | MSDF, MSTF | NA | CA, QR |  |  |  | + |  |  |  |  |  |  |  |  |
| Salicaceae | *Casearia aculeata* Jacq. | Chatay | Ts’iu che | TR | EVF, LDF, MSTF | NA | CA, QR |  |  |  |  |  | + |  |  |  |  | + |  |
| Salicaceae | *Casearia corymbosa* Kunth | Palo de piedra costeño | Ix iim che | TR | LDF, MSTF | NA | CA, QR, YU |  |  |  | + | + | + |  |  |  |  |  |  |
| Salicaceae | *Casearia emarginata* C. Wright ex Griseb. | Naranja che’ | Am che’ | SH | LDF, MSTF | NA | CA, QR, YU |  |  | + |  |  |  | + |  |  |  |  | + |
| Salicaceae | *Laetia thamnia* L. | Zapote amarillo | Ix iim che’ | TR | EVF, LTF, MSDF | NA | CA, QR, YU |  |  |  | + | + |  |  |  |  |  |  |  |
| Salicaceae | *Samyda yucatanensis* Standl. | - | Puuts’ mukuy | SH | LDF, MSDF, SV | NA, EN | CA, QR, YU | + |  | + | + | + | + |  |  |  |  |  |  |
| Salicaceae | *Xylosma flexuosa* (Kunth) Hemsl. | - | - | SH | LDF, LIFT, MSTF, MG | NA | CA, QR, YU | + |  |  |  |  |  |  |  |  |  | + | + |
| Salicaceae | *Zuelania guidonia* (Sw.) Britton & Millsp. | Volador | Ta’May | TR | EVF, LDF, MSDF, MSTF | NA | CA, QR, YU |  |  | + | + | + |  |  |  |  |  |  |  |
| Sapindaceae | *Allophylus camptostachys* Radlk. | Cuachichile | K’an chuunup | TR | MSDF | NA | CA, QR, YU | + | + |  |  |  |  |  |  |  |  | + | + |
| Sapindaceae | *Allophylus cominia* (L.) Sw. | Cordoncillo | Iik’ baach | TR | LDF, MG, LDFCC, LIFT, MSDF | NA | CA, QR, YU |  |  |  |  |  |  |  |  | + | + |  |  |
| Sapindaceae | *Cardiospermum corindum* L. | Tronadora | Paj sakan aak’ | HC | MG, LDF, LDFCC, LIFT, MSDF, MSTF, EVF | NA | CA, QR, YU | + | + | + | + | + |  |  | + | + | + | + | + |
| Sapindaceae | *Cupania belizensis* Standl. | - | Sak poom | TR | EVF, MSTF | NA | CA, QR, YU | + |  |  |  |  |  |  |  |  |  | + | + |
| Sapindaceae | *Cupania dentata* DC. | Agua al ojo blanco | - | SH | MSDF, MSTF | NA | CA, QR, YU | + |  |  | + |  |  |  |  | + | + |  |  |
| Sapindaceae | *Cupania glabra* Sw. | Cascuá | - | SH | LDF, MSDF, MSTF, EVF | NA | QR, YU | + | + |  |  |  |  |  | + | + | + | + | + |
| Sapindaceae | *Dodonaea viscosa* Jacq. | Candela | - | SH | CDS, SV | NA | QR |  |  |  |  |  |  |  |  |  |  |  | + |
| Sapindaceae | *Exothea diphylla* (Standl.) Lundell | Guayo | Wayuum | TR | SV | NA | CA, QR, YU |  | + |  |  |  |  |  |  |  |  |  |  |
| Sapindaceae | *Matayba oppositifolia* (A. Rich.) Britton. | Tempesquite | - | SH | CDS, LDF, MDF | NA | CA, QR | + | + | + |  |  |  |  |  |  |  |  |  |
| Sapindaceae | *Melicoccus oliviformis* Kunth subsp. *oliviformis* | - | - | TR | MSTF, SV | NT | CA, QR, YU | + |  | + | + |  |  |  |  |  |  |  |  |
| Sapindaceae | *Paullinia cururu* L. | - | Chéen aak' | WC | LDF, MSDF, MSTF, EVF | NA | CA, QR, YU |  |  |  |  |  | + | + |  |  |  |  |  |
| Sapindaceae | *Paullinia fuscescens* Kunth | Chilillo | Chéen aak' | WC | LIFT, LDF, MSDF, MSTF | NA | CA, QR, YU | + | + | + |  |  |  |  |  |  |  | + | + |
| Sapindaceae | *Paullinia pinnata* L. | - | Sakan aak' | WC | CDS, LIFT, LDF, MSDF, MSTF, EVF | NA | CA, QR, YU |  |  |  |  | + | + | + |  |  |  |  |  |
| Sapindaceae | *Paullinia tomentosa* Jacq. | Hierba de caballo | - | WC | MG, MSDF, EVF, MSTF | NA | CA, QR, YU |  |  |  |  |  |  |  | + | + | + | + |  |
| Sapindaceae | *Sapindus saponaria* L. | Jaboncillo | Sibul | TR | CDS, LDF, MSDF, MSTF, EVF | NA | CA, QR, YU |  | + | + |  |  |  |  |  |  |  | + | + |
| Sapindaceae | *Serjania adiantoides* Radlk. | - | Boax aak’ | HC | LDF, MSDF, MSTF, EVF | NA | CA, QR, YU | + | + | + | + | + | + | + |  |  |  | + | + |
| Sapindaceae | *Serjania goniocarpa* Radlk. | Zarzaparrilla | Buy aak’ | WC | LDF, MSDF, MSTF | NA | CA, QR, YU |  |  |  |  |  |  |  | + | + | + | + | + |
| Sapindaceae | *Serjania grosii* Standl. | - | - | WC | LDF, MSTF | NA | YU |  |  | + |  |  |  |  |  |  |  | + |  |
| Sapindaceae | *Serjania lundellii* Croat | - | - | WC | LTF | NA | CA, QR, YU |  |  | + |  |  |  |  |  |  |  |  |  |
| Sapindaceae | *Serjania mexicana* (L.) Willd. | Zarzaparrilla | Chéen aak’ | WC | LDF | NA | CA |  | + |  |  |  |  |  |  |  |  |  |  |
| Sapindaceae | *Serjania pterarthra* Standl. | - | - | WC | LDF, MSDF, MSTF, EVF, SV | NA, EN | CA, QR |  |  | + | + | + | + |  |  |  |  |  |  |
| Sapindaceae | *Serjania triquetra* Radlk. | Bejuco de tres costillas | Ch’emil aak’ | WC | MSDF | NA | CA, YU |  |  |  |  |  |  |  |  |  |  | + |  |
| Sapindaceae | *Serjania yucatanensis* Standl. | - | Chéen peek’ | WC | LDF, MSDF, MSTF, SV, EVF | NA, EN | CA, QR, YU | + | + | + |  |  |  |  |  |  |  | + | + |
| Sapindaceae | *Talisia floresii* Standl. | - | - | TR | LDF, MSDF, MSTF, EVF | NA, EN | CA, QR, YU |  |  |  | + | + | + | + | + |  |  |  |  |
| Sapindaceae | *Thinouia tomocarpa* Standl. | - | - | WC | MSTF. SA, SV | NA | CA, QR |  |  | + | + |  |  |  |  |  |  |  |  |
| Sapindaceae | *Thouinia paucidentata* Radlk. | Hueso de tigre | K’an chuunup | TR | MSTF, LSDF | NA, EN | CA, QR, YU |  |  | + |  |  |  | + | + | + | + | + | + |
| Sapindaceae | *Urvillea ulmacea* Kunth | - | Lot sak’ | WC | LDF, MSDF, MSTF, EVF | NA | CA, QR, YU | + | + | + |  |  |  |  |  |  |  | + | + |
| Sapotaceae | *Chrysophyllum cainito* L. | Caymito | - | ARB | LDF, SV, EVF | CU | CA, QR, YU |  |  |  |  |  |  |  |  |  | + |  | + |
| Sapotaceae | *Chrysophyllum mexicanum* Brandegee ex Standl. | Caymito | Chi’kéej | TR | LDF, MSTF, EVF, SV | NA | CA, QR, YU |  | + |  |  |  |  |  | + | + | + |  |  |
| Sapotaceae | *Manilkara zapota* (L.) P. Royen | Chico zapote | - | TR | MSTF | NA | CA, QR, YU |  |  |  | + | + |  | + |  |  |  |  |  |
| Sapotaceae | *Pouteria campechiana* (Kunth) Baehni | Caimitillo | Chi’kéej | TR | CD, EVF | NA | CA, QR, YU | + | + | + | + | + | + |  | + |  |  |  | + |
| Sapotaceae | *Pouteria glomerata* (Miq.) Radlk. subsp. *glomerata* | - | Chóoch | TR | LDF | NA | YU |  |  | + | + | + | + | + | + |  |  |  |  |
| Sapotaceae | *Pouteria reticulata* (Engler) Eyma. subsp. *reticulata* | Zapotillo | - | TR | MSTF, EVF | NA | CA, QR, YU |  |  |  |  | + |  | + | + | + | + |  |  |
| Sapotaceae | *Pouteria sapota* (Jacq.) H.E. Moore & Stearn | Mamey | - | TR | EVF | CU | CA, QR, YU |  |  | + | + |  |  |  |  |  |  | + |  |
| Sapotaceae | *Sideroxylon americanum* (Mill.) T. D. Penn. | Caimitillo | Mulche’ | SH | CD, CDS, LDF, MG, MSTF | NA | CA, QR, YU | + | + | + |  |  | + | + | + | + | + | + | + |
| Sapotaceae | *Sideroxylon celastrinum* (Kunth) T. D. Penn. | - | Lu'uchum che' | TR | LDF, LSDF, LIFT, SV, MSDF, MSTF, SBP | NA | CA, QR, YU | + | + |  |  | + |  |  | + |  | + |  |  |
| Sapotaceae | *Sideroxylon foetidissimum* Jacq. subsp. *gaumeri* (Pittier) Pennington | - | Sibul | TR | EVF, MSDF, MSTF | NA, EN | CA, QR, YU |  |  |  | + | + |  |  | + |  | + | + |  |
| Sapotaceae | *Sideroxylon salicifolium* (L.) Lam. | Zapote | Chakal ja'as | TR | LDF, MSTF, MSDF, SV | NA | CA, QR, YU |  |  | + | + | + |  |  |  |  |  |  |  |
| Scrophulariaceae | *Capraria biflora* L. | Claudiosa | Chokuilxim | HE | AV, CDS, RV | NA | CA, QR, YU | + | + | + | + | + | + | + | + | + | + | + | + |
| Scrophulariaceae | *Capraria frutescens* (Mill.) Britton | Claudiosa | Sek’aax bóox | HE | CD, LDF | NA | CA, QR, YU | + | + | + | + | + | + | + | + | + | + | + | + |
| Simaroubaceae | *Alvaradoa amorphoides* Liebm. subsp. *amorphoides* | Navideño | - | TR | SV | NA | CA, QR, YU | + | + | + |  |  |  |  |  |  |  | + | + |
| Simaroubaceae | *Simarouba amara* Aubl. | Pistache | Pa’ sak, paj sak iil | TR | MSDF, MSTF | NA | CA, QR, YU | + | + | + |  |  |  |  |  |  |  |  | + |
| Solanaceae | *Capsicum annuum* L. var. *glabriusculum* (Dunal) Heiser & Pickersgill | Chile maax | Maax iik | HE | LSDF | NT | CA, QR, YU |  | + |  |  |  | + | + |  |  |  |  |  |
| Solanaceae | *Capsicum frutescens* L. | - | - | HE | MSTF | CU | CA, QR, YU | + |  |  |  | + |  |  | + |  |  |  |  |
| Solanaceae | *Cestrum diurnum* L. | - | - | SH | RV | CU | CA, QR, YU |  |  | + | + | + | + | + | + | + |  |  |  |
| Solanaceae | *Cestrum nocturnum* L. | Dama de noche | Ak’a xiiw | SH | EVF, LDF, MSDF, MSTF, SV | NA | CA, QR, YU |  |  | + | + | + | + | + | + | + |  |  |  |
| Solanaceae | *Cestrum racemosum* Ruiz & Pavón | - | - | TR | MSTF, SV | NA | QR |  |  | + | + | + |  |  |  |  |  |  |  |
| Solanaceae | *Datura stramonium* L. | - | - | HE | LDF, SV | CU | CA, QR, YU | + |  | + |  | + | + | + | + |  |  | + |  |
| Solanaceae | *Lycianthes armentalis* J. L. Gentry | Diente de perro | Ich peek | SH | EVF, LIFT, MSTF, SV | NA | CA, QR, YU |  | + | + | + | + | + | + | + | + | + | + |  |
| Solanaceae | *Lycianthes hypoleuca* Standl. | - | - | SH | EVF, SV | NA | CA, QR |  |  |  |  |  |  | + | + | + | + |  |  |
| Solanaceae | *Lycianthes lenta* (Cav.) Bitter | - | - | TRE L | LDF, MSDF, MSTF, SV | NA | CA, QR, YU |  |  |  |  |  | + | + | + | + |  |  |  |
| Solanaceae | *Lycianthes limitanea* (Standl.) J. L. Gentry | - | - | WC | EVF, MSTF | NA | CA, QR, YU |  |  |  |  |  | + | + | + |  |  |  |  |
| Solanaceae | *Lycianthes sideroxyloides* (Schltdl.) Bitter | - | - | WC | MSDF, MSTF | NA | CA, QR, YU |  |  |  |  | + | + |  | + |  |  |  |  |
| Solanaceae | *Lycianthes sideroxyloides* (Schltdl.) Bitter | Ojo de venado | - | SH | MSDF, MSTF | NA | CA, QR, YU |  |  |  |  |  |  |  | + |  |  |  |  |
| Solanaceae | *Lycium carolinianum* Walter | - | - | SH | CD, CDS, LDF, LIFT | NA | QR, YU |  | + |  | + | + | + |  |  | + | + | + |  |
| Solanaceae | *Nicotiana tabacum* L. | - | - | HE | SV | CU | CA, QR, YU | + | + | + | + | + | + | + | + | + | + | + | + |
| Solanaceae | *Physalis campechiana* L | Tomstillo | Oonop xiiw macho | SH | LDF, RV | NA | CA, QR, YU |  |  |  |  |  |  |  | + | + | + | + | + |
| Solanaceae | *Physalis cinerascens* (Dunal) Hitchc. | Tomatillo | P'aakil | HE | SV | NA | CA, QR, YU | + |  |  |  | + | + |  | + | + | + |  |  |
| Solanaceae | *Physalis pruinosa* L. | - | - | HE | LDF | NA | YU |  |  |  |  |  |  | + | + |  |  |  |  |
| Solanaceae | *Physalis pubescens* L. | Tomatillo | P’aak kanil | HE | LDF, SV | NA | CA, QR, YU | + | + |  |  |  |  |  |  |  |  |  |  |
| Solanaceae | *Schwenckia americana* L. | - | Xayúu lool xiiw | HE | MG, LDF, SV | NA | CA, QR, YU |  | + | + | + |  |  |  | + | + | + | + |  |
| Solanaceae | *Solanum americanum* Mill. | Chilillo | Iik koox | HE | LDF, MG, MSDF, SAV, SV | NA | CA, QR, YU | + | + | + | + | + |  | + | + | + | + | + | + |
| Solanaceae | *Solanum chiapasense* K. E. Roe | - | - | SH | MSDF | NA | QR |  |  |  | + |  |  |  |  |  |  |  |  |
| Solanaceae | *Solanum dasyanthum* Brandegee | - | - | HE | LDF, LIFT | NA | CA, QR, YU |  |  |  |  | + |  |  |  |  |  | + |  |
| Solanaceae | *Solanum diphyllum* L. | Chilillo | Iik k’aax | SH | EVF, MSTF | NA | CA, QR, YU |  |  |  | + | + |  |  |  |  |  |  |  |
| Solanaceae | *Solanum donianum* Walp | Berenjena | chal che’ | HE | CD, LDF, LIFT, MSDF, SV | NA | CA, QR, YU | + |  |  |  | + | + | + |  |  |  | + |  |
| Solanaceae | *Solanum erianthum* D. Don | Lava plato | Chal che | SH | LDF, MSDF, MSTF, SV | NA | CA, QR, YU | + | + | + | + | + |  |  | + | + | + | + | + |
| Solanaceae | *Solanum hirtum* Vahl | Paperas | Puut baalam | SH | SAV, LDF, EVF, SV, MSTF | NA | CA, QR, YU | + |  | + | + | + | + | + | + | + | + | + | + |
| Solanaceae | *Solanum lanceifolium* Jacq. | - | Sikil múuch | WC | AV, CDS, LDF, MSDF, MSTF, SV | NA | CA, QR, YU |  | + |  |  |  | + | + | + |  |  |  |  |
| Solanaceae | *Solanum lycopersicum* L. | Tomate | - | HE | SV | CU | CA, QR, YU |  |  |  |  |  |  |  |  |  | + |  |  |
| Solanaceae | *Solanum mammosum* L. | Ubre de vaca | - | WC | EVF, SV | CU | CA, QR, YU |  |  |  |  |  |  |  |  |  |  |  | + |
| Solanaceae | *Solanum nudum* Dunal | Adano | Boox kúuts | AR | CD, EVF, LDF, MSDF, MSTF, SV | NA | CA, QR, YU |  |  |  |  | + | + | + | + | + | + | + |  |
| Solanaceae | *Solanum rudepannum* Dunal | - | Ts’ay ooch | HE | EVF, LDF, MSDF, MSTF, SV | NA | CA, QR, YU |  | + |  | + |  | + | + | + |  |  |  |  |
| Solanaceae | *Solanum tridynamum* Dunal | - | Kóon ya’ax | HE | CDS, LDF, MSDF, SV | NA | CA, QR, YU |  |  | + | + | + | + | + | + | + |  |  |  |
| Surianaceae | *Suriana maritima* L. | Tabaquillo | Pats’il | SH | CD, CDS | NA | CA, QR, YU | + |  |  |  |  | + | + | + |  |  |  |  |
| Theaceae | *Ternstroemia tepezapote* Schltdl. & Cham. | Manguillo | - | TR | SAV | NA | CA, QR |  |  | + |  | + |  |  |  |  |  |  |  |
| Thymelaceae | *Daphnopsis americana* (Miller) J. R. Johnston. var. *americana* | - | - | SH | RV | NA | QR | + |  |  |  | + |  |  |  |  |  |  | + |
| Thymelaceae | *Daphnopsis mollis* (Schltdl. & Cham.) Standl. | - | - | TR | LDF | NA | QR |  |  |  |  |  |  |  | + |  |  |  |  |
| Typhaceae | *Typha angustifolia* L. | Totora | - | HE | LDF, LIFT, MG, RV | NA | CA, QR, YU | + | + | + | + |  |  |  |  | + | + | + | + |
| Ulmaceae | *Phyllostylon brasiliense* Capan. ex Benth. & Hook. f. | Cerón | Canché | TR | LDF, MSDF, SV | NA | YU | + |  |  | + | + | + |  | + | + | + |  |  |
| Urticaceae | *Cecropia peltata* L. | Guarumbo | K’ooch k’aax | TR | EVF, LDF, MSTF, SV | NA | CA, QR, YU | + |  |  | + | + | + | + | + |  |  | + |  |
| Verbenaceae | *Aloysia virgata* (Ruiz y Pav.) Juss | - | - | SH | LDF, SV | CU | CA, QR, YU |  |  |  | + |  |  | + | + |  |  |  |  |
| Verbenaceae | *Avicennia germinans* (L.) L. | - | - | TR | MG | NA | CA, QR, YU |  |  |  |  |  |  |  |  |  |  |  |  |
| Verbenaceae | *Bouchea prismatica* (L.) Kuntze | Verbena silvestre | - | HE | LDF, MSDF, MSTF, SV | NA | CA, QR, YU | + | + | + | + | + | + | + | + | + | + | + | + |
| Verbenaceae | *Citharexylum hirtellum* Standl. | - | - | SH | LDF, MSDF, MSTF | NA | CA, QR |  |  |  |  | + | + | + | + | + | + |  |  |
| Verbenaceae | *Citharexylum schottii* Greenm. | Palo de violín | Ixim-ché | SH | MSDF | NA | QR, YU | + | + | + | + | + | + | + | + | + | + | + | + |
| Verbenaceae | *Duranta erecta* L | Duranta | K’an pok’ool che’ | SH | CDS, LDF, MSDF, MSTF | NA | CA, QR, YU | + | + | + | + | + | + | + | + | + | + | + | + |
| Verbenaceae | *Lantana camara* L. | Siete colores | Mo'ol peek | SH | CDS, LDF, LDFCC, LIFT, MG, MSDF, MSTF | NA | CA, QR, YU | + | + | + | + | + | + | + | + | + | + | + | + |
| Verbenaceae | *Lantana canescens* Kunth | Orégano k'aax | Xikin juj | SH | LDF, SV | NA | CA, QR, YU |  |  |  |  |  | + | + | + | + | + | + |  |
| Verbenaceae | *Lantana urticifolia* Mill. | - | - | SH | CDS, LDF, LDFCC, LIFT, MG, MSDF, MSTF | NA | QR, YU | + | + | + | + | + | + | + | + | + | + | + | + |
| Verbenaceae | *Lippia alba* (Mill.) N. E. Br. ex Britton & P. Wilson | - | - | SH | LIFT | CU | CA, QR, YU |  |  |  | + |  |  |  |  |  |  |  |  |
| Verbenaceae | *Lippia graveolens* Kunth | Orégano | - | SH | CDS, LDF, MSDF, MSTF, SV | NA | CA, QR, YU | + | + | + | + | + | + | + | + | + | + | + | + |
| Verbenaceae | *Lippia myriocephala* Schltdl. & Cham. | - | - | SH | MSDF, MSTF, SV | NA | CA, YU | + | + | + | + | + | + | + | + | + | + | + | + |
| Verbenaceae | *Petrea volubilis* L. | Tostada de caballo | Oop’tsiimim | WC | CDS, LDF, MSDF, MSTF, SV | NA | CA, QR, YU | + | + | + | + | + | + | + | + | + | + | + |  |
| Verbenaceae | *Phyla dulcis* (Trevir.) Moldenke | - | - | HE | LDF, MSDF, MSTF | NA | CA, QR, YU |  |  |  | + | + | + | + | + | + | + |  |  |
| Verbenaceae | *Phyla nodiflora* (L.) Greene | - | - | HE | CD, CDS, MG, LDF, LIFT, LDFCC, MSDF, MSTF, SV | NA | CA, QR, YU | + | + | + | + | + | + | + | + | + | + | + | + |
| Verbenaceae | *Phyla stoechadifolia* (L.) Small | Pega pega | - | HE | LDF, MSDF, MSTF, SV | NA | CA, QR, YU | + | + | + | + | + | + | + | + | + |  |  |  |
| Verbenaceae | *Priva lappulacea* (L.) Pers. | Pega pega | Ts’a yun t’say | HE | CDS, LDF, LDFCC, MSDF, MSTF, MSTF, SV | NA | CA, QR, YU | + | + | + | + | + | + | + | + | + | + | + | + |
| Verbenaceae | *Stachytarpheta angustifolia* (Mill.) Vahl | - | - | HE | AV, CDS, LDF, LIFT, MSDF, MSTF, SV | NA | CA, QR, YU | + | + | + | + | + | + | + | + | + | + | + | + |
| Verbenaceae | *Stachytarpheta cayennensis* (Rich.) Vahl | - | - | HE | MSDF, MSTF, SV | NA | CA, QR, YU | + | + | + | + | + | + | + | + | + | + | + | + |
| Verbenaceae | *Stachytarpheta frantzii* Pol. | Cola de mico | - | HE | LDF, LIFT, MSDF, MSTF, SV | NA | CA, QR, YU | + | + | + | + | + | + | + | + | + | + | + | + |
| Verbenaceae | *Stachytarpheta miniacea* Moldenke | - | - | HE | MSDF, MSTF, SV | NA | CA, QR, YU | + | + | + | + | + | + | + | + | + | + | + | + |
| Verbenaceae | *Tamonea curassavica* (L.) Pers. | Cabeza arriera chan | Aak’ uuch | HE | CDS, LDF, LIFT, LDFCC, MSDF, MSTF, SV | NA | CA, QR, YU | + | + | + | + | + | + | + | + | + | + | + | + |
| Violaceae | *Corynostylis arborea* (L.) S. F. Blake | - | - | WC | RV | NA | CA, QR |  |  |  |  |  |  |  |  |  | + | + |  |
| Violaceae | *Orthion subsessile* (Standl.) Steyerm. & Standl. | Botoncillo | Chak tuuk anil | TR | LDF, MSDF, MSTF, SV | NA | CA | + | + | + | + |  |  |  |  |  |  |  | + |
| Violaceae | *Rinorea guatemalensis* (S. Watson) Bartlett | - | - | SH | EVF | NA | CA, QR |  |  |  |  |  |  |  | + | + |  |  |  |
| Violaceae | *Rinorea hummelii* Sprague | - | - | SH | EVF, MSTF | NA | CA, QR |  | + | + | + | + | + |  |  |  |  |  |  |
| Vitaceae | *Ampelocissus erdwendbergii* Planch. | - | - | WC | MSDF, MSTF | NA | QR, YU |  |  |  | + | + |  |  |  |  |  |  |  |
| Vitaceae | *Cissus biformifolia* Standl. | - | - | WC | EVF, LDF, MSDF | NA | CA, QR, YU |  |  |  |  |  | + | + | + | + | + | + |  |
| Vitaceae | *Cissus cacuminis* Standl. | - | - | WC | SV | NA | CA, QR |  |  |  |  |  |  | + |  |  |  |  |  |
| Vitaceae | *Cissus erosa* Rich. subsp. *erosa* | - | - | WC | LDF | NA | YU | + | + | + | + | + | + | + | + | + | + | + | + |
| Vitaceae | *Cissus microcarpa* Vahl | - | - | WC | LDF, LIFT, MSDF | NA | CA, QR, YU |  |  |  |  |  |  | + | + | + |  |  |  |
| Vitaceae | *Cissus trifoliata* (L.) L. | - | Bolon tib ib | WC | CD, CDS, LDF, MG, MSDF, SV | NA | CA, QR, YU |  | + | + |  | + | + | + | + | + |  |  |  |
| Vitaceae | *Cissus verticillata* (L.) Nicolson & C. E. Jarvis var. *verticilata* | - | - | WC | LDF, LIFT, MSDF, MSTF, SV | NA | CA, QR, YU |  | + | + |  |  | + | + | + | + | + | + |  |
| Vitaceae | *Vitis bourgaeana* Planch. | - | - | WC | MSDF | NA | CA, QR |  |  | + |  |  |  |  |  |  |  |  |  |
| Vitaceae | *Vitis tiliifolia* Humb. & Bonpl. ex Roem. & Schult. | Uvas de monte | Xta’kanil | WC | LDF, MSDF | NA | CA, QR, YU |  |  | + |  |  |  |  |  |  |  |  |  |
| Zygophyllaceae | *Guaiacum sanctum* L. | Palo santo | - | SH | LDF, LDFCC, MSDF | NA | CA, QR, YU | + | + | + | + | + | + | + | + | + | + | + | + |
| Zygophyllaceae | *Kallstroemia maxima* (L.) Hook. & Arn. | - | Xich’iil aak | HE | SV | NA | CA, QR, YU |  | + | + | + | + | + | + | + | + | + | + | + |
| Zygophyllaceae | *Tribulus cistoides* L. | Abrojo | Chan koj xnuk | HE | CD, CDS, MG | NA | CA, QR, YU |  |  |  | + |  |  |  |  |  |  |  |  |
