# Supplementary figures and images for: A review of the melliferous flora of Yucatan peninsula, Mexico, on the basis for the honey production cycle
Source: J Ethnobiol Ethnomed. 2024 Mar 25;20:40. doi: 10.1186/s13002-024-00681-0 (PMC10964660; doi:10.1186/s13002-024-00681-0)

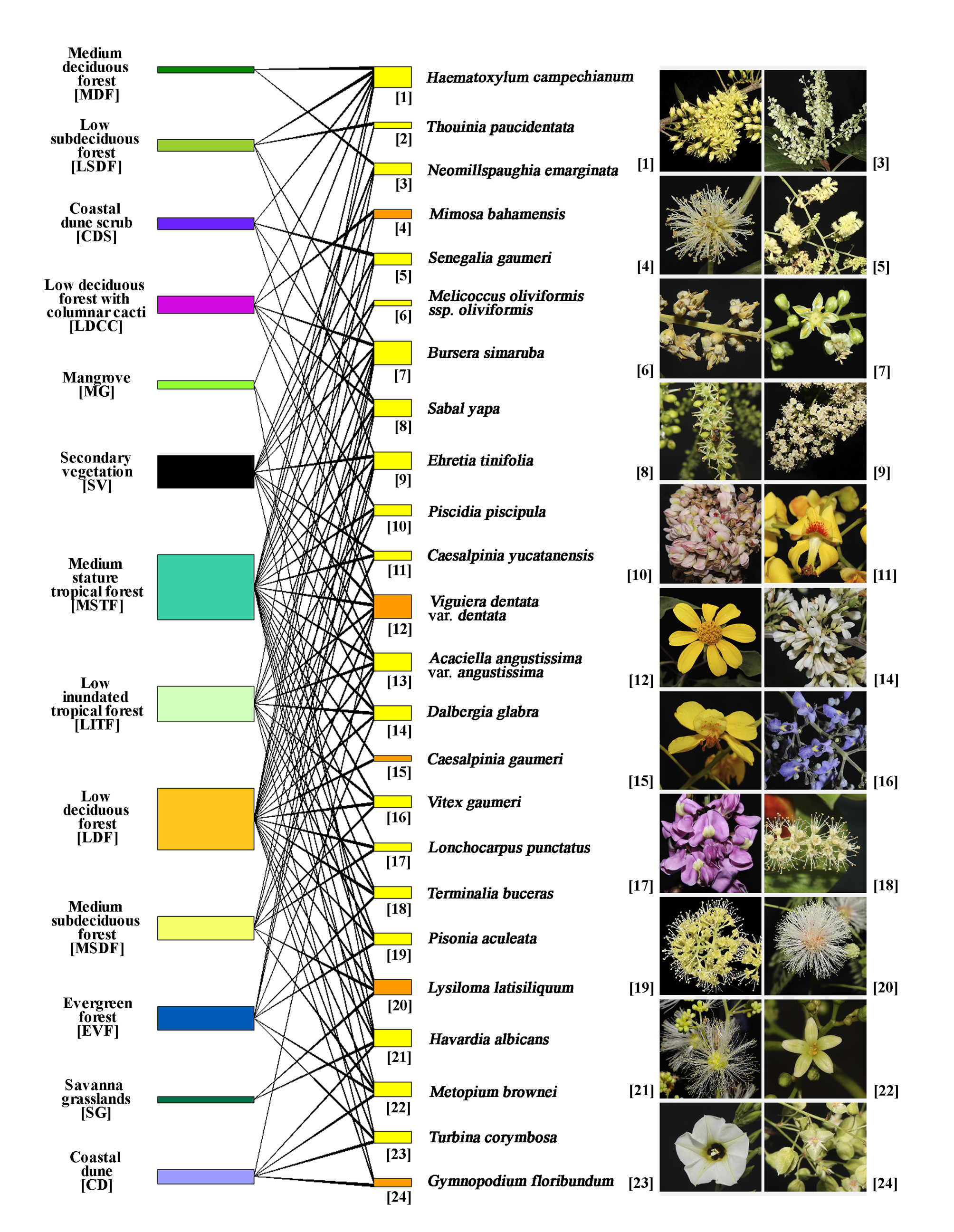

Supplement: Supplementary file 2 — Additional file 2. Figure S1: Network of interaction between the types of vegetation and most important species for beekeeping in the Yucatan peninsula. Number below the boxes represent the numbers of the images to indicate their scientific name. [file 13002_2024_681_MOESM2_ESM.tif]

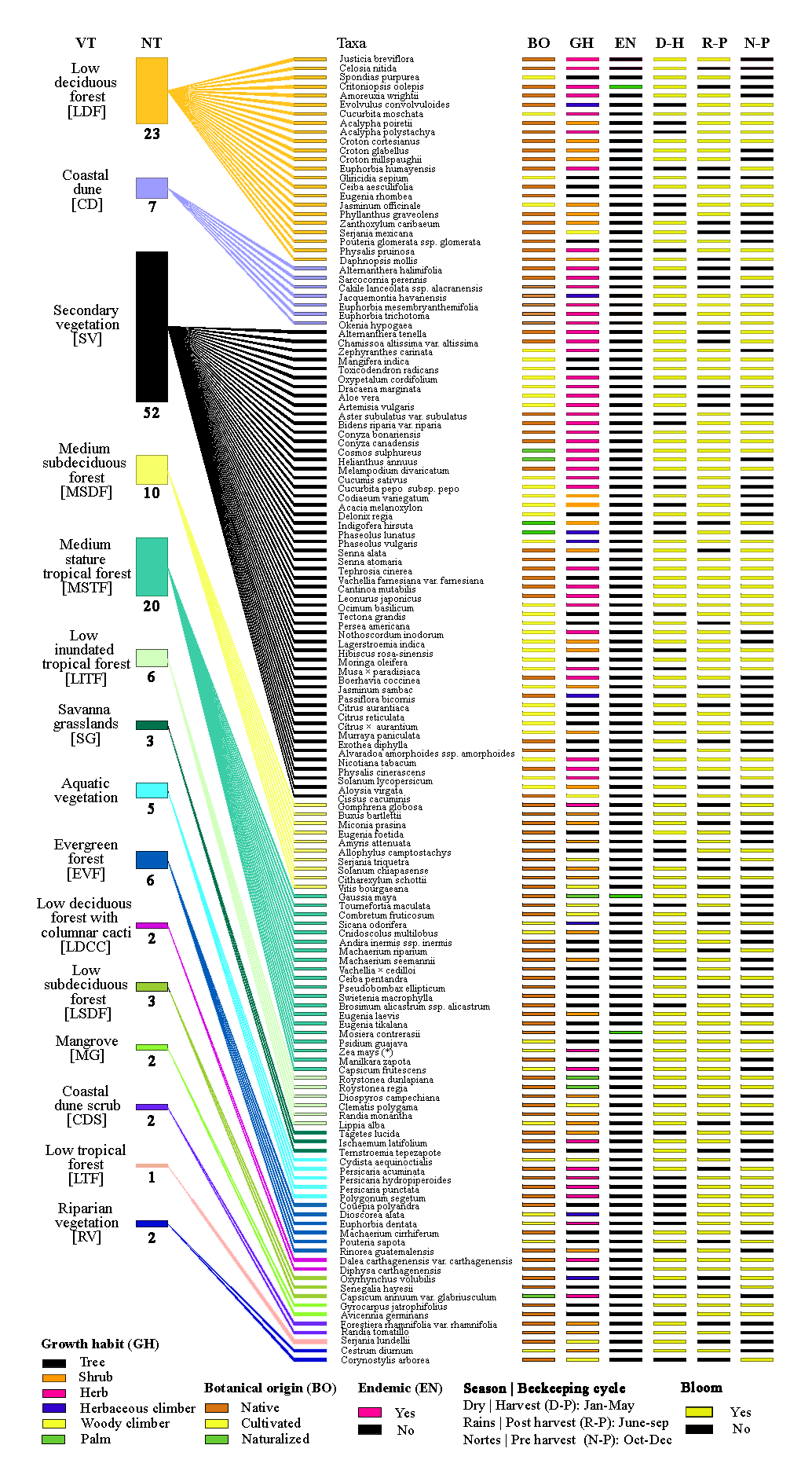

Supplement: Supplementary file 3 — Additional file 3. Figure S2: Interaction networks between vegetation types and species recorded as unique to these ecosystems (including growth habit, botanical origin, endemism and flowering period with respect to the climatic season and beekeeping cycle). NT = Number of taxa by type of vegetation. [file 13002_2024_681_MOESM3_ESM.tif]
